# Six mitophagy-related hub genes as peripheral blood biomarkers of Alzheimer’s disease and their immune cell infiltration correlation

**DOI:** 10.3389/fnins.2023.1125281

**Published:** 2023-05-18

**Authors:** Kun Zhao, Yinyan Wu, Dongliang Zhao, Hui Zhang, Jianyang Lin, Yuanwei Wang

**Affiliations:** ^1^Department of Neurology, Affiliated People's Hospital of Jiangsu University, Zhenjiang, Jiangsu, China; ^2^Fujian Center for Safety Evaluation of New Drug, Fujian Medical University, Fuzhou, Fujian, China; ^3^Department of General Surgery, Affiliated People's Hospital of Jiangsu University, Zhenjiang, Jiangsu, China; ^4^Department of Neurology, Shuyang Hospital Affiliated to Xuzhou Medical University, Shuyang, Jiangsu, China

**Keywords:** Alzheimer’s disease, hub gene, mitophagy, bioinformatics analysis, blood biomarker, immune infiltration

## Abstract

**Background:**

Alzheimer’s disease (AD), a neurodegenerative disorder with progressive symptoms, seriously endangers human health worldwide. AD diagnosis and treatment are challenging, but molecular biomarkers show diagnostic potential. This study aimed to investigate AD biomarkers in the peripheral blood.

**Method:**

Utilizing three microarray datasets, we systematically analyzed the differences in expression and predictive value of mitophagy-related hub genes (MRHGs) in the peripheral blood mononuclear cells of patients with AD to identify potential diagnostic biomarkers. Subsequently, a protein–protein interaction network was constructed to identify hub genes, and functional enrichment analyses were performed. Using consistent clustering analysis, AD subtypes with significant differences were determined. Finally, infiltration patterns of immune cells in AD subtypes and the relationship between MRHGs and immune cells were investigated by two algorithms, CIBERSORT and single-sample gene set enrichment analysis (ssGSEA).

**Results:**

Our study identified 53 AD- and mitophagy-related differentially expressed genes and six MRHGs, which may be potential biomarkers for diagnosing AD. Functional analysis revealed that six MRHGs significantly affected biologically relevant functions and signaling pathways such as IL-4 Signaling Pathway, RUNX3 Regulates Notch Signaling Pathway, IL-1 and Megakaryocytes in Obesity Pathway, and Overview of Leukocyteintrinsic Hippo Pathway. Furthermore, CIBERSORT and ssGSEA algorithms were used for all AD samples to analyze the abundance of infiltrating immune cells in the two disease subtypes. The results showed that these subtypes were significantly related to immune cell types such as activated mast cells, regulatory T cells, M0 macrophages, and neutrophils. Moreover, specific MRHGs were significantly correlated with immune cell levels.

**Conclusion:**

Our findings suggest that MRHGs may contribute to the development and prognosis of AD. The six identified MRHGs could be used as valuable diagnostic biomarkers for further research on AD. This study may provide new promising diagnostic and therapeutic targets in the peripheral blood of patients with AD.

## 1. Introduction

Alzheimer’s disease (AD) is a common, progressive, and complex neurodegenerative disorder that causes cognitive decline, memory loss, and difficulty performing daily tasks (Heckmann et al., 2020). Globally, AD poses a huge threat to people’s health and a significant economic burden to society (Dumitrescu et al., 2020). Thus far, the pathogenesis of AD remains unknown, and there is no definitive treatment. Some molecules correlate with AD progression and cognitive decline; the identification of molecular changes and biological processes connected to AD can increase our understanding of AD pathogenesis and provide biomarkers for AD.

Pathological hallmarks of AD are aggregated amyloid-β (Aβ) protein in senile plaques and aggregated tau protein in neurofibrillary tangles (Liang et al., 2016). However, the molecular mechanisms regulating AD development *via* Aβ, tau, or other factors are poorly understood. Over the past few decades, an increasing number of therapies and immunotherapies, such as vaccines and drugs targeting Aβ protein, tau protein, or AD-related genes, have been developed. The effectiveness of these targeted therapies has been demonstrated in some patient populations and animal models of AD (Town et al., 2008; Sevigny et al., 2016; Congdon and Sigurdsson, 2018; Xiong et al., 2021; Jung et al., 2022); however, it is always challenging to translate these results into humans safely and effectively (Town et al., 2008; Lemere, 2013; Xiong et al., 2021). Thus, it is crucial to identify novel immunological diagnostic and therapeutic AD markers.

Healthy and active mitochondria are essential for neuronal function (Chakravorty et al., 2019; Pradeepkiran and Reddy, 2020). The accumulation of damaged mitochondria and mitochondrial dysfunction are early marker events and core participants in the process of AD (Chakravorty et al., 2019; Fang, 2019). In AD neurons, mitochondrial dysfunction is related to mitochondrial dynamics, biogenesis, and mitophagy (Grimm and Eckert, 2017; Kerr et al., 2017). Mitophagy, also called selective autophagy, is a selective degradation process that gradually accumulates defective mitochondria through autophagy. It is a key mitochondrial quality control system that helps neurons maintain health and function by removing unnecessary and damaged mitochondria. In other words, dysfunctional mitochondria and dysfunctional mitophagy in neurons are closely related to the occurrence of AD. Various proteins related to mitophagy were found to be changed in AD neurons (Rai et al., 2020; Mary et al., 2023). Recent studies (Fang et al., 2019; Morton et al., 2021; Pradeepkiran et al., 2022) from animal and cell models of AD and sporadic late-onset AD showed that impaired mitophagy triggered Aβ and tau protein accumulation by increasing oxidative damage and cell energy deficiency, leading to synaptic dysfunction and cognitive impairment. Moreover, these processes can compromise mitophagy (Fang et al., 2019; Morton et al., 2021; Pradeepkiran et al., 2022). Therefore, interventions that support mitochondrial health or stimulate mitophagy may prevent the neurodegenerative process of AD (Kerr et al., 2017; Fang et al., 2019). Accordingly, by removing defective mitochondria in AD through mitophagy targeting, it might be possible to intervene therapeutically (Wang et al., 2021; Pradeepkiran et al., 2022; Sharma et al., 2022; Xie et al., 2022). Nevertheless, the association of mitophagy with AD pathology and AD-related changes in immune system effectiveness is not fully explained and requires further investigation.

In recent decades, researchers have been interested in finding new biomarkers or models to early identify metabolic risk abnormalities. The progression and prognosis of AD can be affected by many genetic or epigenetic alterations (Karch and Goate, 2015; Efthymiou and Goate, 2017). Familial AD accounts for 5–10% of all AD cases. Pathogenic mutations in genes like *APP*, *PSEN1*, and *PSEN2* are found in approximately 15–20%, 70–80, and 5% of patients with familial AD, respectively (Ryan and Rossor, 2010; Williams, 2011). Apolipoprotein E (*APOE*), as the most important susceptible gene known, may play an important role in the predisposition to sporadic AD; the *APOE4* gene is associated with late-onset AD and contributes to the development of neurofibrillary tangles and Aβ senile plaques (Corder et al., 1993; Poirier et al., 1993). *TREM2* is also a very important gene and encodes the protein, triggering receptor expressed on myeloid cells 2 (TREM2); it is expressed by microglia, the resident immune cells of the brain, and strongly affects the lifelong risk of AD (Roussos et al., 2015; Ulrich et al., 2017). Some other genes such as *CR1*, *SPI1*, *MS4As*, *ABCA7*, *CD33*, and *INPP5D* (Roussos et al., 2015) involved in different biological processes are expressed by microglia as well. *APOE*, *CLU*, and *ABCA7* may be related to lipid metabolism; *ABCA7*, *CD33*, *CR1*, *CLU*, and *EPHA1* may be associated with immune system function (Reitz et al., 2013; Villegas-Llerena et al., 2016); *PICALM*, *BIN1*, *CD33*, and *CD2AP* may be related to cell membrane function including endocytosis (Villegas-Llerena et al., 2016). In addition, polymorphisms of *CLU*, *SORL1*, and *MS4A4A* genes also affect AD-related biomarkers (mainly Aβ, tau, and phosphorylated tau proteins) within the cerebrospinal fluid (Elias-Sonnenschein et al., 2013). However, research on AD is complex, and more experiments are needed to break through the treatment bottleneck of AD. The advances in bioinformatics enable independent studies to identify biomarkers. Numerous genes and loci can be analyzed using bioinformatics to uncover potential biological pathways in AD (Suh et al., 2019).

Our study utilized the Gene Expression Omnibus (GEO) database of the National Center for Biotechnology Information to analyze mitophagy-related differential expressed genes (MRDEGs), do functional enrichment analyses, construct a diagnostic model, determine those that play key roles in AD, and identify possible biomarkers in the peripheral blood and their associated immune cell infiltration. Furthermore, we compared the mitophagy-related hub genes (MRHGs) and immune patterns of patients with AD with those of controls. However, the purpose of this research was to investigate AD biomarkers related to mitophagy and their immune cell infiltration correlation in the peripheral blood.

## 2. Materials and methods

### 2.1. Data retrieval

The AD-related datasets GSE110226 (Kant et al., 2018; Stopa et al., 2018), GSE1297 (Blalock et al., 2004), and GSE63060 (Sood et al., 2015) were downloaded from the GEO database through the R package GEOquery (Davis and Meltzer, 2007). The control samples of all three datasets were obtained from healthy individuals. In this study, we included 7 AD and 6 control samples from GSE110226, 22 AD and 9 control samples from GSE1297 (Supplementary Table S1), and 145 AD and 104 control samples from GSE63060. The batch effects of the datasets GSE110226 and GSE1297 were removed using the R package *sva* (Leek et al., 2012) to obtain an integrated GEO dataset, i.e., combined datasets (CDs) including 29 AD and 15 control samples. Finally, the CDs and GSE63060 were standardized using the R package *limma*, and the annotation probes were standardized and normalized.

**Figure 1:**
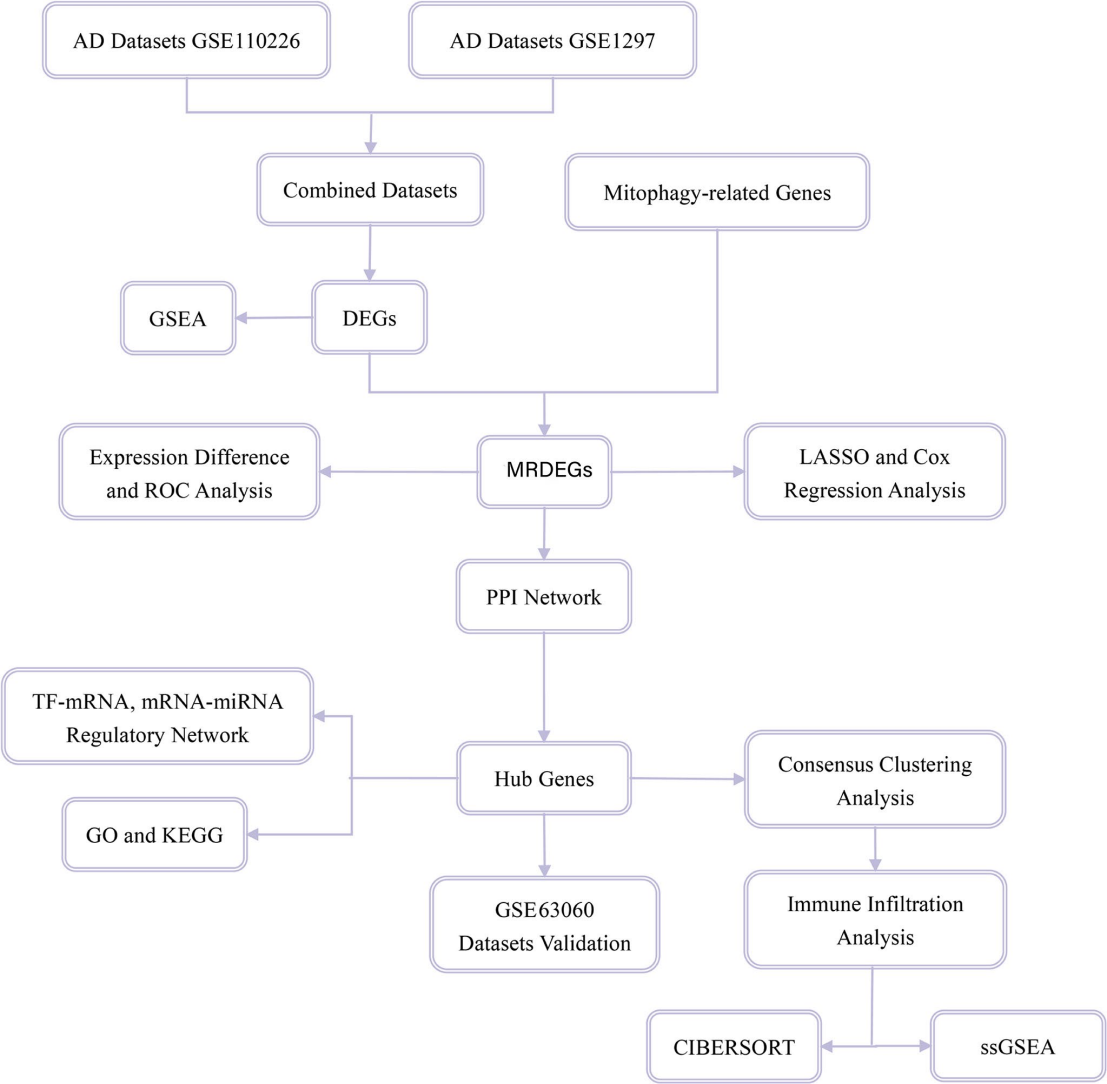
Flow chart for the comprehensive analysis of MRDEGs. MRDEG, mitophagy-related differentially expressed gene.

Mitophagy-related genes (MRGs) were collected using the GeneCards database (Stelzer et al., 2016), which provides comprehensive information about human genes. In addition, MRGs in the published literature (Zhuo et al., 2022) were obtained on the PubMed website using the term “mitophagy-related genes.” A total of 2,414 MRGs were obtained after combining the results and removing duplicates. A flow diagram of the database search is shown in Figure 1.

### 2.2. Differentially expressed genes related to AD

According to the sample grouping of the CDs, samples were divided into the AD and control groups. Differential analysis of genes in different groups was performed using the R package differential gene expression analysis based on the negative binomial distribution (*DESeq2*) (Love et al., 2014). DEGs with logFC>0.5 and *p* < 0.05 were considered statistically significant. Among these, genes with logFC>0.5 and *p* < 0.05 were considered upregulated, and genes with logFC<0.5 and *p* < 0.05 were considered downregulated.

To obtain MRDEGs associated with AD, all DEGs with logFC>0.5 and *p* < 0.05 obtained by differential analysis in the CDs and MRGs were intersected and plotted to obtain MRDEGs. The results of the differential analysis were plotted using the R package *ggplot2*, the heatmap was drawn using the R package *pheatmap*, and chromosome mapping was performed using the R package *RCircos* (Zhang et al., 2013).

### 2.3. Receiver operating characteristic curve

The ROC curve (Park et al., 2004) is a comprehensive index reflecting continuous variables of sensitivity and specificity. The relationship between sensitivity and specificity is reflected by the composition method. The area under the ROC curve (AUROC) is generally between 0.5 and 1. The closer the AUROC value is to 1, the better the diagnostic effect. The AUC values were considered low, medium, or high accuracy for ranges 0.5–0.7, 0.7–0.9, and > 0.9, respectively. The R package *survivalROC* was used to plot the ROC curves of MRDEGs, as well as the survival times and statuses of patients with AD.

### 2.4. Construction of the diagnostic model of MRDEGs

In order to obtain a diagnostic model of MRDEGs in the AD datasets, the R package glmnet (Engebretsen and Bohlin, 2019) with set.seed(2) and family = “binary” as parameters was used to perform least absolute shrinkage and selection operator (LASSO) regression analysis based on MRDEGs. To avoid overfitting, the operating cycle is 1,000. LASSO regression is often used to construct a prognostic model, which is based on linear regression and by adding a penalty term (lambda × absolute value of the slope) reduces the overfitting of the model and improves the generalization ability of the model. The results of LASSO regression analysis were visualized utilizing the diagnostic model and variable trajectory diagrams and the molecular expression of each gene in the MRDEG diagnostic model was displayed in a forest plot.

Thereafter, MRDEGs were screened by LASSO regression analysis, and univariate and multivariate Cox regression analyses were performed to construct a multivariate Cox regression model. Nomogram (Wu et al., 2020) is a graph that uses a cluster of disjoint line segments to represent the functional relationship between multiple independent variables in the plane rectangular coordinate system. Based on these results, nomograms were drawn using the R package *rms*. Next, a calibration analysis was performed, and a calibration curve was generated to evaluate the accuracy and resolution of the nomograms. Decision curve analysis (Van Calster et al., 2018) is a simple method to evaluate clinical prediction models, diagnostic tests, and molecular markers. Finally, the accuracy and resolution of the multivariate Cox regression model were evaluated using the R package *ggDCA* to draw the decision curve analysis map.

### 2.5. Protein–protein interaction network

Protein protein interaction (PPI) network is composed of proteins and proteins through the interaction between them. The STRING database (Szklarczyk et al., 2019) was used to construct the PPI network related to the MRDEGs with a minimum required interaction score of medium confidence (0.400) as the standard, and the Cytoscape software (Shannon et al., 2003) was used to visualize the PPI network model.

In addition, five algorithms in the CytoHubba (Chin et al., 2014) plug-in were applied: maximum neighborhood component, degree, maximal clique centrality, closeness, and edge percolated component (Yang et al., 2019; Liu et al., 2022). In the PPI network, the scores of the MRDEGs were initially calculated, and then the MRDEGs were arranged in the order of their scores. Finally, the genes of the five algorithms were collected and analyzed by drawing the Venn diagram. The intersecting genes of the algorithms were considered hub genes related to mitophagy.

### 2.6. Construction of transcription factor-mRNA and mRNA-miRNA regulatory networks

Transcription factors (TFs) control gene expression through interaction with a target gene (mRNA) in the post-transcriptional stage. By retrieving TFs from the ChIPBase database (Zhou et al., 2017), the regulatory effects of TFs on MRHGs were analyzed, and the TF-mRNA regulatory network was visualized using the Cytoscape software.

miRNAs play an important regulatory role in the process of biological development and evolution. They are able to regulate multiple target genes; the same target gene can be regulated by multiple miRNAs. To analyze the relationship between MRHGs and miRNAs, miRNAs related to MRHGs were obtained from the StarBase database (Li et al., 2014). Finally, the mRNA-miRNA regulatory network was visualized using the Cytoscape software.

### 2.7. Gene function enrichment analysis, pathway enrichment analysis, and gene set enrichment analysis

Gene Ontology (GO) analysis (Mi et al., 2019) is a common method for large-scale functional enrichment studies, including biological processes (BPs), molecular functions (MFs) and cell components (CCs). Kyoto Encyclopedia of Genes and Genomes (KEGG) (Kanehisa and Goto, 2000) is a widely used database that stores information about genomes, biological pathways, diseases and drugs. GO and KEGG pathway annotation of MRHGs was analyzed using the R package *clusterProfiler* (Yu et al., 2012). The entry screening criteria were *p* < 0.05 and a false detection rate (q)-value of <0.05, which were considered statistically significant. The value of *p* was corrected using the Benjamini–Hochberg procedure.

Gene Set Enrichment Analysis (GSEA) (Subramanian et al., 2005) was used to evaluate the distribution trend of genes in a predefined gene set in the gene table sorted by the degree of correlation with phenotype, so as to judge their contribution to phenotype. In this study, genes in the CDs were first divided into two groups with high and low phenotypic correlations according to the phenotypic correlation ranking. Thereafter, all DEGs in the two groups with high and low phenotypic correlations were enriched using the R package *clusterProfiler*. The genes were analyzed by GSEA. We retrieved the c2.cp.v7.2.symbols.gmt gene set from the Molecular Signatures database (Liberzon et al., 2011); the screening criteria for significant enrichment were *p* < 0.05 and *q*-values of <0.05.

### 2.8. Molecular subtype construction of MRHGs

Consistency clustering (Lock and Dunson, 2013) refers to multiple iterations of subsamples of a dataset. It provides the index of clustering stability and parameter decision by using subsamples to induce sampling variability. The consensus clustering method using the R package *ConsensusClusterPlus* (Wilkerson and Hayes, 2010) was employed to identify different disease subtypes of AD based on MRHGs.

### 2.9. Analysis of immune cell infiltration

Using CIBERSORT algorithms (Newman et al., 2015) and the LM22 characteristics gene matrix, the samples with output *p*-values of <0.05 were filtered to obtain the immune cell infiltration matrix. The data were then filtered for immune cell enrichment scores greater than zero. Finally, the specific results of the immune cell infiltration matrix were obtained. A histogram was drawn using *ggplot2* to show the distribution of 22 types of immune cell infiltrates in different subtypes of AD samples; the correlation heatmap was drawn using *pheatmap* to illustrate the correlation analysis results of the 22 immune cell types with MRHGs in different AD subtypes.

The relative abundance of each infiltrating immune cell type was quantified using single-sample GSEA (ssGSEA) algorithms (Coscia et al., 2018). First, the types of infiltrating immune cells were labeled, such as activated CD8+ T cells, activated dendritic cells, γδ T cells, natural killer cells, regulatory T cells, and other human immune cell subtypes. Second, the enrichment score calculated by ssGSEA was used to express the relative abundance of each immune cell type in each sample. Finally, *ggplot2* was used to display the distributions of infiltrating immune cells in different disease subtypes of AD samples; *pheatmap* was used to draw a correlation heatmap that shows the results of the correlation analysis between immune cells and MRHGs in different AD subtypes.

### 2.10. Statistical analysis

All data processing and analysis in this article are based on R software version 4.1.2. Continuous variables are presented as mean ± standard deviation. The Wilcoxon rank sum test was used for comparison between two groups; the Kruskal–Wallis test was used for comparisons of three groups or more. The chi-square test or Fisher’s exact test was used to compare and analyze statistical significance between two groups of categorical variables. Unless otherwise specified, correlation coefficients between different molecules were calculated using Spearman’s correlation analysis, and statistical significance was set at *p* < 0.05.

## 3. Results

### 3.1. Analysis of AD-related DEGs

**Figure 2:**
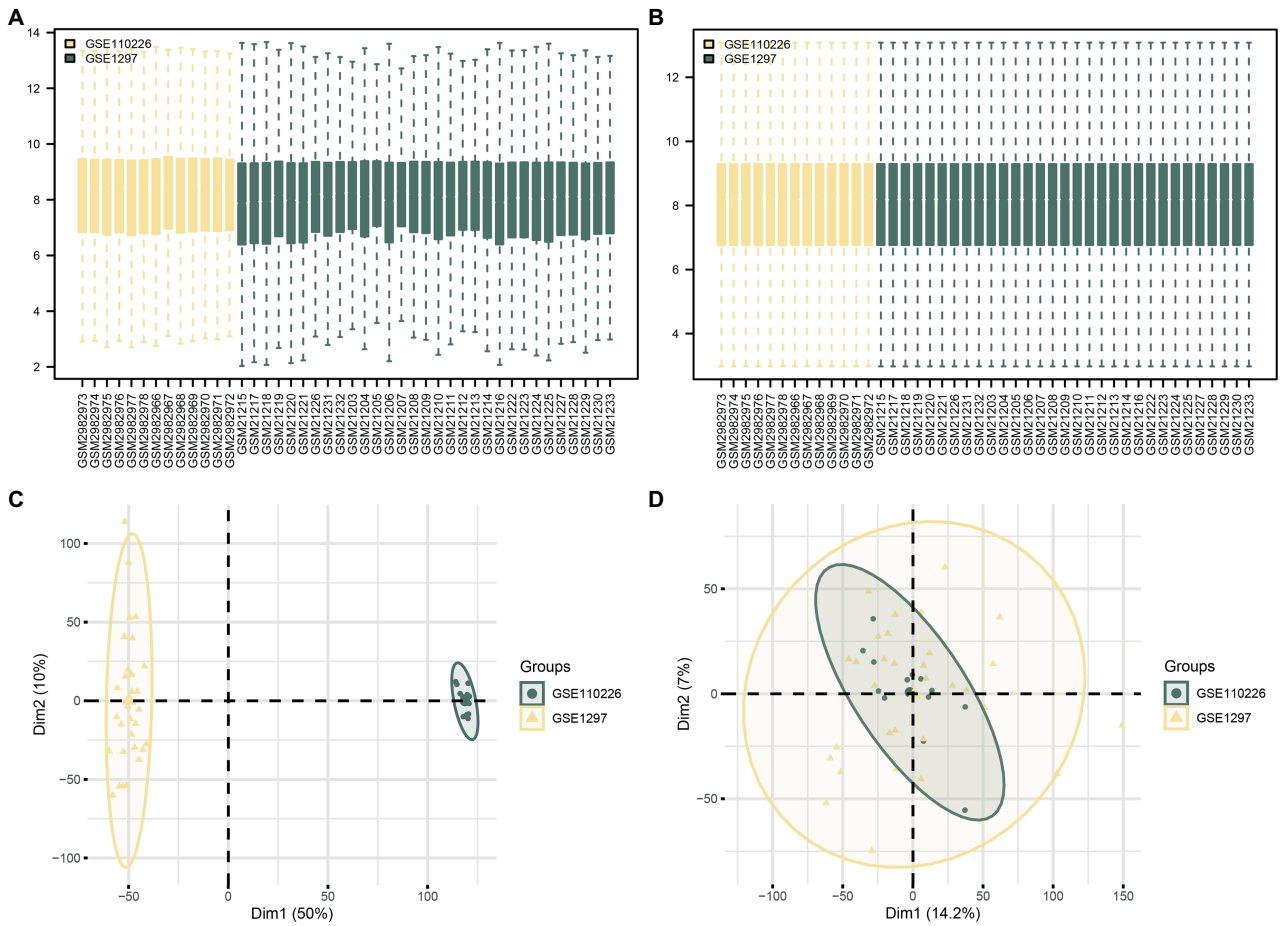
Batch effects removal of GSE110226 and GSE1297. **(A)** Distribution boxplot of datasets before batch processing. **(B)** Distribution boxplot of CDs after batch processing. **(C)** PCA diagram of datasets before batch processing. **(D)** PCA diagram of CDs after batch processing. DEG, differentially expressed gene; MRDEG, mitophagy-related differentially expressed gene; CDs, combined datasets; PCA, principal component analysis.

First, the R package *sva* was used to remove batch effects from the AD datasets GSE110226 and GSE1297 and obtain CDs. The datasets before and after batch effect removal were compared using a distribution box diagram and principal component analysis (PCA) (Figures 2A–D). These results showed that the batch effect of the samples in the AD dataset was basically eliminated by this procedure.

**Figure 3:**
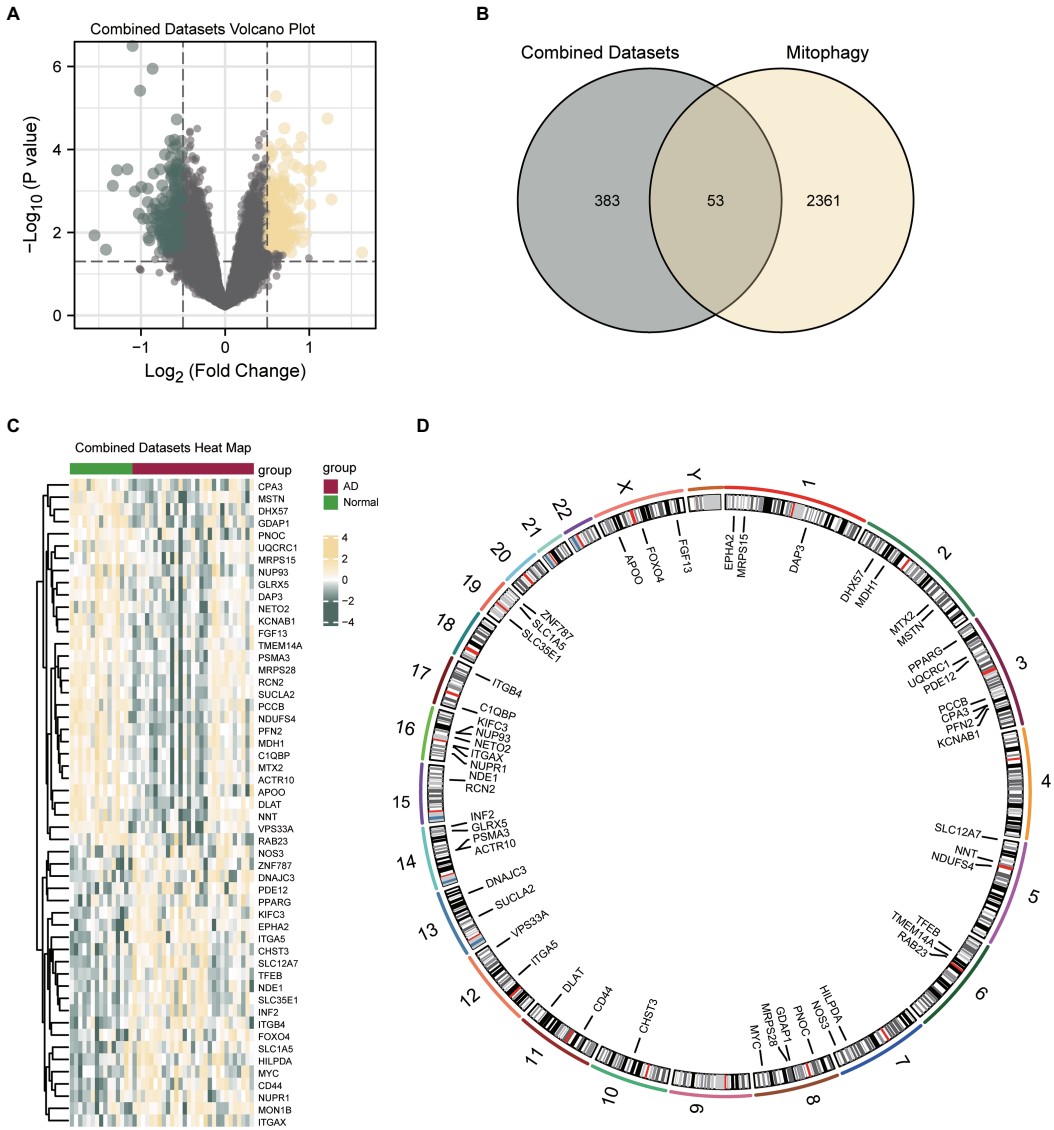
Differential gene expression analysis of AD. **(A)** Volcanic map of differential gene analysis between AD and control groups in CDs. **(B)** Venn diagram of DEGs and MRDEGs in CDs. **(C)** Correlation heat map of MRDEGs in CDs. **(D)** Chromosome mapping of MRDEGs. AD, Alzheimer’s disease; DEG, differentially expressed gene; MRDEG, mitophagy-related differentially expressed gene; CDs, combined datasets.

Then, the data from the CDs were divided into the control and AD groups. To analyze the intergroup differences in gene expression values in the AD dataset, the R package *DESeq2* was used to perform a differential analysis on the CDs of the two data groups. The CDs contained 436 DEGs that met the threshold of logFC>0.5 and *p* < 0.05. Of these, 212 genes were upregulated (logFC>0.5, *p* < 0.05) and 224 downregulated (logFC<0.5, *p* < 0.05), and a volcano map was drawn accordingly (Figure 3A). To identify MRDEGs, all DEGs with logFC>0.5 and *p* < 0.05 were intersected with MRGs (Supplementary Table S2). A total of 53 MRDEGs were obtained, which are illustrated in the Venn diagram in Figure 3B. Specific gene information is presented in Supplementary Table S3. According to the intersection results, differences in MRDEG expression between different CD sample groups were analyzed and displayed in a heatmap (Figure 3C) by using the R package *pheatmap*. Finally, the positions of the identified 53 MRDEGs on human chromosomes were analyzed using the R package *RCircos*, and their chromosome mappings were displayed (Figure 3D; Supplementary Table S4).

### 3.2. Correlation analysis of MRDEGs

**Figure 4:**
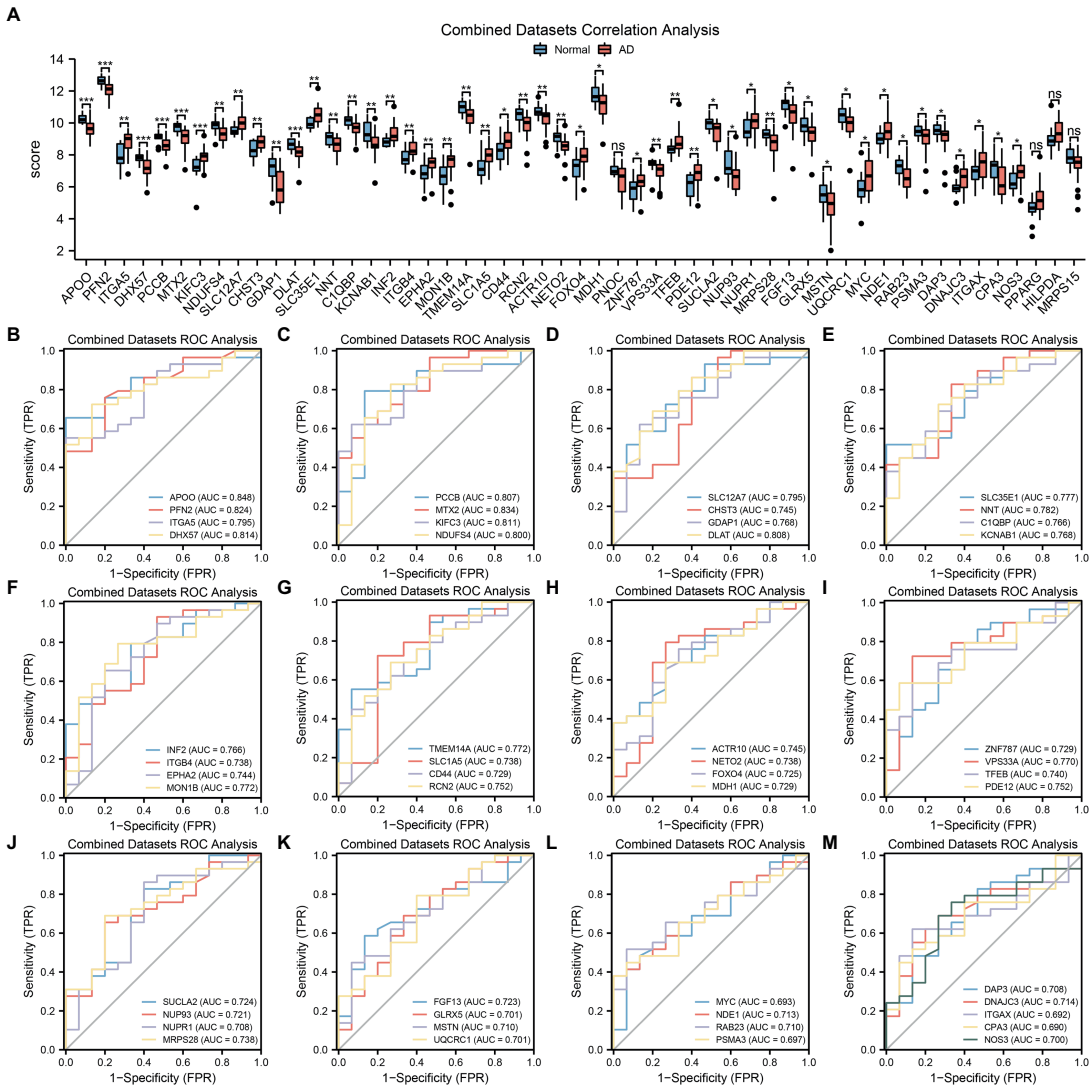
MRDEGs correlation and ROC curve analysis. **(A)** Comparison histogram of the results of differential expression analysis of MRDEGs in CDs. **(B–M)** ROC curves of 49 MRDEGs in CDs. ns, *p* value ≥ 0.05, *p value < 0.05, **p value < 0.01, ***p value < 0.001. AUC at 0.5–0.7 has a low accuracy, while AUC at 0.7–0.9 has a certain accuracy. MRDEG, mitophagy-related differentially expressed gene; ROC, receiver operating characteristic; CDs, combined datasets.

To further explore the differences in MRDEG expression in the AD dataset, a histogram based on grouping and comparison was generated (Figure 4A). It shows the differential expression of the 53 MRDEGs in the AD and control groups in the CDs. The expression levels of 49 MRDEGs were significantly different (Supplementary Table S4). Of these MRDEGs, *APOO*, *PFN2*, *DHX57*, *PCCB*, *MTX2*, *KIFC3*, and dihydrolipoamide S-acetyltransferase (*DLAT*) were significantly different between the AD and control groups (*p* < 0.001); *ITGA5*, *NDUFS4*, *SLC12A7*, *CHST3*, *GDAP1*, *SLC35E1*, *NNT*, *C1QBP*, *KCNAB1*, *INF2*, *ITGB4*, *EPHA2*, *MON1B*, *TMEM14A*, *SLC1A5*, *RCN2*, *ACTR10*, *NETO2*, *VPS33A*, *TFEB*, *PDE12*, and *MRPS28* were highly significantly different (*p* < 0.01); and *CD44*, *FOXO4*, *MDH1*, *ZNF787*, succinate-CoA ligase ADP-forming subunit β (*SUCLA2*), *NUP93*, *NUPR1*, *FGF13*, *GLRX5*, *MSTN*, *UQCRC1*, *MYC*, *NDE1*, *RAB23*, *PSMA3*, *DAP3*, *DNAJC3*, integrin subunit alpha X CD11c (*ITGAX*), *CPA3*, and *NOS3* were significantly different between the studied groups (*p* < 0.05). The expression levels of the remaining genes, including *PNOC*, *PPARG*, *HILPDA*, and *MRPS15*, were not significantly different (Supplementary Table S4).

Next, the ROC curves of the abovementioned 49 MRDEGs were drawn (Figures 4B–M). The ROC curves of 45 MRDEGs revealed a medium correlation with different groups (0.7 < AUC < 0.9; Supplementary Table S4), whereas those of the other four MRDEGs, namely *MYC*, *PSMA3*, *ITGAX*, and *CPA3*, showed a low correlation with different groups (0.5 < AUC < 0.7; Supplementary Table S4).

### 3.3. Construction of the diagnostic model of the MRDEGs

**Figure 5:**
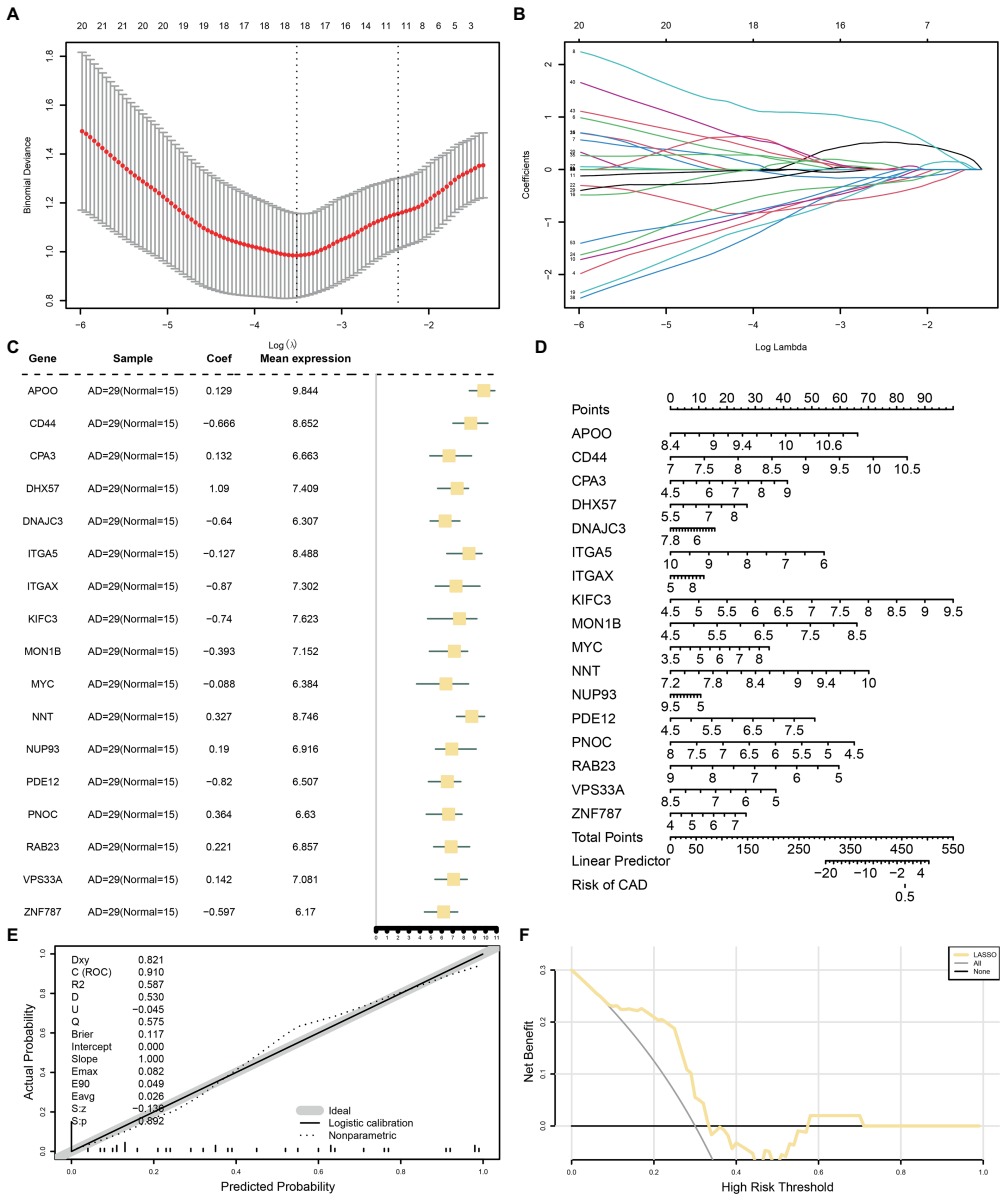
LASSO and cox regression analysis for CDs. **(A)** Diagnostic model of MRDEGs in AD datasets. **(B)** Variable trajectories of MRDEGs in the LASSO diagnostic model of AD. **(C)** Forest map of MRDEGs in the LASSO diagnostic model of AD. **(D)** Nomogram diagram of MRDEGs in Cox regression model. **(E)** Calibration curves of MRDEGs in Cox regression model. **(F)** DCA diagram of MRDEGs in Cox regression model. The ordinate is the net income, and the abscissa is the probability threshold or threshold probability. LASSO, least absolute shrinkage and selection operator; CDs, combined datasets; MRDEG, mitophagy-related differentially expressed gene; AD, Alzheimer’s disease; DCA, decision curve analysis.

To determine the diagnostic value of the 53 identified MRDEGs in the AD dataset, a diagnostic model of the MRDEGs was constructed by LASSO regression analysis (Figure 5A) and visualized through a LASSO variable trajectory diagram (Figure 5B). The LASSO diagnostic model comprised 17 MRDEGs (Supplementary Table S4), and the expression levels of these genes in the different groups of the LASSO diagnostic model are illustrated by a forest plot (Figure 5C).

In addition, the expression levels of these 17 MRDEGs were used for uni- and multivariate Cox regression analyses, and a Cox regression model was constructed. The prognostic ability of the Cox regression model was evaluated based on a generated nomogram (Figure 5D). The calibration curve was drawn, and the predictive power of the model for the actual results was evaluated based on the fitting of the actual probability. The probability predicted by the model under different conditions is presented in Figure 5E. Finally, the clinical utility of the Cox regression model was evaluated by decision curve analysis (Figure 5F). The range was determined in which the line of the model remained stable and higher than “All positive” and “All negative”; the larger this range, the higher the net benefit, and the better the model effect.

### 3.4. Construction of the PPI network and screening of the hub genes

Initially, a PPI analysis was carried out, and the PPI network of the 53 MRDEGs was constructed using the STRING database. Interactions were visualized using the Cytoscape software (Supplementary Figure S1A). Among the 53 MRDEGs, 36 were related (Supplementary Table S4), and the scores provided by the STRING database were calculated by applying five algorithms of the CytoHubba plug-in. Then, the MRDEGs were arranged according to their scores. The five algorithms were maximum neighborhood component (Supplementary Figure S1B), degree (Supplementary Figure S1C), maximal clique centrality (Supplementary Figure S1D), closeness (Supplementary Figure S1E), and edge percolated component (Supplementary Figure S1F). The genes identified by the five algorithms were retrieved, and the Venn diagram was drawn to obtain the MRHGs (Supplementary Figure S1G). The six hub genes were *CD44*, *SUCLA2*, *DLAT*, *ITGAX*, *PPARG*, and *MYC*.

### 3.5. Construction of TF-mRNA and mRNA-miRNA regulatory networks

TFs associated with the MRHGs were obtained from the ChIPBase database, and the mRNA-TF regulatory network was constructed and visualized using the Cytoscape software (Supplementary Figure S2A). This network contained 6 MRHGs and 59 TFs. Likewise, the miRNAs related to the MRHGs were retrieved from the StarBase database, and the mRNA-miRNA regulatory network was constructed and visualized using Cytoscape (Supplementary Figure S2B). This network contained 6 MRHGs and 61 miRNAs.

### 3.6. Function enrichment (GO) analysis, pathway enrichment (KEGG) analysis of MRHGs, and GSEA of the AD dataset

Based on GO and KEGG enrichment analyses, the relationships among BPs, MFs, CCs, and biological pathways of the six MRDEGs discussed in section 3.5 and AD were further explored. The six MRHGs were applied to GO and KEGG gene function enrichment analysis (Tables 1, 2). The six MRHGs were mainly enriched in the regulation of BPs such as cysteine-type endopeptidase activity involved in apoptosis and negative regulation of fibroblast proliferation, CCs such as secretory granule membrane, tricarboxylic acid cycle enzyme complex, and lamellipodium membrane, and MFs such as E-box binding, repressing TF binding, and activating TF binding. Simultaneously, the MRHGs were also enriched in the tricarboxylic acid cycle, thyroid cancer, and carbon metabolism pathways, among others. The results of these analyses were visualized as a histogram (Supplementary Figure S3A), and GO (Supplementary Figures S3B–D) and KEGG (Supplementary Figure S3E) network maps were drawn. A connecting line indicates a molecule and the annotation of the corresponding entry. The larger the node, the more molecules the entry contains. Finally, GO and KEGG enrichment analyses of the combined logFC were performed for the six MRDEGs (Supplementary Figures S3F,G). Based on the enrichment analysis, the z-score corresponding to each entry was calculated using the molecular logFC. The results of the GO analysis visualized by a circle diagram (Supplementary Figure S3F) and those of the KEGG analysis visualized by a chord diagram (Supplementary Figure S3G) showed that cysteine-type endopeptidase activity involved in apoptosis may be the most important positive regulatory pathway, whereas the tricarboxylic acid cycle enzyme complex pathway may be the most influential negative regulatory pathway. The connecting line between the left and right parts shows the molecules included in the KEGG pathway entry.

**Table 1:** Results of GO enrichment analysis in AD.

Ontology	ID	Description	Gene ratio	Bg ratio	*p* value	*p*. adjust	*q* value
BP	GO:0043281	Regulation of cysteine-type endopeptidase activity involved in apoptotic process	3/6	215/18670	2.94e-05	0.004	0.002
BP	GO:0048147	Negative regulation of fibroblast proliferation	2/6	30/18670	3.73e-05	0.004	0.002
BP	GO:2000116	Regulation of cysteine-type endopeptidase activity	3/6	239/18670	4.03e-05	0.004	0.002
BP	GO:0006099	Tricarboxylic acid cycle	2/6	34/18670	4.81e-05	0.004	0.002
BP	GO:0006101	Citrate metabolic process	2/6	35/18670	5.10e-05	0.004	0.002
CC	GO:0030667	Secretory granule membrane	2/6	298/19717	0.003	0.050	0.031
CC	GO:0045239	Tricarboxylic acid cycle enzyme complex	1/6	14/19717	0.004	0.050	0.031
CC	GO:0031258	Lamellipodium membrane	1/6	22/19717	0.007	0.050	0.031
CC	GO:0005759	Mitochondrial matrix	2/6	469/19717	0.008	0.050	0.031
CC	GO:0008305	Integrin complex	1/6	31/19717	0.009	0.050	0.031
MF	GO:0070888	E-box binding	2/6	50/17697	1.17e-04	0.006	0.002
MF	GO:0070491	Repressing transcription factor binding	2/6	71/17697	2.36e-04	0.006	0.002
MF	GO:0033613	Activating transcription factor binding	2/6	85/17697	3.38e-04	0.006	0.002
MF	GO:0031406	Carboxylic acid binding	2/6	193/17697	0.002	0.020	0.005
MF	GO:0043177	Organic acid binding	2/6	205/17697	0.002	0.020	0.005

GO, Gene Ontology; BP, Biological Process; CC, Cellular Component; MF, Molecular Function, AD, Alzheimer’s Disease.

**Table 2:** Results of KEGG enrichment analysis in AD.

Ontology	ID	Description	Gene ratio	Bg ratio	*p* value	*p*. adjust	*q* value
KEGG	hsa00020	Citrate cycle (TCA cycle)	2/6	30/8076	1.98e-04	0.007	0.006
KEGG	hsa05216	Thyroid cancer	2/6	37/8076	3.03e-04	0.007	0.006
KEGG	hsa01200	Carbon metabolism	2/6	118/8076	0.003	0.050	0.039
KEGG	hsa05202	Transcriptional misregulation in cancer	2/6	192/8076	0.008	0.073	0.057
KEGG	hsa05169	Epstein–Barr virus infection	2/6	202/8076	0.009	0.073	0.057
KEGG	hsa05205	Proteoglycans in cancer	2/6	205/8076	0.009	0.073	0.057

KEGG, Kyoto Encyclopedia of Genes and Genomes; AD, Alzheimer’s Disease.

**Figure 6:**
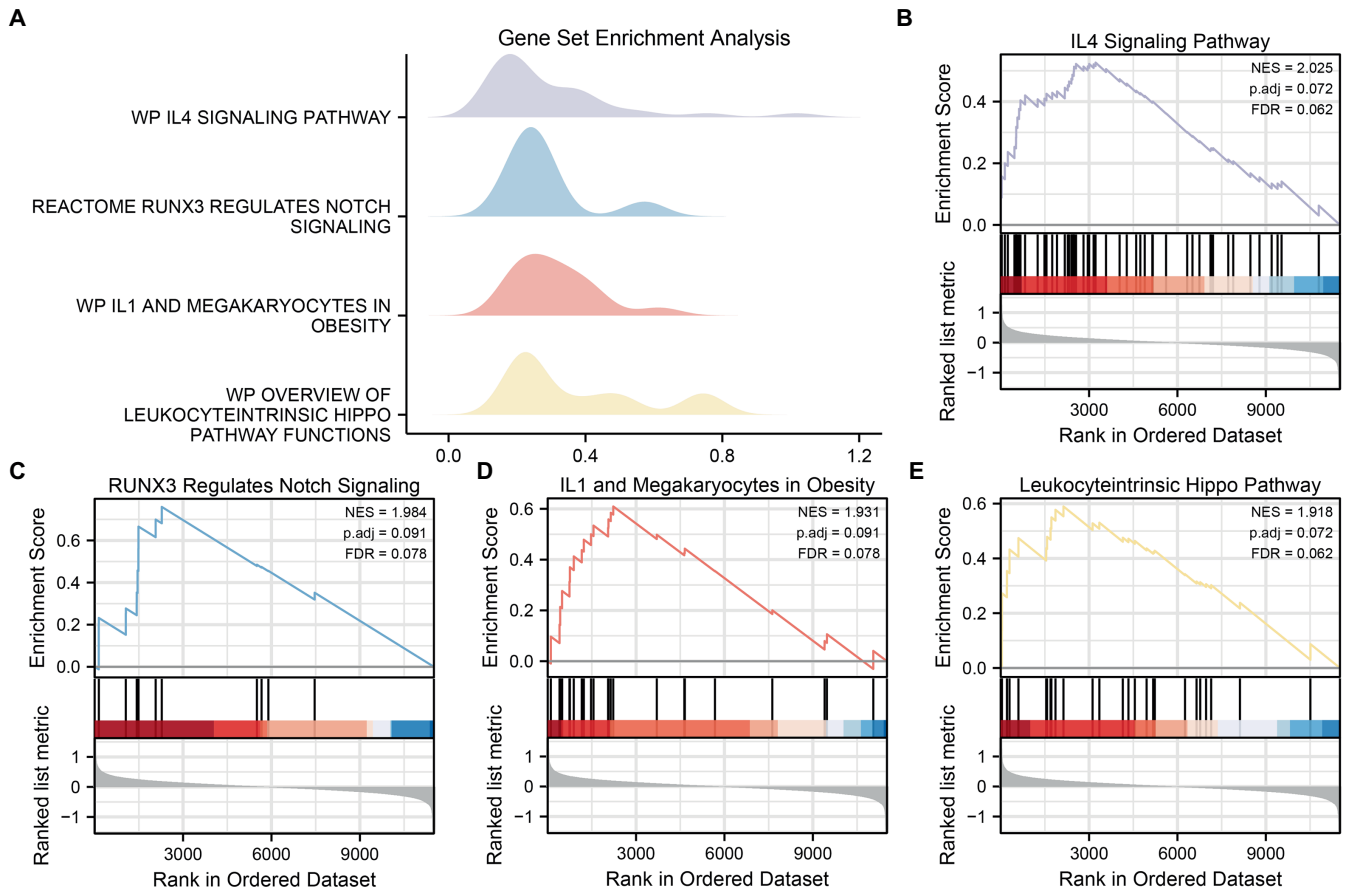
GSEA for CDs. **(A)** Mountain map of four biological functions in GSEA for CDs. **(B–E)** AD significantly affecting IL4 Signaling Pathway **(B)**, RUNX3 Regulations Notch Signaling **(C)**, IL1 and Megakaryocells in Obesity **(D)** and Overview of Leukocytric Hippo Pathway Functions **(E)** showed by GSEA. GSEA, gene set enrichment analysis; CDs, combined datasets; AD, Alzheimer’s disease.

GSEA was used to determine the effects of the DEG expression levels in the AD datasets, specifically the relationships between DEG expression in the CDs and the BPs involved, the CCs affected, and the MFs exerted. As shown in Table 3, DEGs in the CDs significantly affected biologically related functions and signaling pathways (Figures 6A–E) such as IL-4 Signaling Pathway (Figure 6B), RUNX3 Regulates Notch Signaling (Figure 6C), IL-1 and Megakaryocytes in Obesity Pathway (Figure 6D), and Overview of Leukocyteintrinsic Hippo Pathway (Figure 6E).

**Table 3:** Results of combined datasets GSEA in AD.

ID	Set size	Enrichment score	NES	*p* value	*p*. adjust	*q* values
REACTOME_INTERLEUKIN_10_SIGNALING	41	0.6888	2.5057	0.0021	0.0724	0.0621
PID_FRA_PATHWAY	34	0.6655	2.3017	0.0021	0.0724	0.0621
WP_TYROBP_CAUSAL_NETWORK	50	0.5603	2.1308	0.0021	0.0724	0.0621
PID_AMB2_NEUTROPHILS_PATHWAY	38	0.5922	2.1238	0.0021	0.0724	0.0621
WP_COMPLEMENT_AND_COAGULATION_CASCADES	54	0.5358	2.0661	0.0021	0.0724	0.0621
WP_IL4_SIGNALING_PATHWAY	53	0.5266	2.0246	0.0021	0.0724	0.0621
KEGG_CYTOKINE_CYTOKINE_RECEPTOR_INTERACTION	221	0.4197	2.0204	0.0021	0.0724	0.0621
PID_P73PATHWAY	74	0.4962	2.0181	0.0021	0.0724	0.0621
REACTOME_RUNX3_ MEDIATED_NOTCH_SIGNALING	12	0.7595	1.9835	0.0040	0.0905	0.0776
KEGG_HEMATOPOIETIC_CELL_LINEAGE	77	0.4812	1.9740	0.0021	0.0724	0.0621
REACTOME_INITIAL_TRIGGERING_OF_COMPLEMENT	20	0.6463	1.9598	0.0041	0.0905	0.0776
REACTOME_TRAF6_MEDIATED_IRF7_ACTIVATION	25	0.6132	1.9565	0.0041	0.0905	0.0776
WP_INTERACTIONS_BETWEEN_IMMUNE_CELLS_AND_MICRORNAS_IN_TUMOR_MICROENVIRONMENT	26	0.6009	1.9514	0.0020	0.0724	0.0621
REACTOME_INTERLEUKIN_4_AND_INTERLEUKIN_13_SIGNALING	103	0.4556	1.9487	0.0021	0.0724	0.0621
REACTOME_SYNTHESIS_OF_LEUKOTRIENES_LT_AND_EOXINS_EX_	16	0.6814	1.9459	0.0041	0.0905	0.0776
REACTOME_YAP1_AND_WWTR1_TAZ_STIMULATED_GENE_EXPRESSION	13	0.7304	1.9355	0.0062	0.1102	0.0945
REACTOME_NOTCH4_INTRACELLULAR_DOMAIN_REGULATES_TRANSCRIPTION	17	0.6647	1.9314	0.0042	0.0905	0.0776
WP_IL1_AND_MEGAKARYOCYTES_IN_OBESITY	24	0.6094	1.9314	0.0040	0.0905	0.0776
REACTOME_REGULATION_OF_GENE_EXPRESSION_IN_LATE_STAGE_BRANCHING_MORPHOGENESIS_ PANCREATIC_BUD_PRECURSOR_CELLS	13	0.7237	1.9179	0.0062	0.1102	0.0945
WP_OVERVIEW_OF_LEUKOCYTEINTRINSIC_HIPPO_PATHWAY_FUNCTIONS	27	0.5898	1.9178	0.0020	0.0724	0.0621

GSEA, Gene Set Enrichment Analysis; AD, Alzheimer’s Disease; NES, Normalized Enrichment score.

### 3.7. Construction of AD subtypes

**Figure 7:**
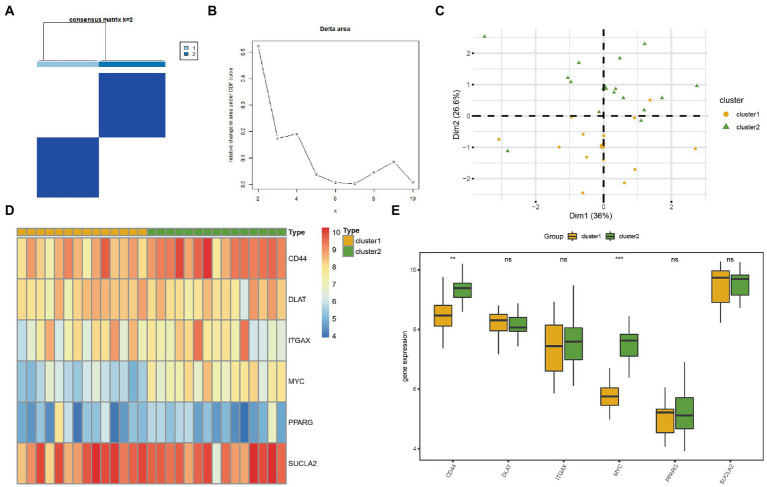
Consensus clustering analysis for hub genes. **(A)** Consistency clustering results of AD. **(B)** Delta diagram of different classifications by consistency clustering analysis. **(C)** PCA diagram of two AD subtypes. **(D)** Complex numerical heat map of MRHGs in two AD subtypes. **(E)** Histogram of grouping comparison of MRHGs in two AD subtypes. Orange is Cluster 1, and green is Cluster 2. **p value < 0.01, ***p value < 0.001. AD, Alzheimer’s disease; PCA, principal component analysis; MRHG, mitophagy-related hub gene.

To explore the differences in MRG expression in the AD subgroup of the CDs, the R package *ConsensusClusterPlus* was used for consistent clustering analyses to identify different AD subtypes based on the six MRHGs. Two AD subtypes were finally identified: Cluster 1 containing 14 samples represented subtype A, whereas Cluster 2 containing 15 samples represented subtype B (Figures 7A,B). The PCA showed significant differences between these two subtypes (Figure 7C). A heatmap was drawn using the *pheatmap* package to visualize the differences in MRHG expression between the two AD subtypes (Figure 7D).

To further verify the expression differences of the six MRHGs in the AD datasets, the correlation between the expression levels of the six MRHGs in the CDs and the two AD subtypes and the results of the difference analysis were shown in a group comparison histogram (Figure 7E). The differential analysis results of the CDs showed that the two MRHGs were statistically significant (*p* < 0.05): the expression of MYC was statistically significant among different subtypes of AD (*p* < 0.001); the expression of CD44 was highly statistically significant between different subtypes of AD (*p* < 0.01). In addition, the expression levels of DLAT, ITGAX, PPARG and SUCLA2 were not statistically significant between AD subtypes (*p* ≥ 0.05).

### 3.8. Analysis of immune cell infiltration between the two AD subtypes

To explore the differences in immune cell infiltration between the identified AD subtypes, CIBERSORT and ssGSEA algorithms were used to analyze for all samples the abundance of infiltrating immune cells in the two AD subtypes.

Based on the results of the CIBERSORT analysis, a histogram of the proportion of immune cells in the AD samples was drawn (Supplementary Figure S4A). Next, the correlations of immune cell infiltration abundance in leukocyte gene signature matrix (LM22) in AD subtype A (Supplementary Figure S4B) and subtype B (Supplementary Figure S4C) were demonstrated by plotting correlation heatmaps. The results showed that the correlation between activated mast cells and regulatory T cells was the highest in subtype A (cor value = 0.90). By contrast, the correlation between M0 macrophages and neutrophils was the highest in subtype B (cor value = 0.84). In addition, the correlation between the abundance of LM22 immune cell infiltration and the expression of the six identified MRHGs in the samples of patients was analyzed by plotting the correlation heatmap of the two subtypes (Supplementary Figures S4D,E). The results showed that in subtype A, *PPARG* expression was significantly positively correlated with follicular helper T cell levels, and *DLAT* expression was significantly negatively correlated with the abundance of activated dendritic cells. In subtype B, *DLAT* expression was significantly negatively correlated with γδ T cell levels.

Similarly, immune cell infiltration was analyzed using ssGSEA. The correlation between the abundance of the 28 types of infiltrating immune cells in subtype A (Supplementary Figure S5A) and subtype B (Supplementary Figure S5B) of AD was demonstrated by plotting the correlation heatmap. The results showed that myeloid-derived suppressor cells (MDSCs) had the highest correlation with neutrophils, mast cells, and central memory CD8+ T cells (cor value = 0.89, 0.77, and 0.82, respectively) in subtype A. In subtype B, the correlation between MDSCs and activated dendritic cells was the highest (cor value = 0.78). Moreover, the correlation between the abundance of these 28 immune cell types and the expression of the six MRHGs in the samples of patients was analyzed by plotting the correlation heatmaps for the two AD subtypes (Supplementary Figures S5C,D). The results showed that in subtype A, *SUCLA2* expression was significantly positively correlated with the levels of effector memory CD4+ T cells and immature dendritic cells, whereas *DLAT* expression was significantly negatively correlated with the level of activated B cells. In subtype B, *SUCLA2* expression was significantly negatively correlated with activated B cell levels.

### 3.9. Dataset validation and ROC analysis

**Figure 8:**
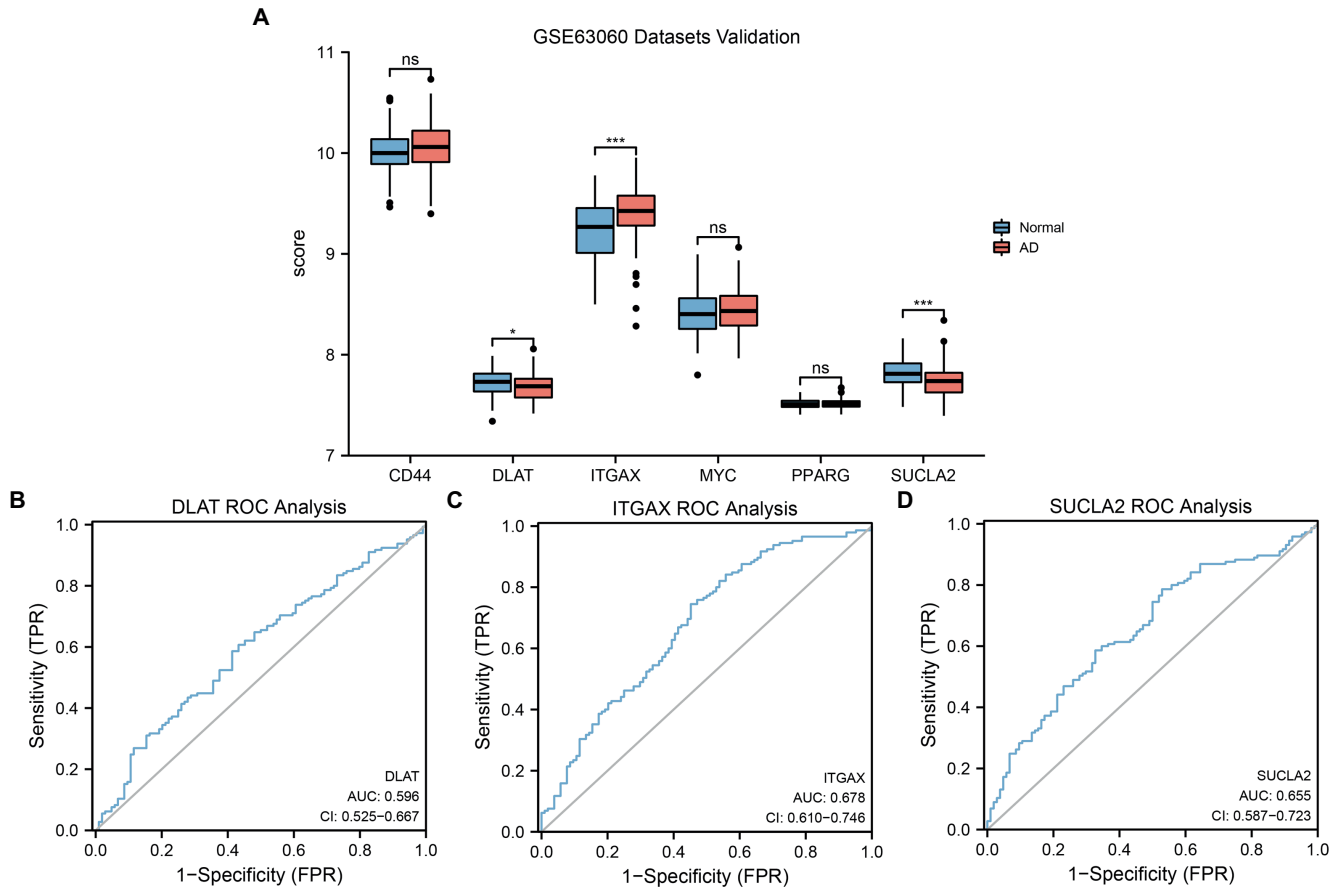
GSE63060 datasets validation and ROC analysis. **(A)** Comparison histogram of the results of differential expression analysis of MRHGs in the dataset GSE63060. **(B–D)** ROC curve of the MRHGs *DLAT* **(B)**, *ITGAX* **(C)** and *SUCLA2* **(D)** in dataset GSE63060. ns, *p* value ≥ 0.05, *p value < 0.05, ***p value < 0.001. AUC at 0.5–0.7 has low accuracy. ROC, receiver operating characteristic; MRHG, mitophagy-related hub gene.

To further verify differences in MRHG expression in the AD dataset, the results of the differential expression analysis comparing the levels of the six identified MRHGs between the AD and control groups of the GSE63060 dataset were displayed in a group comparison histogram. The differential expression analysis results (Figure 8A) showed that three MRHGs significantly differed between the two groups. Among them, the expression of *ITGAX* and *SUCLA2* in the AD and control groups of the GSE63060 dataset was markedly significantly different (*p* < 0.001), and the expression of *DLAT* was significantly different (*p* < 0.05). The expression levels of the other MRHGs (*CD44*, *MYC*, and *PPARG*) were not significantly different between groups. The ROC curves suggested a low accuracy for *DLAT* (AUC = 0.596, Figure 8B), *ITGAX* (AUC = 0.678, Figure 8C), and *SUCLA2* (AUC = 0.655, Figure 8D) to distinguish AD from control samples in the dataset GSE63060.

## 4. Discussion

AD is one of the main causes of dementia and death in the elderly, seriously endangering human health, and its course is usually 6–10 years. With the intensification of the global aging society, its incidence rate and prevalence rate are increasing. In March 2019, the Alzheimer’s Association of the United States released the impact of AD on public health in the United States. It is expected that by the middle of this century, the number of Americans aged 65 and over suffering from AD may increase to 13.8 million. However, up to now, there is a lack of ideal diagnostic indicators and effective prevention and treatment measures for AD. Therefore, it is of great social significance to strengthen the research on the pathogenesis of AD. Hence, it is crucial for this study to broaden its horizons to search for key molecules that may play a role in the pathogenesis of AD.

Mitophagy as a selective degradation process, is critical to keeping mitochondria healthy, producing ATP, and maintaining neuronal activity and survival by removing impaired mitochondria. MRGs have been previously reported as prognostic or diagnostic markers for various tumors, including pancreatic cancer (Zhuo et al., 2022), breast cancer (Zhao et al., 2022), and liver cancer (Chen et al., 2021; Xu et al., 2022), whereas only a limited number of studies have examined the usefulness of them as AD biomarkers. Pakpian et al. (2020) recently reported that alterations in mitochondrial dynamic-related genes in the peripheral blood may be useful for diagnosing AD. However, MRGs have not yet been evaluated for their diagnostic performance in AD and their role is worth further exploring.

In our study, we investigated the role of MRGs in the diagnosis of AD. Not only did we identify six MRHGs (*CD44*, *SUCLA2*, *DLAT*, *ITGAX*, *PPARG*, and *MYC*) as AD biomarkers, but we also used these MRHGs to create a diagnostic model. Moreover, a validation analysis conducted both internally and externally revealed that this model is effective in discriminating patients with AD from controls. Furthermore, we analyzed the relationships between MRHGs and immune cell infiltration in AD utilizing CIBERSORT and ssGSEA algorithms.

Some diagnostic biomarker signatures have been reported in previous studies. For example, Shigemizu et al. (2020) analyzed the blood samples of cognitively normal adults and patients with AD by RNA sequencing and detected DEGs. A model constructed by the proportion of neutrophils and the most important central genes (*EEF2* and *RPL7*) achieved an AUC of 0.878 in the validation cohort. Based on the results of its application to a prospective cohort, the model achieved an accuracy of 0.727, identifying blood-based biomarkers as early indicators of AD. Using the GEO database, researchers have identified in recent years numerous hub genes that are differentially expressed in AD and control brain samples and have further determined many possible diagnostic biomarkers of AD using the ROC prediction model. Tian et al. (2022) identified three hub genes (*MAFF*, *ADCYAP1*, and *ZFP36L1*; AUC = 0.850) and verified their expression in the AD brain (AUC = 0.935). Wu et al. (2021) found 10 hub genes, namely *SERPINE1*, *ZBTB16*, *CD44*, *BCL6*, *HMOX1*, *SLC11A1*, *CEACAM8*, *ITGA5*, *SOCS3*, and *IL4R*, all of which have good diagnostic value (AUC > 0.75). Liu et al. (2021) identified seven genes, including *ABCA2*, *CREBRF*, *CD72*, *CETN2*, *KCNG1*, *NDUFA2*, and *RPL36AL* (AUCs were 0.845 and 0.839 in the test and validation sets, respectively), as hub genes and confirmed them by reverse transcription polymerase chain reaction. Our team (Zhao et al., 2022) found that *AGAP3* is an important hub gene (AUCs in the three studied datasets were 0.878, 0.727, and 0.635), which may be a diagnostic biomarker related to immunity in AD. Among the six MRHGs identified in the current study, the expression levels of *CD44*, *SUCLA2*, *DLAT*, and *PPARG* in the CDs showed a medium correlation with the study groups, whereas *ITGAX* and *MYC* showed a low correlation. Our findings suggest that a combination of a few biomarkers performs fairly well as a diagnostic tool.

Of the six identified MRGs, *CD44*, *ITGAX*, and *PPARG* are clearly correlated with AD according to previous reports (Butovsky et al., 2006; Moreno-Rodriguez et al., 2020; Bottero et al., 2021). The CD44 protein encoded by the *CD44* gene is a cell surface glycoprotein and a receptor for hyaluronic acid. CD44 is involved in cell–cell interaction, as well as cell adhesion and migration (Wang et al., 2018; Moreno-Rodriguez et al., 2020). It is described as a multifaceted molecule involved in several biological and pathological processes. Western blot analyses revealed that CD44 levels of the frontal cortex were increased in sporadic AD and associated with disease progression (Moreno-Rodriguez et al., 2020). This is consistent with our prediction results to some extent. The gene *ITGAX* encodes an integrin α X-chain protein. Integrins are heterodimers composed of α and β chains to integrate membrane proteins, forming αXβ2 integrins (Golinski et al., 2020). *ITGAX* is considered the main driving factor of atherosclerosis (Williams et al., 2020). Using single-cell transcriptome analysis of the brain of AD mice, a recent study on the transcriptional characteristics of plaque-associated microglia found a two-step transition from homeostasis to pathologically related phenotypes, with *Trem2*, to which *Itgax* is related, as the main phenotypic regulator (Mancuso et al., 2019). Ramesha et al. (2021) reported that the inoculation of T cell-based Gramer acetate vaccine against AD-induced dendritic microglia to express *Itgax* and found that the plaque formation and cognitive ability of APP/PS1 mice were reduced (Ramesha et al., 2021). These discoveries are consistent with our predicted changes in the expression of *ITGAX* in AD. The PPAR γ protein encoded by the gene *PPARG*, a member of the peroxisome proliferator-activated receptor subfamily, is a regulator of lipid metabolism and inflammatory response mediators; it may regulate AD switch genes as a TF. It is involved in the pathology of many disorders, such as obesity, atherosclerosis, and AD (Bottero et al., 2021). Activation of platelets and phospholipase D are regarded as its key signal components (Bottero et al., 2021). Like *APOE*, *PPARG* is an important risk gene. CG or GG, which are participant genotypes of rs1805192 in *PPARG*, confer the highest risk for AD (Wang et al., 2017). These research data above support our prediction results. Overall, these findings may account for the distinct role of these genes in AD.

It has been suggested that the three genes *CD44*, *ITGAX*, and *PPARG* participate in AD pathogenesis mainly through neuroinflammation and immune pathways, making them promising therapeutic targets. Previous studies have shown that *CD44* may be highly involved in biological processes and pathways related to immune inflammatory response, apoptosis, and mitogen-activated protein kinase pathways in AD (Shim et al., 2022; Xu et al., 2022). A systematic review found that *CD44* is related to the complexity of reactive astrocytosis in AD (Viejo et al., 2022). As a microglia-related gene, *ITGAX* was found to be differentially expressed in AD and possibly involved in neuroinflammation, oxidative stress, and Aβ autophagy and transport (Li and de Muynck, 2021; Wu et al., 2021). The *PPARG* gene may increase the incidence of AD in patients with psoriasis by activating a positive feedback loop leading to excessive inflammation and metabolic disorder (Liu et al., 2022).

However, the relationship of *SUCLA2*, *DLAT*, and *MYC* expression with AD has not been previously reported. The *SUCLA2* gene encodes ATP-specific Succinyl-CoA synthetase (SCS) β subunits, which dimerize with SCS α subunits to form SCS-A, a heterodimeric mitochondrial matrix enzyme, which is an important component of the tricarboxylic acid cycle. By hydrolyzing ATP, SCS-A forms succinic acid and succinyl-CoA. Mutations of *SUCLA2* are one of the causes of myopathic mitochondrial DNA deletion syndrome (Viscomi and Zeviani, 2017). This is somewhat different from our predicted results, and further prospective studies are needed to determine the diagnostic accuracy of *SUCLA2* for AD. The gene *DLAT* encodes the E2 component of the multi-enzyme pyruvate dehydrogenase complex, which is a lipoylated core protein (Carrico et al., 2018). The protein, which is also an antigen of anti-mitochondrial antibody, accepts the acetyl group formed by oxidative decarboxylation of pyruvate and transfers it to coenzyme A. It has been reported that DLAT is the key mediator of cell survival in chronic myeloid leukemia after tyrosine kinase inhibitor-mediated BCR-ABL1 inhibition (Bencomo-Alvarez et al., 2019). It has also been found that SIRT4 can hydrolyze the lipoamide cofactors derived from DLAT, leading to a decrease in pyruvate dehydrogenase activity (Xie et al., 2020). These’re not the same as our prediction, and the correlation between *DLAT* expression and AD needs to be thoroughly studied. The proto-oncogene *MYC* encodes a nuclear phosphoprotein that is crucial for the progression of the cell cycle, apoptosis, and transformation of cells (Casey et al., 2018). Its amplification is often observed in human tumors, and many drugs targeting the *MYC* pathway can be used for the treatment of tumors; the therapeutic effect might be related to the ability to restore the immune response (Casey et al., 2018). In addition, *MYC* expression is temporarily upregulated in spinal microglia as a TF after nerve injury to mediate early-phase proliferation of microglia, which is recognized as a hallmark of AD (Tan et al., 2022). The above indirectly reveals the possibility that *MYC* may participate in AD, but the diagnostic value of *MYC* in our research results needs to be further verified.

In this study, the differential expression analysis results of the CDs showed that the expression of *CD44* was highly statistically significant and that of *MYC* was statistically significant between different AD subtypes. In the validation dataset GSE63060, *ITGAX* and *SUCLA2* expression was markedly significantly different between the AD and control groups, whereas *DLAT* expression was significantly different between these two groups. The ROC curves of these three genes in the dataset GSE63060 showed that the expression levels of *DLAT*, *ITGAX*, and *SUCLA2* suggested a low correlation with study group. Thus, we will further explore the biological role of these genes in AD in the future.

In the last few decades, increasingly compelling evidence has emerged showing that AD is associated with immune system imbalance (Heneka et al., 2015; Lewcock et al., 2020). For example, compared with healthy controls, patients with AD have higher numbers of neutrophils, CD4+ T cells, and monocytes in the whole blood (Ferretti et al., 2016; Sommer et al., 2017; Unger et al., 2020). However, there remains a lack of clarity regarding the activation pattern of immune cells in AD. In the current study, an in-depth evaluation of AD immune cell infiltration was conducted using CIBERSORT and ssGSEA to further understand the role of immune responses in AD subtypes. The results of the CIBERSORT analysis showed that the correlation between activated mast cells and regulatory T cells was the highest in subtype A, whereas the correlation between M0 macrophages and neutrophils was the highest in subtype B. Similarly, the results of the ssGSEA showed that MDSCs had the highest correlation with neutrophils, mast cells, and central memory CD8+ T cells in subtype A, whereas the correlation between MDSCs and activated dendritic cells was the highest in subtype B.

In addition, our data mining results further confirmed that mitophagy and immunity may play key roles in the pathogenesis of AD. According to recent research, the cellular components of the immune system that may be modulated by mitophagy include natural killer cells, macrophages, dendritic cells, and T and B lymphocytes (Fang et al., 2019; Xu and Jia, 2021; Xie et al., 2022). Thus, we also analyzed the correlation between the six identified MRHGs (*CD44*, *SUCLA2*, *DLAT*, *ITGAX*, *PPARG*, and *MYC*) and infiltrating immune cells. Our results showed that *DLAT*, *PPARG*, and *SUCLA2* may be significantly correlated with distinct immune cell subsets indicative of different immune responses of AD subtypes. The correlation heatmap of the CIBERSORT analysis showed that *PPARG* expression was significantly positively correlated with follicular helper T cell levels in subtype A, whereas *DLAT* expression was significantly negatively correlated with the levels of activated dendritic cells in subtype A and with those of γδ T cells in subtype B. The correlation heatmap of the ssGSEA showed that *SUCLA2* expression was significantly positively correlated with the levels of effector memory CD4+ T cells and immature dendritic cells and significantly negatively correlated with the levels of activated B cells in subtype A, whereas *DLAT* expression was significantly negatively correlated with activated B cell levels in subtype B.

According to these findings, significant correlations exist between most MRGs and immune cells, which may indicate that mitophagy and immune responses interact in AD. This may further the understanding of the MRG-dependent immune status and microenvironment in AD. However, these assumptions require further studies to clarify the molecular mechanisms of the complex interaction between these genes and immune cells.

Clinically, AD can be divided into familial AD and sporadic AD according to genetic history and into early-onset AD and late-onset AD according to the age of onset. In this study, subtypes were only based on bioinformatics clusters according to the gene expression matrix. No other specific characteristics were taken into consideration, but correlations with clinical AD classifications may exist. In the future, we will aim to specify the degree of correlations and connections. Moreover, we will collect samples from AD patients in our hospital, record their clinical types, and determine whether clinical classifications are related to subtypes established by bioinformatics approaches.

The biomarkers identified in this study have several advantages. First, it is the first study to comprehensively explain the relationship between biomarkers and AD from the perspective of mitophagy compared with classic genetic biomarkers such as *APP* and *PSEN1*. Second, by combining three datasets, the sample number was sufficient, and the interbatch differences in the datasets were eliminated, avoiding data bias (see Figure 2). Finally, the validation using an external dataset has further consolidated the conclusions of this study.

Our research has some limitations. First, our research was conducted using secondary mining and analysis of previously published datasets. Second, the external validation was only performed on one dataset, which was relatively small, although the development set had sufficient whole-blood samples from patients with AD and healthy controls. Third, although the AUC of the model showed acceptable discrimination, the performance of the model requires improvement. Therefore, it is vital to guarantee a large sample size for independent research to verify and improve the clinical practicability. Finally, the mechanisms and relationships of MRGs are included in gene signatures, which needs further study.

## 5. Conclusion

We identified six MRHGs that may represent peripheral blood-derived diagnostic biomarkers and may participate in the pathological mechanisms of AD. Furthermore, a diagnostic model of AD based on MRGs was constructed utilizing LASSO and logistic regression, and it exhibited good diagnostic performance in internal and external validation. Moreover, CIBERSORT and ssGSEA were used to analyze the immune cell infiltration in patients with AD, and the correlation analysis showed that mitophagy might modulate the immune response of patients with AD. These findings expand our understanding of the role of MRGs in AD. Our gene signatures may, therefore, provide an accurate and reliable prediction method for the diagnosis of patients with AD.

## Data availability statement

The original contributions presented in the study are included in the article/Supplementary material, further inquiries can be directed to the corresponding author.

## Author contributions

KZ: study design, data acquisition, analysis, completed drawing, and writing. YiW, DZ, HZ, and JL: data acquisition and analysis, and editing of the manuscript. YuW: data analysis, interpretation, and critical revision of the manuscript. All authors read and approved the final manuscript.

## Funding

This work was supported by the research foundation of Affiliated People’s Hospital of Jiangsu University (Y2020003-S) and Project 333 of Jiangsu Province (BRA2019231).

## Conflict of interest

The authors declare that the research was conducted in the absence of any commercial or financial relationships that could be construed as a potential conflict of interest.

## Publisher’s note

All claims expressed in this article are solely those of the authors and do not necessarily represent those of their affiliated organizations, or those of the publisher, the editors and the reviewers. Any product that may be evaluated in this article, or claim that may be made by its manufacturer, is not guaranteed or endorsed by the publisher.

## Abbreviations

AD: Alzheimer’s disease
*APOE*: apolipoprotein E
AUROC: area under the receiver operating characteristics curve
Aβ: amyloid-β
BP: biological process
CC: cellular component
CDs: combined datasets
DEG: differentially expressed gene
*DLAT*: dihydrolipoamide S-acetyltransferase
GEO: Gene Expression Omnibus
GO: Gene Ontology
GSEA: gene set enrichment analysis
*ITGAX*: integrin subunit alpha X
KEGG: Kyoto Encyclopedia of Genes and Genomes
LASSO: least absolute shrinkage and selection operator
MDSC: myeloid-derived suppressor cell
MF: molecular function
MRDEG: mitophagy-related differentially expressed gene
MRG: mitophagy-related gene
MRHG: mitophagy-related hub gene
PCA: principal component analysis
*PPARG* (PPAR γ): peroxisome proliferator-activated receptor gamma
PPI: protein–protein interaction
ROC: receiver operating characteristic
ssGSEA: single-sample gene set enrichment analysis
*SUCLA2*: succinate-CoA ligase ADP-forming subunit β
TF: transcription factors
*TREM2*: triggering receptor expressed on myeloid cells 2
