# Supplementary figures and images for: Six mitophagy-related hub genes as peripheral blood biomarkers of Alzheimer’s disease and their immune cell infiltration correlation
Source: Front Neurosci. 2023 May 18;17:1125281. doi: 10.3389/fnins.2023.1125281 (PMC10232817; doi:10.3389/fnins.2023.1125281)

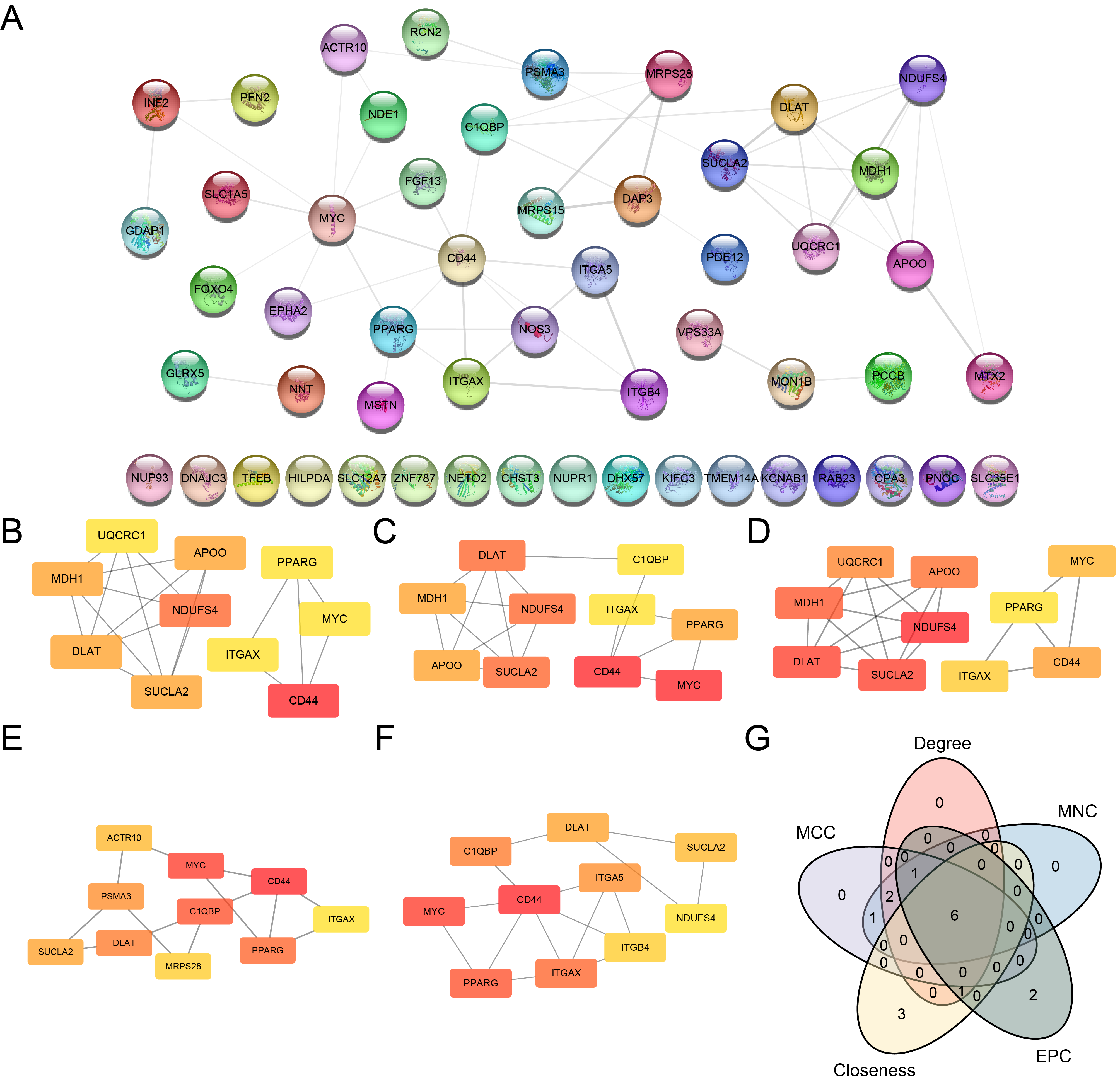

Supplement: Supplementary file 1 [file Image_1.TIF]

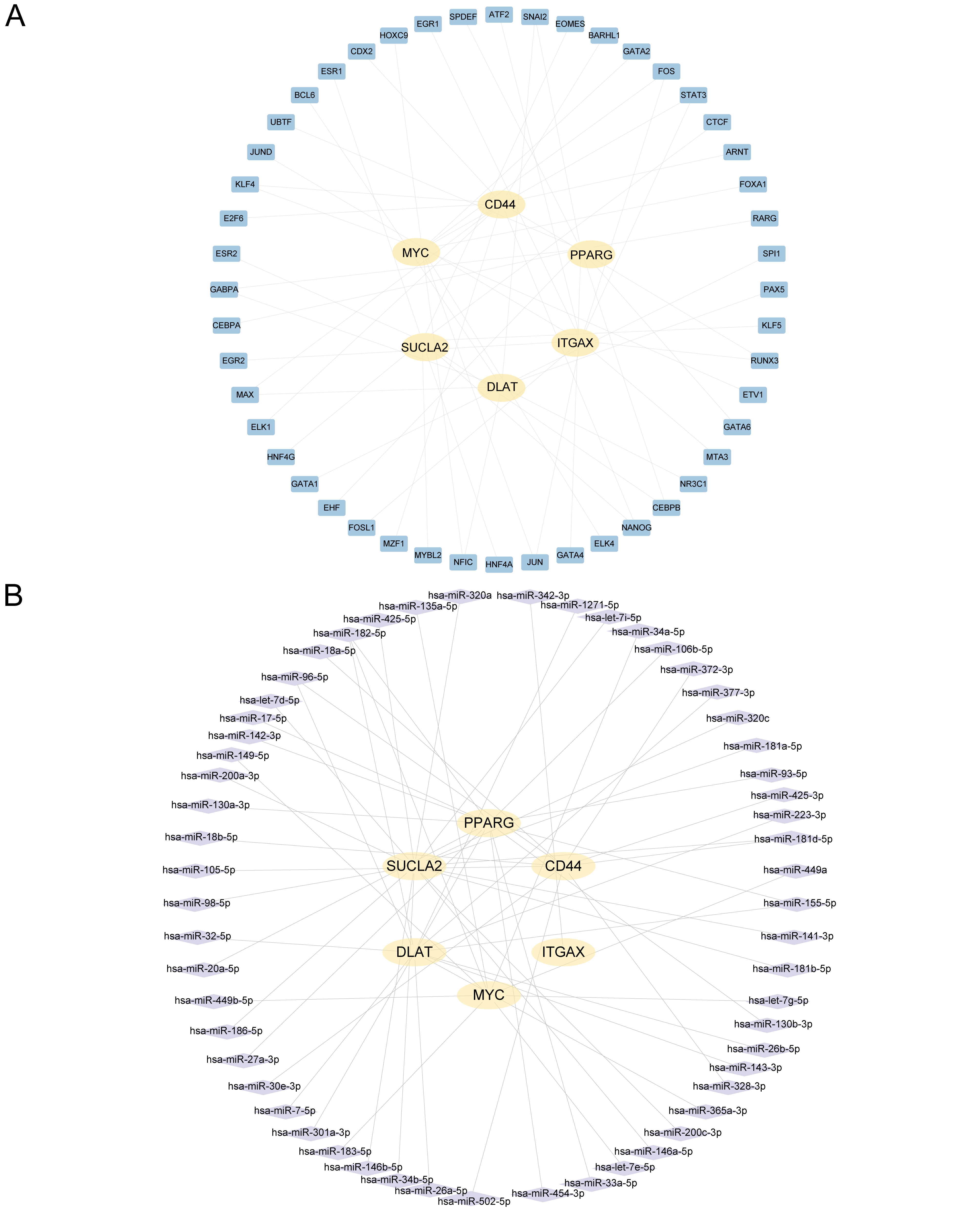

Supplement: Supplementary file 2 [file Image_2.TIF]

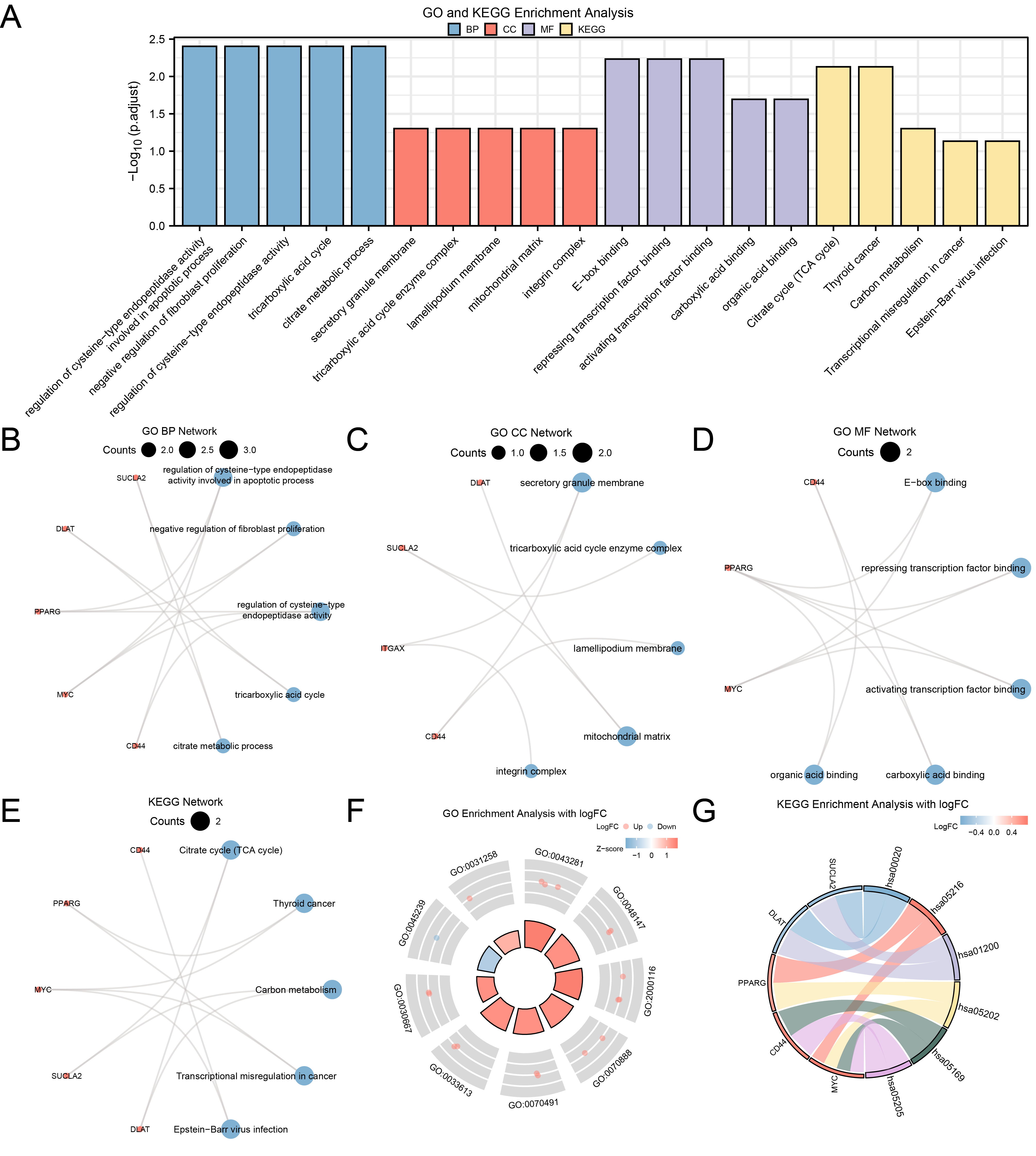

Supplement: Supplementary file 3 [file Image_3.TIF]

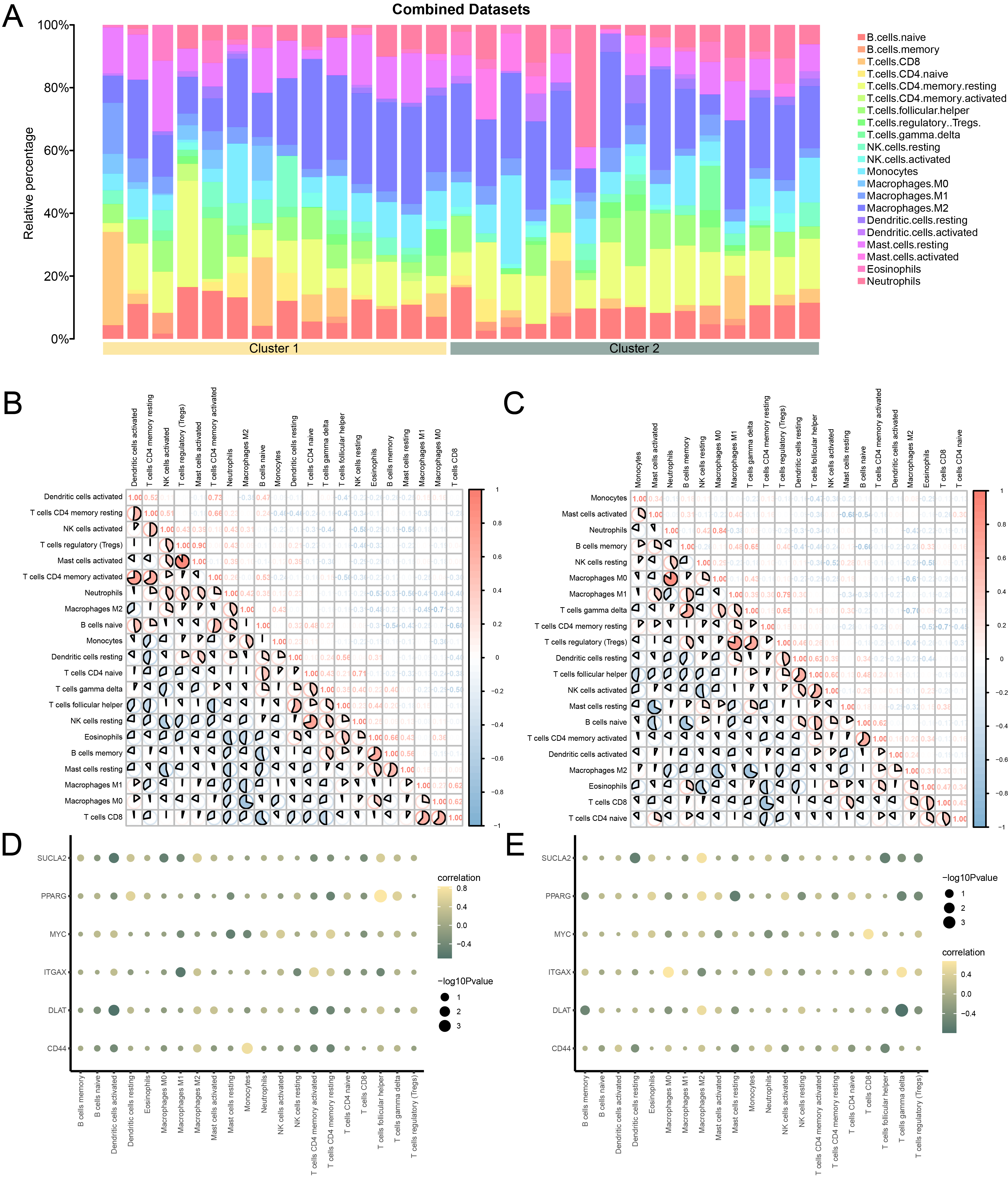

Supplement: Supplementary file 4 [file Image_4.TIF]

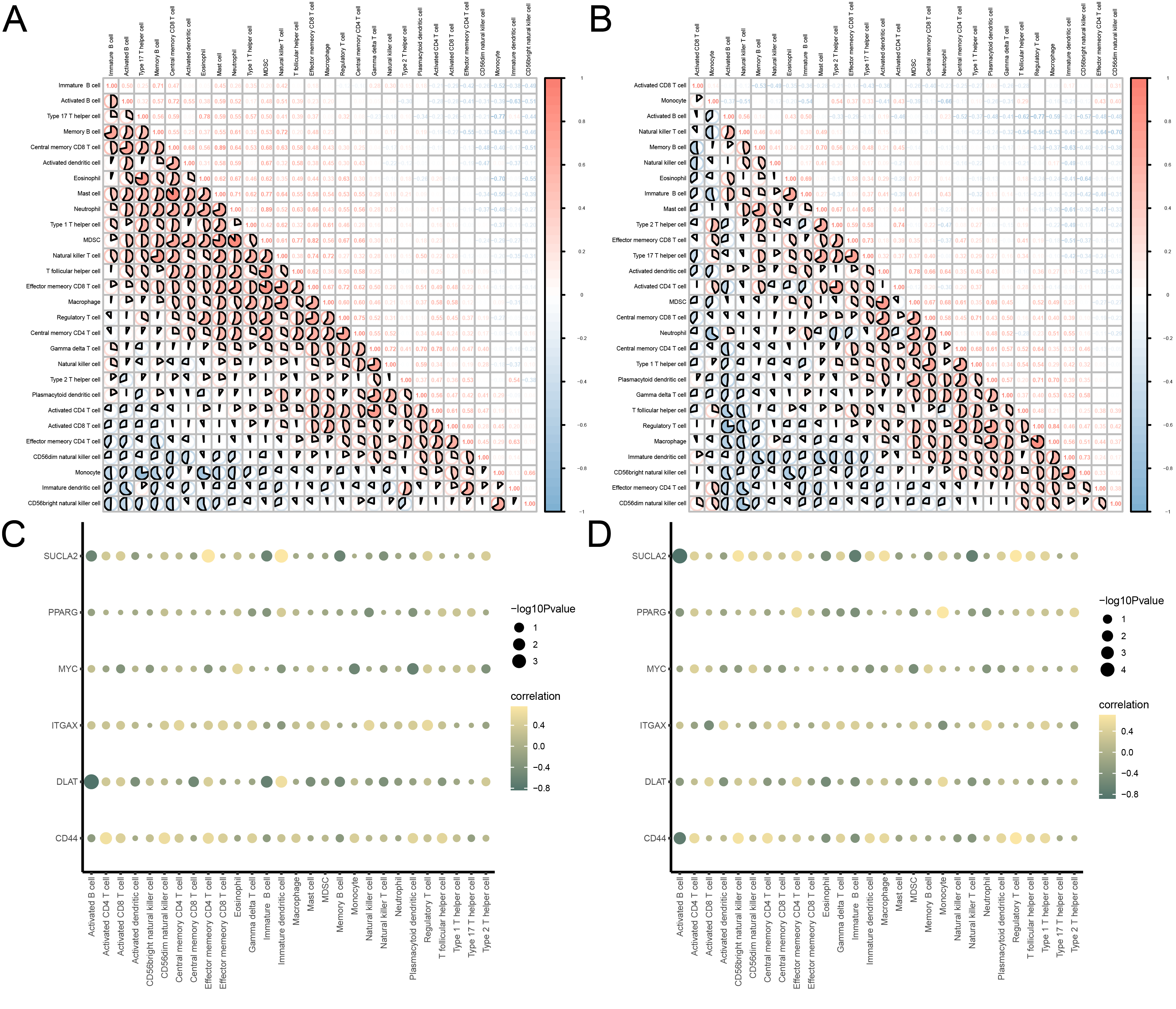

Supplement: Supplementary file 5 [file Image_5.TIF]
