# Supplementary material for: Six mitophagy-related hub genes as peripheral blood biomarkers of Alzheimer’s disease and their immune cell infiltration correlation
Source: Front Neurosci. 2023 May 18;17:1125281. doi: 10.3389/fnins.2023.1125281 (PMC10232817; doi:10.3389/fnins.2023.1125281)
Supplement: Supplementary file 6 [file Table_1.pdf]

Table S1. The information of two GEO Microarray Chip Information in AD

	**GSE110226**	**GSE1297**
Platform	GPL10379	GPL96
Species	Homo sapiens	Homo sapiens
Tissue	Choroid Plexus	Hippocampal CA1
Samples in AD group	7	22
Samples in Normal group	6	9
Reference	PMID: 29848382<br>PMID: 30541599	PMID: 14769913

GEO, Gene Expression Omnibus; AD, Alzheimer's Disease
