# Supplementary material for: Six mitophagy-related hub genes as peripheral blood biomarkers of Alzheimer’s disease and their immune cell infiltration correlation
Source: Front Neurosci. 2023 May 18;17:1125281. doi: 10.3389/fnins.2023.1125281 (PMC10232817; doi:10.3389/fnins.2023.1125281)
Supplement: Supplementary file 7 [file Table_2.pdf]

Table S2. Mitophagy-Related Genes

Gene Symbol	Description	Category	Gifts	GC Id	Relevance score
PRKN	Parkin RBR E3 Ubiquitin Protein Ligase	Protein Coding	37	GC06M161348	16.70443344
PINK1	PTEN Induced Kinase 1	Protein Coding	45	GC01P020634	15.61451721
MAP1LC3B	Microtubule Associated Protein 1 Light Chain 3 Beta	Protein Coding	40	GC16P087384	9.382333755
VDAC1	Voltage Dependent Anion Channel 1	Protein Coding	45	GC05M133975	8.422808647
FUNDC1	FUN14 Domain Containing 1	Protein Coding	33	GC0XM044523	7.76328373
MFN2	Mitofusin 2	Protein Coding	46	GC01P011980	7.512325764
SQSTM1	Sequestosome 1	Protein Coding	46	GC05P179806	6.747109413
MAP1LC3A	Microtubule Associated Protein 1 Light Chain 3 Alpha	Protein Coding	40	GC20P034546	6.161763191
ATG13	Autophagy Related 13	Protein Coding	36	GC11P046648	5.962795258
PHB2	Prohibitin 2	Protein Coding	41	GC12M006965	5.911291122
ULK1	Unc-51 Like Autophagy Activating Kinase 1	Protein Coding	42	GC12P131894	5.619565487
AMBRA1	Autophagy And Beclin 1 Regulator 1	Protein Coding	37	GC11M089343	5.539550304
UBC	Ubiquitin C	Protein Coding	41	GC12M124911	5.329469681
TOMM20	Translocase Of Outer Mitochondrial Membrane 20	Protein Coding	38	GC01M235109	5.164019585
USP30	Ubiquitin Specific Peptidase 30	Protein Coding	38	GC12P109027	5.061582565
CLEC16A	C-Type Lectin Domain Containing 16A	Protein Coding	36	GC16P010944	4.679720402
DNM1L	Dynamin 1 Like	Protein Coding	46	GC12P032679	4.608840942
RIMOC1	RAB7A Interacting MON1-CCZ1 Complex Subunit 1	Protein Coding	23	GC05P041991	4.494271755
UBA52	Ubiquitin A-52 Residue Ribosomal Protein Fusion Product 1	Protein Coding	40	GC19P018563	4.39316988
MFN1	Mitofusin 1	Protein Coding	41	GC03P179347	4.314269066
HUWE1	HECT, UBA And WWE Domain Containing E3 Ubiquitin Protein Ligase 1	Protein Coding	42	GC0XM053532	4.276308537
PGAM5	PGAM Family Member 5, Mitochondrial Serine/Threonine Protein Phosphatase	Protein Coding	34	GC12P132710	4.261062622
OPTN	Optineurin	Protein Coding	43	GC10P013099	4.163879395
SLC25A4	Solute Carrier Family 25 Member 4	Protein Coding	47	GC04P185143	4.157104015
RNF41	Ring Finger Protein 41	Protein Coding	38	GC12M056202	4.049372196
ATG7	Autophagy Related 7	Protein Coding	41	GC03P012474	3.942675114
UBB	Ubiquitin B	Protein Coding	41	GC17P016380	3.916631937
SLC25A5	Solute Carrier Family 25 Member 5	Protein Coding	42	GC0XP119468	3.863794804
VPS13D	Vacuolar Protein Sorting 13 Homolog D	Protein Coding	34	GC01P012236	3.833941936
TOMM40	Translocase Of Outer Mitochondrial Membrane 40	Protein Coding	40	GC19P044890	3.829195023
VPS13C	Vacuolar Protein Sorting 13 Homolog C	Protein Coding	33	GC15M061852	3.807225227
ATG4D	Autophagy Related 4D Cysteine Peptidase	Protein Coding	36	GC19P010543	3.710482359
TAFAZZIN	Tafazzin, Phospholipid-Lysophospholipid Transacylase	Protein Coding	32	GC0XP154413	3.703956604
ATG4B	Autophagy Related 4B Cysteine Peptidase	Protein Coding	40	GC02P241637	3.652068853
USP15	Ubiquitin Specific Peptidase 15	Protein Coding	44	GC12P062260	3.55750227
GABARAPL2	GABA Type A Receptor Associated Protein Like 2	Protein Coding	41	GC16P075566	3.545252085
BNIP3L	BCL2 Interacting Protein 3 Like	Protein Coding	40	GC08P026383	3.543636799
TOMM7	Translocase Of Outer Mitochondrial Membrane 7	Protein Coding	35	GC07M022816	3.541124821
ABCE1	ATP Binding Cassette Subfamily E Member 1	Protein Coding	38	GC04P145097	3.479506016
BECN1	Beclin 1	Protein Coding	44	GC17M042810	3.479400635
CERS1	Ceramide Synthase 1	Protein Coding	40	GC19M018868	3.430210114
ATG5	Autophagy Related 5	Protein Coding	42	GC06M106045	3.400434971
TIGAR	TP53 Induced Glycolysis Regulatory Phosphatase	Protein Coding	36	GC12P021012	3.268504381
RHOT1	Ras Homolog Family Member T1	Protein Coding	41	GC17P032142	3.256548166
LRBA	LPS Responsive Beige-Like Anchor Protein	Protein Coding	40	GC04M150264	3.248070955
ARFIP2	ADP Ribosylation Factor Interacting Protein 2	Protein Coding	38	GC11M006483	3.248070955
RPS27A	Ribosomal Protein S27a	Protein Coding	41	GC02P055231	3.245027304
TOMM22	Translocase Of Outer Mitochondrial Membrane 22	Protein Coding	34	GC22P038681	3.204845667
PHB1	Prohibitin 1	Protein Coding	36	GC17M049406	3.172647953
TOMM70	Translocase Of Outer Mitochondrial Membrane 70	Protein Coding	30	GC03M100364	3.156105995
FBXO7	F-Box Protein 7	Protein Coding	42	GC22P032474	3.144320011
BNIP3	BCL2 Interacting Protein 3	Protein Coding	40	GC10M131966	3.127365112
RAB7A	RAB7A, Member RAS Oncogene Family	Protein Coding	45	GC03P134197	3.120419741
MAP1LC3B2	Microtubule Associated Protein 1 Light Chain 3 Beta 2	Protein Coding	32	GC12P116559	2.965862751
MUL1	Mitochondrial E3 Ubiquitin Protein Ligase 1	Protein Coding	38	GC01M020499	2.858245373
GABARAP	GABA Type A Receptor-Associated Protein	Protein Coding	41	GC17M007240	2.857750177
CHUK	Component Of Inhibitor Of Nuclear Factor Kappa B Kinase Complex	Protein Coding	49	GC10M100188	2.761273146
ATG9A	Autophagy Related 9A	Protein Coding	38	GC02M219219	2.751641512
TP53	Tumor Protein P53	Protein Coding	50	GC17M007661	2.747530699
CISD1	CDGSH Iron Sulfur Domain 1	Protein Coding	37	GC10P058269	2.73961091
FKBP8	FKBP Prolyl Isomerase 8	Protein Coding	41	GC19M018503	2.718072176
CNOT4	CCR4-NOT Transcription Complex Subunit 4	Protein Coding	38	GC07M135361	2.660097599
CSNK2A1	Casein Kinase 2 Alpha 1	Protein Coding	48	GC20M000472	2.638748407
TBK1	TANK Binding Kinase 1	Protein Coding	47	GC12P064451	2.594376802
CSNK2A2	Casein Kinase 2 Alpha 2	Protein Coding	45	GC16M058157	2.556794405
MTX1	Metaxin 1	Protein Coding	38	GC01P155208	2.540253401
CALCOCO2	Calcium Binding And Coiled-Coil Domain 2	Protein Coding	38	GC17P055981	2.488806725
ATG4A	Autophagy Related 4A Cysteine Peptidase	Protein Coding	40	GC0XP108091	2.470188618
BCL2L13	BCL2 Like 13	Protein Coding	37	GC22P017628	2.450586796
SNCA	Synuclein Alpha	Protein Coding	48	GC04M089724	2.412575722
MON1A	MON1 Homolog A, Secretory Trafficking Associated	Protein Coding	36	GC03M051222	2.379864931
PELO	Pelota MRNA Surveillance And Ribosome Rescue Factor	Protein Coding	36	GC05P052787	2.295246363
VDAC2	Voltage Dependent Anion Channel 2	Protein Coding	42	GC10P075210	2.255251408
HDAC6	Histone Deacetylase 6	Protein Coding	50	GC0XP048801	2.222805738
LRRK2	Leucine Rich Repeat Kinase 2	Protein Coding	47	GC12P040196	2.214469671
PRKAA1	Protein Kinase AMP-Activated Catalytic Subunit Alpha 1	Protein Coding	45	GC05M040759	2.191478729
MTARC2	Mitochondrial Amidoxime Reducing Component 2	Protein Coding	28	GC01P220748	2.173646688
SPATA33	Spermatogenesis Associated 33	Protein Coding	30	GC16P091321	2.146743298
PTRH2	Peptidyl-TRNA Hydrolase 2	Protein Coding	41	GC17M059674	2.132793903
BCL2L1	BCL2 Like 1	Protein Coding	45	GC20M031664	2.131608486
OPA1	OPA1 Mitochondrial Dynamin Like GTPase	Protein Coding	44	GC03P193594	2.127315283
ATG14	Autophagy Related 14	Protein Coding	34	GC14M055366	2.122996807
TDRKH	Tudor And KH Domain Containing	Protein Coding	38	GC01M152176	2.109253645
GBA	Glucosylceramidase Beta	Protein Coding	48	GC01M155234	2.089705944
ATG12	Autophagy Related 12	Protein Coding	39	GC05M115828	2.073549032
UBE2L3	Ubiquitin Conjugating Enzyme E2 L3	Protein Coding	43	GC22P021549	2.067678928
MFF	Mitochondrial Fission Factor	Protein Coding	37	GC02P227325	2.065841436
PI4KB	Phosphatidylinositol 4-Kinase Beta	Protein Coding	42	GC01M151291	2.044233561
SIRT1	Sirtuin 1	Protein Coding	46	GC10P067884	2.044166088
WIPI1	WD Repeat Domain, Phosphoinositide Interacting 1	Protein Coding	37	GC17M068420	1.989925146
VCP	Valosin Containing Protein	Protein Coding	48	GC09M035056	1.974441528
NBR1	NBR1 Autophagy Cargo Receptor	Protein Coding	37	GC17P043170	1.974441528
SPATA18	Spermatogenesis Associated 18	Protein Coding	33	GC04P052051	1.909831285
HTRA2	HtrA Serine Peptidase 2	Protein Coding	44	GC02P074529	1.906463623
SESN2	Sestrin 2	Protein Coding	37	GC01P028270	1.905782938

FIS1	Fission, Mitochondrial 1	Protein Coding	37	GC07M101239	1.894189954
ATG4C	Autophagy Related 4C Cysteine Peptidase	Protein Coding	38	GC01P062784	1.884317636
SAMM50	SAMM50 Sorting And Assembly Machinery Component	Protein Coding	37	GC22P043955	1.883943439
IMMT	Inner Membrane Mitochondrial Protein	Protein Coding	40	GC02M086144	1.850885153
MAPK14	Mitogen-Activated Protein Kinase 14	Protein Coding	49	GC06P083841	1.846100092
FANCC	FA Complementation Group C	Protein Coding	46	GC09M095099	1.8381145
TBC1D5	TBC1 Domain Family Member 5	Protein Coding	36	GC03M017157	1.8381145
STX10	Syntaxin 10	Protein Coding	34	GC19M013144	1.8381145
MAP1LC3C	Microtubule Associated Protein 1 Light Chain 3 Gamma	Protein Coding	31	GC01M241995	1.815096378
STK4	Serine/Threonine Kinase 4	Protein Coding	48	GC20P044966	1.802430391
NLRP3	NLR Family Pyrin Domain Containing 3	Protein Coding	45	GC01P247415	1.800661683
MARCHF5	Membrane Associated Ring-CH-Type Finger 5	Protein Coding	30	GC10P092552	1.745763779
PLSCR1	Phospholipid Scramblase 1	Protein Coding	42	GC03M146515	1.730199695
BCL2	BCL2 Apoptosis Regulator	Protein Coding	48	GC18M063123	1.729902267
SRC	SRC Proto-Oncogene, Non-Receptor Tyrosine Kinase	Protein Coding	49	GC20P037344	1.70765996
CSNK2B	Casein Kinase 2 Beta	Protein Coding	46	GC06P083698	1.70765996
TOMM5	Translocase Of Outer Mitochondrial Membrane 5	Protein Coding	33	GC09M037582	1.70765996
MTERF3	Mitochondrial Transcription Termination Factor 3	Protein Coding	30	GC08M096240	1.70765996
TOMM6	Translocase Of Outer Mitochondrial Membrane 6	Protein Coding	29	GC06P041787	1.70765996
MIEF1	Mitochondrial Elongation Factor 1	Protein Coding	32	GC22P039510	1.703234434
BAG5	BAG Cochaperone 5	Protein Coding	40	GC14M103556	1.69826901
PEX13	Peroxisomal Biogenesis Factor 13	Protein Coding	40	GC02P061017	1.692638159
MTX2	Metaxin 2	Protein Coding	41	GC02P176269	1.649554133
HSPA8	Heat Shock Protein Family A (Hsp70) Member 8	Protein Coding	46	GC11M123057	1.64833653
MAVS	Mitochondrial Antiviral Signaling Protein	Protein Coding	37	GC20P004265	1.642849922
OGT	O-Linked N-Acetylglucosamine (GlcNAc) Transferase	Protein Coding	43	GC0XP071534	1.639873743
RETREG1	Reticulophagy Regulator 1	Protein Coding	30	GC05M016472	1.639873743
CHCHD3	Coiled-Coil-Helix-Coiled-Coil-Helix Domain Containing 3	Protein Coding	36	GC07M132784	1.637634277
GABARAPL1	GABA Type A Receptor Associated Protein Like 1	Protein Coding	40	GC12P010212	1.634231448
PARL	Presenilin Associated Rhomboid Like	Protein Coding	41	GC03M183826	1.626164198
TSC2	TSC Complex Subunit 2	Protein Coding	48	GC16P011696	1.613154292
HK2	Hexokinase 2	Protein Coding	45	GC02P074833	1.604134083
VDAC3	Voltage Dependent Anion Channel 3	Protein Coding	42	GC08P042392	1.601760507
MAPK1	Mitogen-Activated Protein Kinase 1	Protein Coding	49	GC22M021759	1.598525643
TSPO	Translocator Protein	Protein Coding	43	GC22P043151	1.587952018
FOXO3	Forkhead Box O3	Protein Coding	44	GC06P108559	1.572308183
MAP2K1	Mitogen-Activated Protein Kinase Kinase 1	Protein Coding	51	GC15P066386	1.568232536
MDH1	Malate Dehydrogenase 1	Protein Coding	44	GC02P063557	1.563021302
ATAD3A	ATPase Family AAA Domain Containing 3A	Protein Coding	40	GC01P003886	1.553566575
TBC1D15	TBC1 Domain Family Member 15	Protein Coding	36	GC12P071839	1.541822195
OCIAD1	OCIA Domain Containing 1	Protein Coding	35	GC04P048805	1.541822195
USP8	Ubiquitin Specific Peptidase 8	Protein Coding	46	GC15P050424	1.538779259
PI4K2A	Phosphatidylinositol 4-Kinase Type 2 Alpha	Protein Coding	39	GC10P097640	1.525234222
KRT15	Keratin 15	Protein Coding	38	GC17M041513	1.525234222
STOM	Stomatin	Protein Coding	37	GC09M121338	1.525234222
BLOC1S1	Biogenesis Of Lysosomal Organelles Complex 1 Subunit 1	Protein Coding	35	GC12P055894	1.525234222
HIF1A	Hypoxia Inducible Factor 1 Subunit Alpha	Protein Coding	45	GC14P061695	1.52385354
ATM	ATM Serine/Threonine Kinase	Protein Coding	50	GC11P108222	1.521288157
LRPPRC	Leucine Rich Pentatricopeptide Repeat Containing	Protein Coding	42	GC02M043886	1.508531094
ATP1B1	ATPase Na+/K+ Transporting Subunit Beta 1	Protein Coding	46	GC01P169105	1.503952265
APP	Amyloid Beta Precursor Protein	Protein Coding	49	GC21M025880	1.47420752
BCAS3	BCAS3 Microtubule Associated Cell Migration Factor	Protein Coding	37	GC17P060678	1.469820619
PHAF1	Phagosome Assembly Factor 1	Protein Coding	25	GC16P067110	1.469820619
ZFYVE16	Zinc Finger FYVE-Type Containing 16	Protein Coding	38	GC05P080407	1.453692436
BET1	Bet1 Golgi Vesicular Membrane Trafficking Protein	Protein Coding	37	GC07M093962	1.453692436
GDAP1	Ganglioside Induced Differentiation Associated Protein 1	Protein Coding	42	GC08P074315	1.437648892
WDR26	WD Repeat Domain 26	Protein Coding	40	GC01M224385	1.414417744
MCL1	MCL1 Apoptosis Regulator, BCL2 Family Member	Protein Coding	45	GC01M152066	1.400294185
LINC-PINT	Long Intergenic Non-Protein Coding RNA, P53 Induced Transcript	RNA Gene	18	GC07M131004	1.39254427
NR4A1	Nuclear Receptor Subfamily 4 Group A Member 1	Protein Coding	45	GC12P052022	1.390671253
HSPD1	Heat Shock Protein Family D (Hsp60) Member 1	Protein Coding	46	GC02M197486	1.3745327
HTT	Huntingtin	Protein Coding	42	GC04P003041	1.374489307
ATG16L1	Autophagy Related 16 Like 1	Protein Coding	41	GC02P233253	1.374489307
TFEB	Transcription Factor EB	Protein Coding	40	GC06M066171	1.360960484
NIPSNAP1	Nipsnap Homolog 1	Protein Coding	38	GC22M029554	1.360960484
BAG6	BAG Cochaperone 6	Protein Coding	36	GC06M031639	1.360960484
CDC37	Cell Division Cycle 37, HSP90 Cochaperone	Protein Coding	40	GC19M010391	1.34890902
PTEN	Phosphatase And Tensin Homolog	Protein Coding	50	GC10P092487	1.346855283
MST1	Macrophage Stimulating 1	Protein Coding	45	GC03M049683	1.346855283
SIRT3	Sirtuin 3	Protein Coding	45	GC11M000215	1.346855283
ATG2B	Autophagy Related 2B	Protein Coding	34	GC14M101208	1.339164138
TGFB1	Transforming Growth Factor Beta 1	Protein Coding	49	GC19M041301	1.332149148
NFE2L2	NFE2 Like BZIP Transcription Factor 2	Protein Coding	48	GC02M177227	1.332149148
UQCRC2	Ubiquinol-Cytochrome C Reductase Core Protein 2	Protein Coding	45	GC16P022240	1.332149148
NIPSNAP2	Nipsnap Homolog 2	Protein Coding	30	GC07P056688	1.332149148
TFE3	Transcription Factor Binding To IGHM Enhancer 3	Protein Coding	42	GC0XM049028	1.316757917
VAPA	VAMP Associated Protein A	Protein Coding	41	GC18P009904	1.316757917
CAV1	Caveolin 1	Protein Coding	46	GC07P116524	1.300575852
TOM1	Target Of Myb1 Membrane Trafficking Protein	Protein Coding	42	GC22P035299	1.300575852
NME4	NME/NM23 Nucleoside Diphosphate Kinase 4	Protein Coding	40	GC16P000396	1.300575852
USP36	Ubiquitin Specific Peptidase 36	Protein Coding	37	GC17M078787	1.300575852
UBXN1	UBX Domain Protein 1	Protein Coding	34	GC11M089634	1.300575852
RMC1	Regulator Of MON1-CCZ1	Protein Coding	24	GC18P023504	1.300575852
RNF31	Ring Finger Protein 31	Protein Coding	40	GC14P024146	1.298311234
TCHP	Trichoplein Keratin Filament Binding	Protein Coding	34	GC12P109900	1.298311234
MTOR	Mechanistic Target Of Rapamycin Kinase	Protein Coding	51	GC01M011106	1.283466935
GPD2	Glycerol-3-Phosphate Dehydrogenase 2	Protein Coding	45	GC02P156401	1.283466935
SHC1	SHC Adaptor Protein 1	Protein Coding	42	GC01M154962	1.283466935
USP33	Ubiquitin Specific Peptidase 33	Protein Coding	38	GC01M077695	1.283466935
STX17	Syntaxin 17	Protein Coding	33	GC09P099906	1.283466935
RNF121	Ring Finger Protein 121	Protein Coding	32	GC11P071960	1.283466935
EPHA2	EPH Receptor A2	Protein Coding	50	GC01M016124	1.265250802
SLC12A6	Solute Carrier Family 12 Member 6	Protein Coding	48	GC15M034229	1.265250802
ITCH	Itchy E3 Ubiquitin Protein Ligase	Protein Coding	44	GC20P034363	1.265250802
SLC12A4	Solute Carrier Family 12 Member 4	Protein Coding	44	GC16M068098	1.265250802
FADS2	Fatty Acid Desaturase 2	Protein Coding	43	GC11P061792	1.265250802
STEAP3	STEAP3 Metalloreductase	Protein Coding	43	GC02P122521	1.265250802
UBE2G2	Ubiquitin Conjugating Enzyme E2 G2	Protein Coding	42	GC21M044768	1.265250802

VPS35	VPS35 Retromer Complex Component	Protein Coding	42	GC16M046992	1.265250802
ANO6	Anoctamin 6	Protein Coding	40	GC12P045215	1.265250802
ARL6IP5	ADP Ribosylation Factor Like GTPase 6 Interacting Protein 5	Protein Coding	36	GC03P069084	1.265250802
EMC1	ER Membrane Protein Complex Subunit 1	Protein Coding	36	GC01M019215	1.265250802
LPCAT3	Lysophosphatidylcholine Acyltransferase 3	Protein Coding	34	GC12M006976	1.265250802
MON2	MON2 Homolog, Regulator Of Endosome-To-Golgi Trafficking	Protein Coding	32	GC12P062466	1.265250802
AMFR	Autocrine Motility Factor Receptor	Protein Coding	41	GC16M056361	1.25146246
SNX3	Sorting Nexin 3	Protein Coding	40	GC06M108211	1.245679855
REEP5	Receptor Accessory Protein 5	Protein Coding	39	GC05M112876	1.245679855
AKAP1	A-Kinase Anchoring Protein 1	Protein Coding	38	GC17P057085	1.245679855
CHMP2A	Charged Multivesicular Body Protein 2A	Protein Coding	38	GC19M058551	1.245679855
GDI2	GDP Dissociation Inhibitor 2	Protein Coding	38	GC10M005765	1.245679855
ESYT1	Extended Synaptotagmin 1	Protein Coding	37	GC12P057267	1.245679855
SPG21	SPG21 Abhydrolase Domain Containing, Maspardin	Protein Coding	37	GC15M064963	1.245679855
ATG2A	Autophagy Related 2A	Protein Coding	36	GC11M089735	1.245679855
SEC22B	SEC22 Homolog B, Vesicle Trafficking Protein	Protein Coding	34	GC01M120150	1.245679855
ARMCX3	Armadillo Repeat Containing X-Linked 3	Protein Coding	33	GC0XP101622	1.245679855
TBC1D17	TBC1 Domain Family Member 17	Protein Coding	33	GC19P049877	1.245679855
RMDN3	Regulator Of Microtubule Dynamics 3	Protein Coding	32	GC15M040735	1.245679855
CCZ1B	CCZ1 Homolog B, Vacuolar Protein Trafficking And Biogenesis Associated	Protein Coding	28	GC07M006794	1.245679855
SREBF2	Sterol Regulatory Element Binding Transcription Factor 2	Protein Coding	41	GC22P041833	1.224397898
FLOT2	Flotillin 2	Protein Coding	40	GC17M035142	1.224397898
SH3GLB1	SH3 Domain Containing GRB2 Like, Endophilin B1	Protein Coding	40	GC01P086704	1.224397898
MIR155	MicroRNA 155	RNA Gene	21	GC21P025573	1.224397898
PPARGC1A	PPARG Coactivator 1 Alpha	Protein Coding	44	GC04M023755	1.209079027
HSPA1L	Heat Shock Protein Family A (Hsp70) Member 1 Like	Protein Coding	44	GC06M031809	1.20085752
HMGB1	High Mobility Group Box 1	Protein Coding	44	GC13M030456	1.187141061
MAP2K3	Mitogen-Activated Protein Kinase Kinase 3	Protein Coding	46	GC17P054789	1.174138069
LMAN1	Lectin, Mannose Binding 1	Protein Coding	44	GC18M059327	1.174138069
FBXW7	F-Box And WD Repeat Domain Containing 7	Protein Coding	43	GC04M152321	1.174138069
ARIH2	Ariadne RBR E3 Ubiquitin Protein Ligase 2	Protein Coding	39	GC03P048918	1.174138069
ATP5IF1	ATP Synthase Inhibitory Factor Subunit 1	Protein Coding	29	GC01P028237	1.168924928
MAP2K2	Mitogen-Activated Protein Kinase Kinase 2	Protein Coding	50	GC19M004090	1.163404942
TIMM23	Translocase Of Inner Mitochondrial Membrane 23	Protein Coding	33	GC10P045972	1.149871707
UBXN6	UBX Domain Protein 6	Protein Coding	36	GC19M004444	1.1493541
FTMT	Ferritin Mitochondrial	Protein Coding	37	GC05P121851	1.142180562
HSPA9	Heat Shock Protein Family A (Hsp70) Member 9	Protein Coding	45	GC05M138554	1.119774342
NRF1	Nuclear Respiratory Factor 1	Protein Coding	42	GC07P129611	1.119774342
KRAS	KRAS Proto-Oncogene, GTPase	Protein Coding	49	GC12M025204	1.104531765
PRKAA2	Protein Kinase AMP-Activated Catalytic Subunit Alpha 2	Protein Coding	49	GC01P056645	1.104531765
AKT1	AKT Serine/Threonine Kinase 1	Protein Coding	50	GC14M104769	1.103592277
RCAN1	Regulator Of Calcineurin 1	Protein Coding	41	GC21M034513	1.103592277
RB1CC1	RB1 Inducible Coiled-Coil 1	Protein Coding	40	GC08M052622	1.103592277
WIPI2	WD Repeat Domain, Phosphoinositide Interacting 2	Protein Coding	40	GC07P005190	1.101327658
ZFYVE1	Zinc Finger FYVE-Type Containing 1	Protein Coding	36	GC14M072969	1.101327658
TIMM44	Translocase Of Inner Mitochondrial Membrane 44	Protein Coding	38	GC19M007926	1.081223369
HRAS	HRas Proto-Oncogene, GTPase	Protein Coding	50	GC11M003189	1.068267226
TFRC	Transferrin Receptor	Protein Coding	47	GC03M196027	1.068267226
NDUFA10	NADH:Ubiquinone Oxidoreductase Subunit A10	Protein Coding	43	GC02M239893	1.068267226
USP35	Ubiquitin Specific Peptidase 35	Protein Coding	33	GC11P078188	1.067651033
ALB	Albumin	Protein Coding	48	GC04P073397	1.053545952
TUFM	Tu Translation Elongation Factor, Mitochondrial	Protein Coding	45	GC16M036978	1.053545952
PRPF8	Pre-MRNA Processing Factor 8	Protein Coding	41	GC17M001650	1.053545952
BAX	BCL2 Associated X, Apoptosis Regulator	Protein Coding	47	GC19P048954	1.04869628
VIM	Vimentin	Protein Coding	48	GC10P017227	1.038839817
MIR106B	MicroRNA 106b	RNA Gene	22	GC07M101892	1.038839817
MIR93	MicroRNA 93	RNA Gene	22	GC07M101891	1.038839817
MIR25	MicroRNA 25	RNA Gene	21	GC07M100093	1.038839817
HK1	Hexokinase 1	Protein Coding	48	GC10P069269	1.027414322
IGF1	Insulin Like Growth Factor 1	Protein Coding	46	GC12M102395	1.027414322
NDUFV1	NADH:Ubiquinone Oxidoreductase Core Subunit V1	Protein Coding	44	GC11P070169	1.027414322
OSBPL5	Oxysterol Binding Protein Like 5	Protein Coding	38	GC11M003327	1.027414322
RDH13	Retinol Dehydrogenase 13	Protein Coding	38	GC19M055039	1.027414322
TAX1BP1	Tax1 Binding Protein 1	Protein Coding	38	GC07P027739	1.027414322
CKAP4	Cytoskeleton Associated Protein 4	Protein Coding	36	GC12M106237	1.027414322
PRKCD	Protein Kinase C Delta	Protein Coding	50	GC03P053156	1.007266521
BIRC2	Baculoviral IAP Repeat Containing 2	Protein Coding	44	GC11P102347	1.007266521
HSPA1A	Heat Shock Protein Family A (Hsp70) Member 1A	Protein Coding	44	GC06P083713	1.007266521
TXN	Thioredoxin	Protein Coding	44	GC09M110243	1.007266521
TRAP1	TNF Receptor Associated Protein 1	Protein Coding	42	GC16M007296	1.007266521
STOML2	Stomatin Like 2	Protein Coding	38	GC09M035099	1.007266521
ATAD3B	ATPase Family AAA Domain Containing 3B	Protein Coding	36	GC01P003884	1.007266521
MRPS2	Mitochondrial Ribosomal Protein S2	Protein Coding	36	GC09P135499	1.00485146
SCO2	Synthesis Of Cytochrome C Oxidase 2	Protein Coding	43	GC22M050523	1.003873944
BAK1	BCL2 Antagonist/Killer 1	Protein Coding	42	GC06M033572	1.003873944
HCCS	Holocytochrome C Synthase	Protein Coding	41	GC0XP011111	1.003873944
WDR45	WD Repeat Domain 45	Protein Coding	39	GC0XM049074	1.003873944
GOSR1	Golgi SNAP Receptor Complex Member 1	Protein Coding	38	GC17P030477	1.003873944
ATP6V1G1	ATPase H+ Transporting V1 Subunit G1	Protein Coding	37	GC09P118943	1.003873944
CHCHD2P9	Coiled-Coil-Helix-Coiled-Coil-Helix Domain Containing 2 Pseudogene 9	Pseudogene	15	GC09P079391	1.003873944
BAG4	BAG Cochaperone 4	Protein Coding	37	GC08P038176	1.001420856
SIAH3	Siah E3 Ubiquitin Protein Ligase Family Member 3	Protein Coding	29	GC13M045777	1.001420856
FLT3	Fms Related Receptor Tyrosine Kinase 3	Protein Coding	50	GC13M028003	0.990157545
SIAH1	Siah E3 Ubiquitin Protein Ligase 1	Protein Coding	45	GC16M048357	0.990157545
GAK	Cyclin G Associated Kinase	Protein Coding	44	GC04M000849	0.990157545
BBC3	BCL2 Binding Component 3	Protein Coding	38	GC19M047220	0.990157545
PRCC	Proline Rich Mitotic Checkpoint Control Factor	Protein Coding	37	GC01P156750	0.990157545
MCU	Mitochondrial Calcium Uniporter	Protein Coding	35	GC10P072692	0.990157545
RIPK1	Receptor Interacting Serine/Threonine Kinase 1	Protein Coding	46	GC06P003275	0.977154493
UQCRC1	Ubiquinol-Cytochrome C Reductase Core Protein 1	Protein Coding	42	GC03M048598	0.977154493
BST2	Bone Marrow Stromal Cell Antigen 2	Protein Coding	38	GC19M017403	0.977154493
ADAM17	ADAM Metallopeptidase Domain 17	Protein Coding	49	GC02M009488	0.971941411
GAPDH	Glyceraldehyde-3-Phosphate Dehydrogenase	Protein Coding	47	GC12P021056	0.971941411
MYH9	Myosin Heavy Chain 9	Protein Coding	47	GC22M036281	0.971941411
SPTAN1	Spectrin Alpha, Non-Erythrocytic 1	Protein Coding	46	GC09P128552	0.971941411
FUS	FUS RNA Binding Protein	Protein Coding	43	GC16P031180	0.971941411
EEF1A1	Eukaryotic Translation Elongation Factor 1 Alpha 1	Protein Coding	42	GC06M073515	0.971941411
UBE2D2	Ubiquitin Conjugating Enzyme E2 D2	Protein Coding	42	GC05P139526	0.971941411

MSRB2	Methionine Sulfoxide Reductase B2	Protein Coding	40	GC10P023095	0.971941411
ARIH1	Ariadne RBR E3 Ubiquitin Protein Ligase 1	Protein Coding	39	GC15P072474	0.971941411
MAPK9	Mitogen-Activated Protein Kinase 9	Protein Coding	46	GC05M180254	0.963674545
DNAJB2	DnaJ Heat Shock Protein Family (Hsp40) Member B2	Protein Coding	41	GC02P219279	0.954590082
DSP	Desmoplakin	Protein Coding	48	GC06P007541	0.952370524
ACTB	Actin Beta	Protein Coding	47	GC07M005527	0.952370524
YWHAE	Tyrosine 3-Monooxygenase/Tryptophan 5-Monooxygenase Activation Protein Epsilon	Protein Coding	47	GC17M003080	0.952370524
CD55	CD55 Molecule (Cromer Blood Group)	Protein Coding	45	GC01P207321	0.952370524
UBE2N	Ubiquitin Conjugating Enzyme E2 N	Protein Coding	45	GC12M093406	0.952370524
SPTBN1	Spectrin Beta, Non-Erythrocytic 1	Protein Coding	44	GC02P054456	0.952370524
A2M	Alpha-2-Macroglobulin	Protein Coding	43	GC12M009067	0.952370524
LAMP2	Lysosomal Associated Membrane Protein 2	Protein Coding	42	GC0XM120426	0.952370524
PLEC	Plectin	Protein Coding	42	GC08M144188	0.952370524
SLC25A24	Solute Carrier Family 25 Member 24	Protein Coding	42	GC01M108134	0.952370524
HNRNPD	Heterogeneous Nuclear Ribonucleoprotein D	Protein Coding	41	GC04M082352	0.952370524
SPTBN2	Spectrin Beta, Non-Erythrocytic 2	Protein Coding	41	GC11M089939	0.952370524
TRIM25	Tripartite Motif Containing 25	Protein Coding	41	GC17M056836	0.952370524
TUBA1C	Tubulin Alpha 1c	Protein Coding	41	GC12P049188	0.952370524
LMO7	LIM Domain 7	Protein Coding	40	GC13P075620	0.952370524
CHDH	Choline Dehydrogenase	Protein Coding	39	GC03M053812	0.952370524
DBN1	Drebrin 1	Protein Coding	39	GC05M177456	0.952370524
IL24	Interleukin 24	Protein Coding	39	GC01P206897	0.952370524
VPS29	VPS29 Retromer Complex Component	Protein Coding	39	GC12M110491	0.952370524
NEXN	Nexilin F-Actin Binding Protein	Protein Coding	38	GC01P077898	0.952370524
A1BG	Alpha-1-B Glycoprotein	Protein Coding	37	GC19M058345	0.952370524
DCD	Dermcidin	Protein Coding	37	GC12M054644	0.952370524
DDRGK1	DDRGK Domain Containing 1	Protein Coding	37	GC20M003344	0.952370524
IL25	Interleukin 25	Protein Coding	36	GC14P032705	0.952370524
GADD45GIP1	GADD45G Interacting Protein 1	Protein Coding	35	GC19M012953	0.952370524
SPECC1	Sperm Antigen With Calponin Homology And Coiled-Coil Domains 1	Protein Coding	33	GC17P054745	0.952370524
MIR181A1	MicroRNA 181a-1	RNA Gene	20	GC01M198860	0.952370524
DDX58	DExD/H-Box Helicase 58	Protein Coding	45	GC09M032455	0.94546032
DUSP1	Dual Specificity Phosphatase 1	Protein Coding	45	GC05M172768	0.94546032
IAPP	Islet Amyloid Polypeptide	Protein Coding	41	GC12P021354	0.94546032
AURKA	Aurora Kinase A	Protein Coding	49	GC20M056370	0.931088448
CDKN2A	Cyclin Dependent Kinase Inhibitor 2A	Protein Coding	48	GC09M021967	0.931088448
ITGA6	Integrin Subunit Alpha 6	Protein Coding	48	GC02P172427	0.931088448
CREB1	CAMP Responsive Element Binding Protein 1	Protein Coding	47	GC02P207529	0.931088448
JUP	Junction Plakoglobin	Protein Coding	46	GC17M041754	0.931088448
MAPK3	Mitogen-Activated Protein Kinase 3	Protein Coding	46	GC16M037120	0.931088448
EIF2AK2	Eukaryotic Translation Initiation Factor 2 Alpha Kinase 2	Protein Coding	45	GC02M037099	0.931088448
TARDBP	TAR DNA Binding Protein	Protein Coding	45	GC01P011013	0.931088448
ATP6V1A	ATPase H+ Transporting V1 Subunit A	Protein Coding	44	GC03P113747	0.931088448
DISC1	DISC1 Scaffold Protein	Protein Coding	44	GC01P231626	0.931088448
DSG2	Desmoglein 2	Protein Coding	44	GC18P031498	0.931088448
ITPR3	Inositol 1,4,5-Trisphosphate Receptor Type 3	Protein Coding	44	GC06P033620	0.931088448
NUP155	Nucleoporin 155	Protein Coding	44	GC05M037288	0.931088448
TRAF6	TNF Receptor Associated Factor 6	Protein Coding	44	GC11M036467	0.931088448
USP14	Ubiquitin Specific Peptidase 14	Protein Coding	44	GC18P000158	0.931088448
RAB5A	RAB5A, Member RAS Oncogene Family	Protein Coding	43	GC03P019948	0.931088448
RUVBL1	RuvB Like AAA ATPase 1	Protein Coding	43	GC03M128064	0.931088448
SEC23A	SEC23 Homolog A, COPII Coat Complex Component	Protein Coding	43	GC14M039031	0.931088448
UBE2D1	Ubiquitin Conjugating Enzyme E2 D1	Protein Coding	43	GC10P058334	0.931088448
VAPB	VAMP Associated Protein B And C	Protein Coding	43	GC20P058389	0.931088448
FUT8	Fucosyltransferase 8	Protein Coding	42	GC14P065411	0.931088448
PFAS	Phosphoribosylformylglycinamidine Synthase	Protein Coding	42	GC17P008247	0.931088448
DST	Dystonin	Protein Coding	41	GC06M056457	0.931088448
RAB10	RAB10, Member RAS Oncogene Family	Protein Coding	41	GC02P026033	0.931088448
BCAP31	B Cell Receptor Associated Protein 31	Protein Coding	40	GC0XM153701	0.931088448
C9orf72	C9orf72-SMCR8 Complex Subunit	Protein Coding	40	GC09M028335	0.931088448
HNRNPDL	Heterogeneous Nuclear Ribonucleoprotein D Like	Protein Coding	40	GC04M082422	0.931088448
HSPA4	Heat Shock Protein Family A (Hsp70) Member 4	Protein Coding	40	GC05P133051	0.931088448
MTCH2	Mitochondrial Carrier 2	Protein Coding	40	GC11M047604	0.931088448
RAB1B	RAB1B, Member RAS Oncogene Family	Protein Coding	40	GC11P070039	0.931088448
SLC3A2	Solute Carrier Family 3 Member 2	Protein Coding	40	GC11P062856	0.931088448
VPS13A	Vacuolar Protein Sorting 13 Homolog A	Protein Coding	40	GC09P077177	0.931088448
DPYSL5	Dihydropyrimidinase Like 5	Protein Coding	39	GC02P026847	0.931088448
HDGF	Heparin Binding Growth Factor	Protein Coding	39	GC01M156912	0.931088448
UXS1	UDP-Glucuronate Decarboxylase 1	Protein Coding	38	GC02M106094	0.931088448
AJUBA	Ajuba LIM Protein	Protein Coding	37	GC14M022971	0.931088448
EIF3C	Eukaryotic Translation Initiation Factor 3 Subunit C	Protein Coding	37	GC16P040885	0.931088448
IL33	Interleukin 33	Protein Coding	37	GC09P006572	0.931088448
SEC61B	SEC61 Translocon Subunit Beta	Protein Coding	37	GC09P099222	0.931088448
SH3GLB2	SH3 Domain Containing GRB2 Like, Endophilin B2	Protein Coding	37	GC09M129007	0.931088448
SLC25A46	Solute Carrier Family 25 Member 46	Protein Coding	37	GC05P110738	0.931088448
MTFR1	Mitochondrial Fission Regulator 1	Protein Coding	36	GC08P065643	0.931088448
RAI14	Retinoic Acid Induced 14	Protein Coding	36	GC05P034656	0.931088448
MON1B	MON1 Homolog B, Secretory Trafficking Associated	Protein Coding	34	GC16P077190	0.931088448
CCZ1	CCZ1 Homolog, Vacuolar Protein Trafficking And Biogenesis Associated	Protein Coding	32	GC07P005898	0.931088448
MEG3	Maternally Expressed 3	RNA Gene	29	GC14P109962	0.931088448
MIR320A	MicroRNA 320a	RNA Gene	21	GC08M022268	0.931088448
MIR410	MicroRNA 410	RNA Gene	18	GC14P109990	0.931088448
MAPK10	Mitogen-Activated Protein Kinase 10	Protein Coding	48	GC04M085990	0.922369838
LDHA	Lactate Dehydrogenase A	Protein Coding	49	GC11P018394	0.907548189
PSMB8	Proteasome 20S Subunit Beta 8	Protein Coding	48	GC06M032840	0.907548189
TRPV4	Transient Receptor Potential Cation Channel Subfamily V Member 4	Protein Coding	48	GC12M109783	0.907548189
ABCC1	ATP Binding Cassette Subfamily C Member 1	Protein Coding	47	GC16P015949	0.907548189
FBP1	Fructose-Bisphosphatase 1	Protein Coding	47	GC09M094603	0.907548189
NPC1	NPC Intracellular Cholesterol Transporter 1	Protein Coding	47	GC18M023506	0.907548189
CPT1A	Carnitine Palmitoyltransferase 1A	Protein Coding	46	GC11M068754	0.907548189
CYCS	Cytochrome C, Somatic	Protein Coding	46	GC07M025118	0.907548189
IGF2R	Insulin Like Growth Factor 2 Receptor	Protein Coding	46	GC06P159969	0.907548189
KEAP1	Kelch Like ECH Associated Protein 1	Protein Coding	46	GC19M010486	0.907548189
PDE2A	Phosphodiesterase 2A	Protein Coding	46	GC11M072576	0.907548189
ADH5	Alcohol Dehydrogenase 5 (Class III), Chi Polypeptide	Protein Coding	45	GC04M099070	0.907548189
APAF1	Apoptotic Peptidase Activating Factor 1	Protein Coding	45	GC12P098645	0.907548189
CAMKK2	Calcium/Calmodulin Dependent Protein Kinase Kinase 2	Protein Coding	45	GC12M122269	0.907548189
DCN	Decorin	Protein Coding	45	GC12M091140	0.907548189

ENO1	Enolase 1	Protein Coding	45	GC01M008861	0.907548189
MYH2	Myosin Heavy Chain 2	Protein Coding	45	GC17M010521	0.907548189
YAP1	Yes1 Associated Transcriptional Regulator	Protein Coding	45	GC11P102110	0.907548189
YWHAZ	Tyrosine 3-Monooxygenase/Tryptophan 5-Monooxygenase Activation Protein Zeta	Protein Coding	45	GC08M100917	0.907548189
EPS8	Epidermal Growth Factor Receptor Pathway Substrate 8	Protein Coding	44	GC12M021188	0.907548189
NEFL	Neurofilament Light Chain	Protein Coding	44	GC08M024950	0.907548189
NNT	Nicotinamide Nucleotide Transhydrogenase	Protein Coding	44	GC05P043602	0.907548189
RAD23B	RAD23 Homolog B, Nucleotide Excision Repair Protein	Protein Coding	44	GC09P107283	0.907548189
SLC25A20	Solute Carrier Family 25 Member 20	Protein Coding	44	GC03M048869	0.907548189
SMARCAD1	SWI/SNF-Related, Matrix-Associated Actin-Dependent Regulator Of Chromatin, Subfamily A, Containing DEAD/H Box 1	Protein Coding	44	GC04P094207	0.907548189
VCAM1	Vascular Cell Adhesion Molecule 1	Protein Coding	44	GC01P100719	0.907548189
XBP1	X-Box Binding Protein 1	Protein Coding	44	GC22M028794	0.907548189
PEX2	Peroxisomal Biogenesis Factor 2	Protein Coding	43	GC08M076980	0.907548189
RTN4	Reticulon 4	Protein Coding	43	GC02M054934	0.907548189
ABCC5	ATP Binding Cassette Subfamily C Member 5	Protein Coding	42	GC03M183919	0.907548189
ACSL4	Acyl-CoA Synthetase Long Chain Family Member 4	Protein Coding	42	GC0XM109624	0.907548189
APEX1	Apurinic/Apyrimidinic Endodeoxyribonuclease 1	Protein Coding	42	GC14P020455	0.907548189
ATP12A	ATPase H+/K+ Transporting Non-Gastric Alpha2 Subunit	Protein Coding	42	GC13P024680	0.907548189
B3GAT3	Beta-1,3-Glucuronyltransferase 3	Protein Coding	42	GC11M089626	0.907548189
COL11A1	Collagen Type XI Alpha 1 Chain	Protein Coding	42	GC01M102876	0.907548189
EMD	Emerin	Protein Coding	42	GC0XP154379	0.907548189
UCP1	Uncoupling Protein 1	Protein Coding	42	GC04M140559	0.907548189
ACSL5	Acyl-CoA Synthetase Long Chain Family Member 5	Protein Coding	41	GC10P112374	0.907548189
CAV2	Caveolin 2	Protein Coding	41	GC07P116287	0.907548189
COX15	Cytochrome C Oxidase Assembly Homolog COX15	Protein Coding	41	GC10M099696	0.907548189
HS2ST1	Heparan Sulfate 2-O-Sulfotransferase 1	Protein Coding	41	GC01P086914	0.907548189
SLC31A1	Solute Carrier Family 31 Member 1	Protein Coding	41	GC09P113221	0.907548189
SLC35A3	Solute Carrier Family 35 Member A3	Protein Coding	41	GC01P099987	0.907548189
TSG101	Tumor Susceptibility 101	Protein Coding	41	GC11M018468	0.907548189
ABCB10	ATP Binding Cassette Subfamily B Member 10	Protein Coding	40	GC01M229516	0.907548189
BAIAP2	BAR/IMD Domain Containing Adaptor Protein 2	Protein Coding	40	GC17P081035	0.907548189
CDIPT	CDP-Diacylglycerol--Inositol 3-Phosphatidyltransferase	Protein Coding	40	GC16M037106	0.907548189
E2F3	E2F Transcription Factor 3	Protein Coding	40	GC06P020402	0.907548189
PLEKHA1	Pleckstrin Homology Domain Containing A1	Protein Coding	40	GC10P122374	0.907548189
STX16	Syntaxin 16	Protein Coding	40	GC20P058652	0.907548189
ORMDL3	ORMDL Sphingolipid Biosynthesis Regulator 3	Protein Coding	39	GC17M039921	0.907548189
SDCBP	Syndecan Binding Protein	Protein Coding	39	GC08P058539	0.907548189
SIRT4	Sirtuin 4	Protein Coding	39	GC12P120291	0.907548189
TAOK2	TAO Kinase 2	Protein Coding	39	GC16P041023	0.907548189
ATG3	Autophagy Related 3	Protein Coding	38	GC03M112532	0.907548189
CHMP5	Charged Multivesicular Body Protein 5	Protein Coding	38	GC09P033257	0.907548189
CORO1C	Coronin 1C	Protein Coding	38	GC12M108645	0.907548189
DDX39B	DExD-Box Helicase 39B	Protein Coding	38	GC06M031530	0.907548189
ERGIC1	Endoplasmic Reticulum-Golgi Intermediate Compartment 1	Protein Coding	38	GC05P172834	0.907548189
GALNT1	Polypeptide N-Acetylgalactosaminyltransferase 1	Protein Coding	38	GC18P035581	0.907548189
GLT8D1	Glycosyltransferase 8 Domain Containing 1	Protein Coding	38	GC03M052694	0.907548189
MTPAP	Mitochondrial Poly(A) Polymerase	Protein Coding	38	GC10M033007	0.907548189
MYO18A	Myosin XVIIIA	Protein Coding	38	GC17M035146	0.907548189
RPL28	Ribosomal Protein L28	Protein Coding	38	GC19P067402	0.907548189
STX8	Syntaxin 8	Protein Coding	38	GC17M009250	0.907548189
TMEM165	Transmembrane Protein 165	Protein Coding	38	GC04P055395	0.907548189
TNRC6A	Trinucleotide Repeat Containing Adaptor 6A	Protein Coding	38	GC16P024611	0.907548189
ATP9B	ATPase Phospholipid Transporting 9B (Putative)	Protein Coding	37	GC18P079069	0.907548189
GHITM	Growth Hormone Inducible Transmembrane Protein	Protein Coding	37	GC10P084139	0.907548189
KPNA5	Karyopherin Subunit Alpha 5	Protein Coding	37	GC06P116681	0.907548189
MYO5C	Myosin VC	Protein Coding	37	GC15M091517	0.907548189
PLSCR3	Phospholipid Scramblase 3	Protein Coding	37	GC17M007389	0.907548189
PRUNE2	Prune Homolog 2 With BCH Domain	Protein Coding	37	GC09M076611	0.907548189
SFXN3	Sideroflexin 3	Protein Coding	37	GC10P101031	0.907548189
TMBIM6	Transmembrane BAX Inhibitor Motif Containing 6	Protein Coding	37	GC12P049707	0.907548189
TMED1	Transmembrane P24 Trafficking Protein 1	Protein Coding	37	GC19M010832	0.907548189
TMX3	Thioredoxin Related Transmembrane Protein 3	Protein Coding	37	GC18M068673	0.907548189
TRAPPC12	Trafficking Protein Particle Complex Subunit 12	Protein Coding	37	GC02P003383	0.907548189
TXNIP	Thioredoxin Interacting Protein	Protein Coding	37	GC01M145992	0.907548189
VPS28	VPS28 Subunit Of ESCRT-I	Protein Coding	37	GC08M145416	0.907548189
CYP20A1	Cytochrome P450 Family 20 Subfamily A Member 1	Protein Coding	36	GC02P203238	0.907548189
DAZAP2	DAZ Associated Protein 2	Protein Coding	36	GC12P051238	0.907548189
FADS3	Fatty Acid Desaturase 3	Protein Coding	36	GC11M061873	0.907548189
PPM1L	Protein Phosphatase, Mg2+/Mn2+ Dependent 1L	Protein Coding	36	GC03P160755	0.907548189
VPS16	VPS16 Core Subunit Of CORVET And HOPS Complexes	Protein Coding	36	GC20P002840	0.907548189
ZC3H7B	Zinc Finger CCCH-Type Containing 7B	Protein Coding	36	GC22P041301	0.907548189
IRGM	Immunity Related GTPase M	Protein Coding	35	GC05P150846	0.907548189
MLF2	Myeloid Leukemia Factor 2	Protein Coding	35	GC12M006912	0.907548189
SEC62	SEC62 Homolog, Preprotein Translocation Factor	Protein Coding	35	GC03P169966	0.907548189
SLC38A7	Solute Carrier Family 38 Member 7	Protein Coding	35	GC16M058665	0.907548189
WDR6	WD Repeat Domain 6	Protein Coding	35	GC03P049007	0.907548189
ACTBL2	Actin Beta Like 2	Protein Coding	34	GC05M057480	0.907548189
OIP5	Opa Interacting Protein 5	Protein Coding	34	GC15M041309	0.907548189
PPP1R18	Protein Phosphatase 1 Regulatory Subunit 18	Protein Coding	34	GC06M065878	0.907548189
PRAF2	PRA1 Domain Family Member 2	Protein Coding	34	GC0XM049258	0.907548189
REEP4	Receptor Accessory Protein 4	Protein Coding	34	GC08M022138	0.907548189
RILP	Rab Interacting Lysosomal Protein	Protein Coding	34	GC17M001646	0.907548189
TMED5	Transmembrane P24 Trafficking Protein 5	Protein Coding	34	GC01M093150	0.907548189
TTC1	Tetratricopeptide Repeat Domain 1	Protein Coding	34	GC05P160009	0.907548189
VPS37C	VPS37C Subunit Of ESCRT-I	Protein Coding	34	GC11M061130	0.907548189
GOLT1B	Golgi Transport 1B	Protein Coding	33	GC12P021501	0.907548189
MFSD10	Major Facilitator Superfamily Domain Containing 10	Protein Coding	33	GC04M002903	0.907548189
MVB12B	Multivesicular Body Subunit 12B	Protein Coding	33	GC09P126326	0.907548189
RNF26	Ring Finger Protein 26	Protein Coding	33	GC11P119334	0.907548189
CCDC127	Coiled-Coil Domain Containing 127	Protein Coding	31	GC05M000204	0.907548189
SARAF	Store-Operated Calcium Entry Associated Regulatory Factor	Protein Coding	31	GC08M030063	0.907548189
RELL1	RELT Like 1	Protein Coding	30	GC04M037592	0.907548189
WDR24	WD Repeat Domain 24	Protein Coding	30	GC16M007028	0.907548189
KIAA1671	KIAA1671	Protein Coding	29	GC22P036129	0.907548189
MTARC1	Mitochondrial Amidoxime Reducing Component 1	Protein Coding	26	GC01P220788	0.907548189
PACC1	Proton Activated Chloride Channel 1	Protein Coding	25	GC01M212365	0.907548189
PEDS1	Plasmanylethanolamine Desaturase 1	Protein Coding	24	GC20M050119	0.907548189
PIP4P2	Phosphatidylinositol-4,5-Bisphosphate 4-Phosphatase 2	Protein Coding	24	GC08M090994	0.907548189

PIK3C3	Phosphatidylinositol 3-Kinase Catalytic Subunit Type 3	Protein Coding	48	GC18P041955	0.904155612
STARD7	StAR Related Lipid Transfer Domain Containing 7	Protein Coding	36	GC02M096184	0.904155612
MYC	MYC Proto-Oncogene, BHLH Transcription Factor	Protein Coding	49	GC08P127735	0.880828738
PIK3R1	Phosphoinositide-3-Kinase Regulatory Subunit 1	Protein Coding	49	GC05P068215	0.880828738
UCHL1	Ubiquitin C-Terminal Hydrolase L1	Protein Coding	49	GC04P041256	0.880828738
GLUD1	Glutamate Dehydrogenase 1	Protein Coding	48	GC10M087050	0.880828738
ITGB1	Integrin Subunit Beta 1	Protein Coding	48	GC10M033116	0.880828738
MAP3K7	Mitogen-Activated Protein Kinase Kinase Kinase 7	Protein Coding	48	GC06M090513	0.880828738
NOTCH2	Notch Receptor 2	Protein Coding	48	GC01M119911	0.880828738
PIK3R2	Phosphoinositide-3-Kinase Regulatory Subunit 2	Protein Coding	48	GC19P018153	0.880828738
POR	Cytochrome P450 Oxidoreductase	Protein Coding	48	GC07P075899	0.880828738
PTPN1	Protein Tyrosine Phosphatase Non-Receptor Type 1	Protein Coding	48	GC20P050510	0.880828738
SCD	Stearoyl-CoA Desaturase	Protein Coding	48	GC10P100347	0.880828738
SLC12A2	Solute Carrier Family 12 Member 2	Protein Coding	48	GC05P128083	0.880828738
SMAD2	SMAD Family Member 2	Protein Coding	48	GC18M047809	0.880828738
DHFR	Dihydrofolate Reductase	Protein Coding	47	GC05M080626	0.880828738
ECE1	Endothelin Converting Enzyme 1	Protein Coding	47	GC01M021217	0.880828738
GLS	Glutaminase	Protein Coding	47	GC02P190880	0.880828738
ITGA5	Integrin Subunit Alpha 5	Protein Coding	47	GC12M054899	0.880828738
ITPR1	Inositol 1,4,5-Trisphosphate Receptor Type 1	Protein Coding	47	GC03P004486	0.880828738
LRP2	LDL Receptor Related Protein 2	Protein Coding	47	GC02M169127	0.880828738
PLD1	Phospholipase D1	Protein Coding	47	GC03M171600	0.880828738
SLC1A1	Solute Carrier Family 1 Member 1	Protein Coding	47	GC09P004490	0.880828738
AK2	Adenylate Kinase 2	Protein Coding	46	GC01M033007	0.880828738
ATP7B	ATPase Copper Transporting Beta	Protein Coding	46	GC13M051930	0.880828738
DGKE	Diacylglycerol Kinase Epsilon	Protein Coding	46	GC17P056834	0.880828738
EIF2AK3	Eukaryotic Translation Initiation Factor 2 Alpha Kinase 3	Protein Coding	46	GC02M088556	0.880828738
GGCX	Gamma-Glutamyl Carboxylase	Protein Coding	46	GC02M085544	0.880828738
GPHN	Gephyrin	Protein Coding	46	GC14P066507	0.880828738
GRN	Granulin Precursor	Protein Coding	46	GC17P044345	0.880828738
MIF	Macrophage Migration Inhibitory Factor	Protein Coding	46	GC22P023894	0.880828738
PI4KA	Phosphatidylinositol 4-Kinase Alpha	Protein Coding	46	GC22M020707	0.880828738
PIKFYVE	Phosphoinositide Kinase, FYVE-Type Zinc Finger Containing	Protein Coding	46	GC02P208266	0.880828738
PRKCI	Protein Kinase C Iota	Protein Coding	46	GC03P170222	0.880828738
SLC29A1	Solute Carrier Family 29 Member 1 (Augustine Blood Group)	Protein Coding	46	GC06P044219	0.880828738
STIM1	Stromal Interaction Molecule 1	Protein Coding	46	GC11P003855	0.880828738
ABCC4	ATP Binding Cassette Subfamily C Member 4	Protein Coding	45	GC13M095019	0.880828738
ATF6	Activating Transcription Factor 6	Protein Coding	45	GC01P161766	0.880828738
ATP7A	ATPase Copper Transporting Alpha	Protein Coding	45	GC0XP077993	0.880828738
HMOX2	Heme Oxygenase 2	Protein Coding	45	GC16P004474	0.880828738
ITGAV	Integrin Subunit Alpha V	Protein Coding	45	GC02P186589	0.880828738
ITPR2	Inositol 1,4,5-Trisphosphate Receptor Type 2	Protein Coding	45	GC12M026336	0.880828738
LBR	Lamin B Receptor	Protein Coding	45	GC01M225401	0.880828738
LNPEP	Leucyl And Cystinyl Aminopeptidase	Protein Coding	45	GC05P096935	0.880828738
ROCK2	Rho Associated Coiled-Coil Containing Protein Kinase 2	Protein Coding	45	GC02M011309	0.880828738
SCARB1	Scavenger Receptor Class B Member 1	Protein Coding	45	GC12M124776	0.880828738
SGPL1	Sphingosine-1-Phosphate Lyase 1	Protein Coding	45	GC10P070815	0.880828738
TSC1	TSC Complex Subunit 1	Protein Coding	45	GC09M132891	0.880828738
WFS1	Wolframin ER Transmembrane Glycoprotein	Protein Coding	45	GC04P006260	0.880828738
WWOX	WW Domain Containing Oxidoreductase	Protein Coding	45	GC16P078099	0.880828738
ALDH3A2	Aldehyde Dehydrogenase 3 Family Member A2	Protein Coding	44	GC17P019648	0.880828738
BCAT2	Branched Chain Amino Acid Transaminase 2	Protein Coding	44	GC19M048795	0.880828738
CDC34	Cell Division Cycle 34, Ubiqiutin Conjugating Enzyme	Protein Coding	44	GC19P002782	0.880828738
CLCN7	Chloride Voltage-Gated Channel 7	Protein Coding	44	GC16M001444	0.880828738
CTNND1	Catenin Delta 1	Protein Coding	44	GC11P058140	0.880828738
EFNB1	Ephrin B1	Protein Coding	44	GC0XP068828	0.880828738
FURIN	Furin, Paired Basic Amino Acid Cleaving Enzyme	Protein Coding	44	GC15P090868	0.880828738
HLA-C	Major Histocompatibility Complex, Class I, C	Protein Coding	44	GC06M065914	0.880828738
PAK2	P21 (RAC1) Activated Kinase 2	Protein Coding	44	GC03P196739	0.880828738
PEX1	Peroxisomal Biogenesis Factor 1	Protein Coding	44	GC07M092487	0.880828738
PIK3R4	Phosphoinositide-3-Kinase Regulatory Subunit 4	Protein Coding	44	GC03M130678	0.880828738
POMGNT1	Protein O-Linked Mannose N-Acetylglucosaminyltransferase 1 (Beta 1,2-)	Protein Coding	44	GC01M046188	0.880828738
SIRT5	Sirtuin 5	Protein Coding	44	GC06P013574	0.880828738
SLC26A2	Solute Carrier Family 26 Member 2	Protein Coding	44	GC05P149944	0.880828738
SLC27A4	Solute Carrier Family 27 Member 4	Protein Coding	44	GC09P128340	0.880828738
TMPO	Thymopoietin	Protein Coding	44	GC12P098515	0.880828738
TRPM7	Transient Receptor Potential Cation Channel Subfamily M Member 7	Protein Coding	44	GC15M050552	0.880828738
UGDH	UDP-Glucose 6-Dehydrogenase	Protein Coding	44	GC04M039502	0.880828738
ABCD3	ATP Binding Cassette Subfamily D Member 3	Protein Coding	43	GC01P094385	0.880828738
ATP6AP2	ATPase H+ Transporting Accessory Protein 2	Protein Coding	43	GC0XP040582	0.880828738
BCL2L2	BCL2 Like 2	Protein Coding	43	GC14P032837	0.880828738
CALM2	Calmodulin 2	Protein Coding	43	GC02M047124	0.880828738
DOCK8	Dedicator Of Cytokinesis 8	Protein Coding	43	GC09P000214	0.880828738
EPS15	Epidermal Growth Factor Receptor Pathway Substrate 15	Protein Coding	43	GC01M051354	0.880828738
EWSR1	EWS RNA Binding Protein 1	Protein Coding	43	GC22P036820	0.880828738
FIG4	FIG4 Phosphoinositide 5-Phosphatase	Protein Coding	43	GC06P109691	0.880828738
GBF1	Golgi Brefeldin A Resistant Guanine Nucleotide Exchange Factor 1	Protein Coding	43	GC10P102245	0.880828738
MAD2L1	Mitotic Arrest Deficient 2 Like 1	Protein Coding	43	GC04M120055	0.880828738
PRKD3	Protein Kinase D3	Protein Coding	43	GC02M037251	0.880828738
SLC12A7	Solute Carrier Family 12 Member 7	Protein Coding	43	GC05M001050	0.880828738
SOAT1	Sterol O-Acyltransferase 1	Protein Coding	43	GC01P179262	0.880828738
SV2A	Synaptic Vesicle Glycoprotein 2A	Protein Coding	43	GC01M149903	0.880828738
YME1L1	YME1 Like 1 ATPase	Protein Coding	43	GC10M027110	0.880828738
ADAM22	ADAM Metallopeptidase Domain 22	Protein Coding	42	GC07P087934	0.880828738
APBB1	Amyloid Beta Precursor Protein Binding Family B Member 1	Protein Coding	42	GC11M006571	0.880828738
ATP6AP1	ATPase H+ Transporting Accessory Protein 1	Protein Coding	42	GC0XP154428	0.880828738
CPD	Carboxypeptidase D	Protein Coding	42	GC17P030378	0.880828738
CXADR	CXADR Ig-Like Cell Adhesion Molecule	Protein Coding	42	GC21P017513	0.880828738
CYP51A1	Cytochrome P450 Family 51 Subfamily A Member 1	Protein Coding	42	GC07M092112	0.880828738
DIAPH2	Diaphanous Related Formin 2	Protein Coding	42	GC0XP096684	0.880828738
FBXW11	F-Box And WD Repeat Domain Containing 11	Protein Coding	42	GC05M171861	0.880828738
GDI1	GDP Dissociation Inhibitor 1	Protein Coding	42	GC0XP154436	0.880828738
JAM3	Junctional Adhesion Molecule 3	Protein Coding	42	GC11P134068	0.880828738
MADD	MAP Kinase Activating Death Domain	Protein Coding	42	GC11P047290	0.880828738
NBEA	Neurobeachin	Protein Coding	42	GC13P034942	0.880828738
NDUFA8	NADH:Ubiquinone Oxidoreductase Subunit A8	Protein Coding	42	GC09M122132	0.880828738
NDUFB10	NADH:Ubiquinone Oxidoreductase Subunit B10	Protein Coding	42	GC16P011690	0.880828738
NISCH	Nischarin	Protein Coding	42	GC03P052455	0.880828738

OCLN	Occludin	Protein Coding	42	GC05P069492	0.880828738
PEX6	Peroxisomal Biogenesis Factor 6	Protein Coding	42	GC06M042963	0.880828738
PTPN13	Protein Tyrosine Phosphatase Non-Receptor Type 13	Protein Coding	42	GC04P086594	0.880828738
RPTOR	Regulatory Associated Protein Of MTOR Complex 1	Protein Coding	42	GC17P080544	0.880828738
SAR1B	Secretion Associated Ras Related GTPase 1B	Protein Coding	42	GC05M134601	0.880828738
SEC63	SEC63 Homolog, Protein Translocation Regulator	Protein Coding	42	GC06M107867	0.880828738
SLC4A2	Solute Carrier Family 4 Member 2	Protein Coding	42	GC07P151057	0.880828738
STT3B	STT3 Oligosaccharyltransferase Complex Catalytic Subunit B	Protein Coding	42	GC03P031550	0.880828738
VANGL1	VANGL Planar Cell Polarity Protein 1	Protein Coding	42	GC01P115641	0.880828738
BCKDHB	Branched Chain Keto Acid Dehydrogenase E1 Subunit Beta	Protein Coding	41	GC06P080106	0.880828738
CD9	CD9 Molecule	Protein Coding	41	GC12P021026	0.880828738
CDC42BPA	CDC42 Binding Protein Kinase Alpha	Protein Coding	41	GC01M226989	0.880828738
CHM	CHM Rab Escort Protein	Protein Coding	41	GC0XM085861	0.880828738
COG2	Component Of Oligomeric Golgi Complex 2	Protein Coding	41	GC01P230642	0.880828738
FAF1	Fas Associated Factor 1	Protein Coding	41	GC01M050439	0.880828738
FLCN	Folliculin	Protein Coding	41	GC17M017212	0.880828738
GOSR2	Golgi SNAP Receptor Complex Member 2	Protein Coding	41	GC17P055888	0.880828738
KIF1A	Kinesin Family Member 1A	Protein Coding	41	GC02M240713	0.880828738
LEMD3	LEM Domain Containing 3	Protein Coding	41	GC12P065169	0.880828738
LRP8	LDL Receptor Related Protein 8	Protein Coding	41	GC01M053243	0.880828738
MCAM	Melanoma Cell Adhesion Molecule	Protein Coding	41	GC11M119308	0.880828738
NEO1	Neogenin 1	Protein Coding	41	GC15P073051	0.880828738
PIGK	Phosphatidylinositol Glycan Anchor Biosynthesis Class K	Protein Coding	41	GC01M077088	0.880828738
PKMYT1	Protein Kinase, Membrane Associated Tyrosine/Threonine 1	Protein Coding	41	GC16M007240	0.880828738
PLXNA1	Plexin A1	Protein Coding	41	GC03P126988	0.880828738
PPM1G	Protein Phosphatase, Mg2+/Mn2+ Dependent 1G	Protein Coding	41	GC02M028129	0.880828738
RAB23	RAB23, Member RAS Oncogene Family	Protein Coding	41	GC06M066403	0.880828738
RABGEF1	RAB Guanine Nucleotide Exchange Factor 1	Protein Coding	41	GC07P070542	0.880828738
RNF14	Ring Finger Protein 14	Protein Coding	41	GC05P146229	0.880828738
SLC33A1	Solute Carrier Family 33 Member 1	Protein Coding	41	GC03M155821	0.880828738
SLC4A7	Solute Carrier Family 4 Member 7	Protein Coding	41	GC03M027372	0.880828738
SLC7A2	Solute Carrier Family 7 Member 2	Protein Coding	41	GC08P017497	0.880828738
SNAP23	Synaptosome Associated Protein 23	Protein Coding	41	GC15P042491	0.880828738
THOP1	Thimet Oligopeptidase 1	Protein Coding	41	GC19P002785	0.880828738
TMEM38B	Transmembrane Protein 38B	Protein Coding	41	GC09P105694	0.880828738
TMEM43	Transmembrane Protein 43	Protein Coding	41	GC03P014124	0.880828738
TRIP11	Thyroid Hormone Receptor Interactor 11	Protein Coding	41	GC14M091965	0.880828738
VAC14	VAC14 Component Of PIKFYVE Complex	Protein Coding	41	GC16M070690	0.880828738
VAMP2	Vesicle Associated Membrane Protein 2	Protein Coding	41	GC17M010433	0.880828738
VPS33B	VPS33B Late Endosome And Lysosome Associated	Protein Coding	41	GC15M090998	0.880828738
VPS37A	VPS37A Subunit Of ESCRT-I	Protein Coding	41	GC08P017246	0.880828738
AAAS	Aladin WD Repeat Nucleoporin	Protein Coding	40	GC12M053307	0.880828738
AKAP12	A-Kinase Anchoring Protein 12	Protein Coding	40	GC06P151239	0.880828738
ARFGEF2	ADP Ribosylation Factor Guanine Nucleotide Exchange Factor 2	Protein Coding	40	GC20P048921	0.880828738
ATL1	Atlastin GTPase 1	Protein Coding	40	GC14P050532	0.880828738
ATP4A	ATPase H+/K+ Transporting Subunit Alpha	Protein Coding	40	GC19M066540	0.880828738
ATP6V1F	ATPase H+ Transporting V1 Subunit F	Protein Coding	40	GC07P128862	0.880828738
CAMLG	Calcium Modulating Ligand	Protein Coding	40	GC05P134738	0.880828738
CISD2	CDGSH Iron Sulfur Domain 2	Protein Coding	40	GC04P102868	0.880828738
COG4	Component Of Oligomeric Golgi Complex 4	Protein Coding	40	GC16M070737	0.880828738
GLE1	GLE1 RNA Export Mediator	Protein Coding	40	GC09P128504	0.880828738
GOLGA2	Golgin A2	Protein Coding	40	GC09M128255	0.880828738
GOPC	Golgi Associated PDZ And Coiled-Coil Motif Containing	Protein Coding	40	GC06M117560	0.880828738
KIDINS220	Kinase D Interacting Substrate 220	Protein Coding	40	GC02M008724	0.880828738
KTN1	Kinectin 1	Protein Coding	40	GC14P055559	0.880828738
LRIG1	Leucine Rich Repeats And Immunoglobulin Like Domains 1	Protein Coding	40	GC03M066379	0.880828738
LRIG2	Leucine Rich Repeats And Immunoglobulin Like Domains 2	Protein Coding	40	GC01P113073	0.880828738
MTMR4	Myotubularin Related Protein 4	Protein Coding	40	GC17M058489	0.880828738
NBAS	NBAS Subunit Of NRZ Tethering Complex	Protein Coding	40	GC02M014783	0.880828738
PDE8A	Phosphodiesterase 8A	Protein Coding	40	GC15P118028	0.880828738
PIGT	Phosphatidylinositol Glycan Anchor Biosynthesis Class T	Protein Coding	40	GC20P045416	0.880828738
PLEKHM1	Pleckstrin Homology And RUN Domain Containing M1	Protein Coding	40	GC17M045435	0.880828738
PPFIBP1	PPFIA Binding Protein 1	Protein Coding	40	GC12P027523	0.880828738
PUM2	Pumilio RNA Binding Family Member 2	Protein Coding	40	GC02M020328	0.880828738
RAB5B	RAB5B, Member RAS Oncogene Family	Protein Coding	40	GC12P055973	0.880828738
RTN3	Reticulon 3	Protein Coding	40	GC11P063702	0.880828738
SCAP	SREBF Chaperone	Protein Coding	40	GC03M047413	0.880828738
SCD5	Stearoyl-CoA Desaturase 5	Protein Coding	40	GC04M082629	0.880828738
SH3BP4	SH3 Domain Binding Protein 4	Protein Coding	40	GC02P234951	0.880828738
SLC35A2	Solute Carrier Family 35 Member A2	Protein Coding	40	GC0XM048903	0.880828738
SLC6A15	Solute Carrier Family 6 Member 15	Protein Coding	40	GC12M084859	0.880828738
STAM	Signal Transducing Adaptor Molecule	Protein Coding	40	GC10P017689	0.880828738
TACC1	Transforming Acidic Coiled-Coil Containing Protein 1	Protein Coding	40	GC08P038728	0.880828738
TOR1AIP1	Torsin 1A Interacting Protein 1	Protein Coding	40	GC01P179882	0.880828738
TPD52	Tumor Protein D52	Protein Coding	40	GC08M080034	0.880828738
UBE2E3	Ubiquitin Conjugating Enzyme E2 E3	Protein Coding	40	GC02P180967	0.880828738
UBE2J1	Ubiquitin Conjugating Enzyme E2 J1	Protein Coding	40	GC06M089326	0.880828738
UBQLN1	Ubiquilin 1	Protein Coding	40	GC09M083659	0.880828738
UVRAG	UV Radiation Resistance Associated	Protein Coding	40	GC11P075815	0.880828738
VAMP7	Vesicle Associated Membrane Protein 7	Protein Coding	40	GC0XP155881	0.880828738
VAMP8	Vesicle Associated Membrane Protein 8	Protein Coding	40	GC02P085561	0.880828738
VPS33A	VPS33A Core Subunit Of CORVET And HOPS Complexes	Protein Coding	40	GC12M122229	0.880828738
VRK2	VRK Serine/Threonine Kinase 2	Protein Coding	40	GC02P057907	0.880828738
VTI1B	Vesicle Transport Through Interaction With T-SNAREs 1B	Protein Coding	40	GC14M067647	0.880828738
ZFYVE9	Zinc Finger FYVE-Type Containing 9	Protein Coding	40	GC01P052142	0.880828738
ANKLE2	Ankyrin Repeat And LEM Domain Containing 2	Protein Coding	39	GC12M132725	0.880828738
CAPNS1	Calpain Small Subunit 1	Protein Coding	39	GC19P066563	0.880828738
DOCK6	Dedicator Of Cytokinesis 6	Protein Coding	39	GC19M011199	0.880828738
FYCO1	FYVE And Coiled-Coil Domain Autophagy Adaptor 1	Protein Coding	39	GC03M045917	0.880828738
HPS3	HPS3 Biogenesis Of Lysosomal Organelles Complex 2 Subunit 1	Protein Coding	39	GC03P149129	0.880828738
HSPA1B	Heat Shock Protein Family A (Hsp70) Member 1B	Protein Coding	39	GC06P083712	0.880828738
INF2	Inverted Formin 2	Protein Coding	39	GC14P109729	0.880828738
LAMTOR2	Late Endosomal/Lysosomal Adaptor, MAPK And MTOR Activator 2	Protein Coding	39	GC01P156054	0.880828738
MBOAT7	Membrane Bound O-Acyltransferase Domain Containing 7	Protein Coding	39	GC19M054173	0.880828738
PCDH7	Protocadherin 7	Protein Coding	39	GC04P030722	0.880828738
RAB11FIP5	RAB11 Family Interacting Protein 5	Protein Coding	39	GC02M073128	0.880828738
RALGAPA1	Ral GTPase Activating Protein Catalytic Subunit Alpha 1	Protein Coding	39	GC14M035538	0.880828738
RPS6KC1	Ribosomal Protein S6 Kinase C1	Protein Coding	39	GC01P213051	0.880828738

RRAGC	Ras Related GTP Binding C	Protein Coding	39	GC01M038858	0.880828738
SAR1A	Secretion Associated Ras Related GTPase 1A	Protein Coding	39	GC10M070147	0.880828738
SEC24B	SEC24 Homolog B, COPII Coat Complex Component	Protein Coding	39	GC04P109433	0.880828738
SLC38A2	Solute Carrier Family 38 Member 2	Protein Coding	39	GC12M046358	0.880828738
SNX1	Sorting Nexin 1	Protein Coding	39	GC15P064094	0.880828738
STX5	Syntaxin 5	Protein Coding	39	GC11M062806	0.880828738
VPS13B	Vacuolar Protein Sorting 13 Homolog B	Protein Coding	39	GC08P099011	0.880828738
VPS41	VPS41 Subunit Of HOPS Complex	Protein Coding	39	GC07M039082	0.880828738
ACBD5	Acyl-CoA Binding Domain Containing 5	Protein Coding	38	GC10M027182	0.880828738
ADGRL2	Adhesion G Protein-Coupled Receptor L2	Protein Coding	38	GC01P081306	0.880828738
APBB2	Amyloid Beta Precursor Protein Binding Family B Member 2	Protein Coding	38	GC04M040812	0.880828738
BICD1	BICD Cargo Adaptor 1	Protein Coding	38	GC12P032107	0.880828738
CDKAL1	CDK5 Regulatory Subunit Associated Protein 1 Like 1	Protein Coding	38	GC06P020534	0.880828738
CLCC1	Chloride Channel CLIC Like 1	Protein Coding	38	GC01M108881	0.880828738
COG6	Component Of Oligomeric Golgi Complex 6	Protein Coding	38	GC13P039655	0.880828738
CTDSP2	CTD Small Phosphatase 2	Protein Coding	38	GC12M057819	0.880828738
DCTN4	Dynactin Subunit 4	Protein Coding	38	GC05M150708	0.880828738
DNAJC13	DnaJ Heat Shock Protein Family (Hsp40) Member C13	Protein Coding	38	GC03P132417	0.880828738
FMN2	Formin 2	Protein Coding	38	GC01P240014	0.880828738
GIGYF2	GRB10 Interacting GYF Protein 2	Protein Coding	38	GC02P232698	0.880828738
GORASP2	Golgi Reassembly Stacking Protein 2	Protein Coding	38	GC02P170928	0.880828738
JPH1	Junctophilin 1	Protein Coding	38	GC08M074234	0.880828738
KDELR1	KDEL Endoplasmic Reticulum Protein Retention Receptor 1	Protein Coding	38	GC19M048382	0.880828738
LAMTOR3	Late Endosomal/Lysosomal Adaptor, MAPK And MTOR Activator 3	Protein Coding	38	GC04M099878	0.880828738
LYST	Lysosomal Trafficking Regulator	Protein Coding	38	GC01M235661	0.880828738
MAPK15	Mitogen-Activated Protein Kinase 15	Protein Coding	38	GC08P143716	0.880828738
MARVELD2	MARVEL Domain Containing 2	Protein Coding	38	GC05P069415	0.880828738
MTDH	Metadherin	Protein Coding	38	GC08P097644	0.880828738
PEG3	Paternally Expressed 3	Protein Coding	38	GC19M056810	0.880828738
PGRMC2	Progesterone Receptor Membrane Component 2	Protein Coding	38	GC04M128269	0.880828738
PIGS	Phosphatidylinositol Glycan Anchor Biosynthesis Class S	Protein Coding	38	GC17M028553	0.880828738
PREB	Prolactin Regulatory Element Binding	Protein Coding	38	GC02M027130	0.880828738
RRBP1	Ribosome Binding Protein 1	Protein Coding	38	GC20M017613	0.880828738
SCAMP1	Secretory Carrier Membrane Protein 1	Protein Coding	38	GC05P078360	0.880828738
SCFD1	Sec1 Family Domain Containing 1	Protein Coding	38	GC14P030622	0.880828738
SLC30A5	Solute Carrier Family 30 Member 5	Protein Coding	38	GC05P069093	0.880828738
SLC39A10	Solute Carrier Family 39 Member 10	Protein Coding	38	GC02P195575	0.880828738
SNX2	Sorting Nexin 2	Protein Coding	38	GC05P122774	0.880828738
SPG11	SPG11 Vesicle Trafficking Associated, Spatacsin	Protein Coding	38	GC15M044562	0.880828738
STARD3	StAR Related Lipid Transfer Domain Containing 3	Protein Coding	38	GC17P039637	0.880828738
STIM2	Stromal Interaction Molecule 2	Protein Coding	38	GC04P026859	0.880828738
STX6	Syntaxin 6	Protein Coding	38	GC01M180972	0.880828738
STX7	Syntaxin 7	Protein Coding	38	GC06M132445	0.880828738
SUN1	Sad1 And UNC84 Domain Containing 1	Protein Coding	38	GC07P000815	0.880828738
SUN2	Sad1 And UNC84 Domain Containing 2	Protein Coding	38	GC22M057589	0.880828738
SYNE2	Spectrin Repeat Containing Nuclear Envelope Protein 2	Protein Coding	38	GC14P063761	0.880828738
TMCO1	Transmembrane And Coiled-Coil Domains 1	Protein Coding	38	GC01M165724	0.880828738
TMEM106B	Transmembrane Protein 106B	Protein Coding	38	GC07P012228	0.880828738
TMOD3	Tropomodulin 3	Protein Coding	38	GC15P051829	0.880828738
UBE2E2	Ubiquitin Conjugating Enzyme E2 E2	Protein Coding	38	GC03P023221	0.880828738
USP32	Ubiquitin Specific Peptidase 32	Protein Coding	38	GC17M060267	0.880828738
VPS26A	VPS26 Retromer Complex Component A	Protein Coding	38	GC10P069123	0.880828738
VPS45	Vacuolar Protein Sorting 45 Homolog	Protein Coding	38	GC01P150252	0.880828738
YKT6	YKT6 V-SNARE Homolog	Protein Coding	38	GC07P044200	0.880828738
ZC3HAV1	Zinc Finger CCCH-Type Containing, Antiviral 1	Protein Coding	38	GC07M139057	0.880828738
ZW10	Zw10 Kinetochore Protein	Protein Coding	38	GC11M113733	0.880828738
ACBD3	Acyl-CoA Binding Domain Containing 3	Protein Coding	37	GC01M226144	0.880828738
AKAP11	A-Kinase Anchoring Protein 11	Protein Coding	37	GC13P042272	0.880828738
ANKS6	Ankyrin Repeat And Sterile Alpha Motif Domain Containing 6	Protein Coding	37	GC09M098731	0.880828738
ARFGEF1	ADP Ribosylation Factor Guanine Nucleotide Exchange Factor 1	Protein Coding	37	GC08M067173	0.880828738
ATF6B	Activating Transcription Factor 6 Beta	Protein Coding	37	GC06M032115	0.880828738
ATP9A	ATPase Phospholipid Transporting 9A (Putative)	Protein Coding	37	GC20M051596	0.880828738
CDCA3	Cell Division Cycle Associated 3	Protein Coding	37	GC12M006844	0.880828738
CDK5RAP1	CDK5 Regulatory Subunit Associated Protein 1	Protein Coding	37	GC20M033358	0.880828738
CDK5RAP3	CDK5 Regulatory Subunit Associated Protein 3	Protein Coding	37	GC17P047967	0.880828738
CHML	CHM Like Rab Escort Protein	Protein Coding	37	GC01M241628	0.880828738
DDX54	DEAD-Box Helicase 54	Protein Coding	37	GC12M113157	0.880828738
DHX29	DExH-Box Helicase 29	Protein Coding	37	GC05M055256	0.880828738
FAF2	Fas Associated Factor Family Member 2	Protein Coding	37	GC05P176447	0.880828738
FNIP1	Folliculin Interacting Protein 1	Protein Coding	37	GC05M131641	0.880828738
GOLGA5	Golgin A5	Protein Coding	37	GC14P092794	0.880828738
KIAA0319L	KIAA0319 Like	Protein Coding	37	GC01M035433	0.880828738
LRIG3	Leucine Rich Repeats And Immunoglobulin Like Domains 3	Protein Coding	37	GC12M058872	0.880828738
NETO2	Neuropilin And Tolloid Like 2	Protein Coding	37	GC16M047077	0.880828738
OSBPL1A	Oxysterol Binding Protein Like 1A	Protein Coding	37	GC18M024162	0.880828738
OSBPL8	Oxysterol Binding Protein Like 8	Protein Coding	37	GC12M076354	0.880828738
OSBPL9	Oxysterol Binding Protein Like 9	Protein Coding	37	GC01P051577	0.880828738
PPP1R15B	Protein Phosphatase 1 Regulatory Subunit 15B	Protein Coding	37	GC01M204509	0.880828738
RASAL2	RAS Protein Activator Like 2	Protein Coding	37	GC01P178093	0.880828738
RETSAT	Retinol Saturase	Protein Coding	37	GC02M085344	0.880828738
RINT1	RAD50 Interactor 1	Protein Coding	37	GC07P105532	0.880828738
RRAGA	Ras Related GTP Binding A	Protein Coding	37	GC09P019049	0.880828738
RTN1	Reticulon 1	Protein Coding	37	GC14M059595	0.880828738
SCAMP3	Secretory Carrier Membrane Protein 3	Protein Coding	37	GC01M155255	0.880828738
SCYL3	SCY1 Like Pseudokinase 3	Protein Coding	37	GC01M169821	0.880828738
SEC24A	SEC24 Homolog A, COPII Coat Complex Component	Protein Coding	37	GC05P134647	0.880828738
SEMA4C	Semaphorin 4C	Protein Coding	37	GC02M096859	0.880828738
SLC30A6	Solute Carrier Family 30 Member 6	Protein Coding	37	GC02P032166	0.880828738
SLC30A7	Solute Carrier Family 30 Member 7	Protein Coding	37	GC01P100896	0.880828738
SNAPIN	SNAP Associated Protein	Protein Coding	37	GC01P153660	0.880828738
SNX14	Sorting Nexin 14	Protein Coding	37	GC06M085505	0.880828738
SRPRB	SRP Receptor Subunit Beta	Protein Coding	37	GC03P133784	0.880828738
STXBP5	Syntaxin Binding Protein 5	Protein Coding	37	GC06P147204	0.880828738
SYNGR2	Synaptogyrin 2	Protein Coding	37	GC17P078168	0.880828738
TMX1	Thioredoxin Related Transmembrane Protein 1	Protein Coding	37	GC14P051240	0.880828738
TRAPPC2	Trafficking Protein Particle Complex Subunit 2	Protein Coding	37	GC0XM013712	0.880828738
TYW1	TRNA-YW Synthesizing Protein 1 Homolog	Protein Coding	37	GC07P066995	0.880828738
UBAP2L	Ubiquitin Associated Protein 2 Like	Protein Coding	37	GC01P154219	0.880828738

VIPAS39	VPS33B Interacting Protein, Apical-Basolateral Polarity Regulator, Spe-39 Homolog	Protein Coding	37	GC14M077426	0.880828738
VKORC1L1	Vitamin K Epoxide Reductase Complex Subunit 1 Like 1	Protein Coding	37	GC07P070500	0.880828738
ZDHHC13	Zinc Finger DHHC-Type Palmitoyltransferase 13	Protein Coding	37	GC11P019095	0.880828738
ACTR10	Actin Related Protein 10	Protein Coding	36	GC14P058200	0.880828738
ANKRD27	Ankyrin Repeat Domain 27	Protein Coding	36	GC19M032597	0.880828738
ARFIP1	ADP Ribosylation Factor Interacting Protein 1	Protein Coding	36	GC04P152779	0.880828738
ARL8B	ADP Ribosylation Factor Like GTPase 8B	Protein Coding	36	GC03P005122	0.880828738
AUP1	AUP1 Lipid Droplet Regulating VLDL Assembly Factor	Protein Coding	36	GC02M074526	0.880828738
CHP1	Calcineurin Like EF-Hand Protein 1	Protein Coding	36	GC15P041230	0.880828738
COG3	Component Of Oligomeric Golgi Complex 3	Protein Coding	36	GC13P045464	0.880828738
DENND4A	DENN Domain Containing 4A	Protein Coding	36	GC15M065658	0.880828738
DMXL2	Dmx Like 2	Protein Coding	36	GC15M051447	0.880828738
DNAJC1	DnaJ Heat Shock Protein Family (Hsp40) Member C1	Protein Coding	36	GC10M021762	0.880828738
FNIP2	Folliculin Interacting Protein 2	Protein Coding	36	GC04P158769	0.880828738
GOLGA3	Golgin A3	Protein Coding	36	GC12M132768	0.880828738
GOLGA4	Golgin A4	Protein Coding	36	GC03P037243	0.880828738
GOLGB1	Golgin B1	Protein Coding	36	GC03M121663	0.880828738
GRIPAP1	GRIP1 Associated Protein 1	Protein Coding	36	GC0XM048973	0.880828738
HS1BP3	HCLS1 Binding Protein 3	Protein Coding	36	GC02M020560	0.880828738
KIF16B	Kinesin Family Member 16B	Protein Coding	36	GC20M016272	0.880828738
LACTB2	Lactamase Beta 2	Protein Coding	36	GC08M070635	0.880828738
MEGF8	Multiple EGF Like Domains 8	Protein Coding	36	GC19P042325	0.880828738
MIOS	Meiosis Regulator For Oocyte Development	Protein Coding	36	GC07P007580	0.880828738
NUP35	Nucleoporin 35	Protein Coding	36	GC02P183117	0.880828738
OSBPL11	Oxysterol Binding Protein Like 11	Protein Coding	36	GC03M125529	0.880828738
PDXDC1	Pyridoxal Dependent Decarboxylase Domain Containing 1	Protein Coding	36	GC16P014974	0.880828738
PIGU	Phosphatidylinositol Glycan Anchor Biosynthesis Class U	Protein Coding	36	GC20M034560	0.880828738
RABGAP1	RAB GTPase Activating Protein 1	Protein Coding	36	GC09P122932	0.880828738
RBSN	Rabenosyn, RAB Effector	Protein Coding	36	GC03M015070	0.880828738
RUFY2	RUN And FYVE Domain Containing 2	Protein Coding	36	GC10M068341	0.880828738
SCIN	Scinderin	Protein Coding	36	GC07P012570	0.880828738
SEC16A	SEC16 Homolog A, Endoplasmic Reticulum Export Factor	Protein Coding	36	GC09M136440	0.880828738
SGTA	Small Glutamine Rich Tetratricopeptide Repeat Co-Chaperone Alpha	Protein Coding	36	GC19M002754	0.880828738
ST7	Suppression Of Tumorigenicity 7	Protein Coding	36	GC07P117083	0.880828738
STX12	Syntaxin 12	Protein Coding	36	GC01P027797	0.880828738
STX18	Syntaxin 18	Protein Coding	36	GC04M004417	0.880828738
SYNJ2BP	Synaptojanin 2 Binding Protein	Protein Coding	36	GC14M070366	0.880828738
TAPT1	Transmembrane Anterior Posterior Transformation 1	Protein Coding	36	GC04M016162	0.880828738
TENM1	Teneurin Transmembrane Protein 1	Protein Coding	36	GC0XM124375	0.880828738
THAP11	THAP Domain Containing 11	Protein Coding	36	GC16P067844	0.880828738
TMF1	TATA Element Modulatory Factor 1	Protein Coding	36	GC03M069019	0.880828738
TRIM13	Tripartite Motif Containing 13	Protein Coding	36	GC13P049995	0.880828738
UBXN4	UBX Domain Protein 4	Protein Coding	36	GC02P135741	0.880828738
VMA21	Vacuolar ATPase Assembly Factor VMA21	Protein Coding	36	GC0XP151396	0.880828738
VPS8	VPS8 Subunit Of CORVET Complex	Protein Coding	36	GC03P184812	0.880828738
WDR59	WD Repeat Domain 59	Protein Coding	36	GC16M074871	0.880828738
WDR81	WD Repeat Domain 81	Protein Coding	36	GC17P001716	0.880828738
ZFYVE27	Zinc Finger FYVE-Type Containing 27	Protein Coding	36	GC10P097739	0.880828738
B9D1	B9 Domain Containing 1	Protein Coding	35	GC17M019334	0.880828738
BET1L	Bet1 Golgi Vesicular Membrane Trafficking Protein Like	Protein Coding	35	GC11M003166	0.880828738
BNIP1	BCL2 Interacting Protein 1	Protein Coding	35	GC05P173144	0.880828738
CPNE8	Copine 8	Protein Coding	35	GC12M038646	0.880828738
DHRS7	Dehydrogenase/Reductase 7	Protein Coding	35	GC14M060144	0.880828738
DHX57	DExH-Box Helicase 57	Protein Coding	35	GC02M038797	0.880828738
ESYT2	Extended Synaptotagmin 2	Protein Coding	35	GC07M158730	0.880828738
GCC1	GRIP And Coiled-Coil Domain Containing 1	Protein Coding	35	GC07M127580	0.880828738
NECTIN2	Nectin Cell Adhesion Molecule 2	Protein Coding	35	GC19P066877	0.880828738
PARP16	Poly(ADP-Ribose) Polymerase Family Member 16	Protein Coding	35	GC15M089975	0.880828738
RHBDD2	Rhomboid Domain Containing 2	Protein Coding	35	GC07P075842	0.880828738
SNX17	Sorting Nexin 17	Protein Coding	35	GC02P027370	0.880828738
SNX19	Sorting Nexin 19	Protein Coding	35	GC11M130868	0.880828738
USE1	Unconventional SNARE In The ER 1	Protein Coding	35	GC19P066217	0.880828738
VMP1	Vacuole Membrane Protein 1	Protein Coding	35	GC17P059707	0.880828738
VPS18	VPS18 Core Subunit Of CORVET And HOPS Complexes	Protein Coding	35	GC15P040894	0.880828738
YIPF3	Yip1 Domain Family Member 3	Protein Coding	35	GC06M066209	0.880828738
ANGEL1	Angel Homolog 1	Protein Coding	34	GC14M077059	0.880828738
ANKRD50	Ankyrin Repeat Domain Containing 50	Protein Coding	34	GC04M124664	0.880828738
APPL2	Adaptor Protein, Phosphotyrosine Interacting With PH Domain And Leucine Zipper 2	Protein Coding	34	GC12M105173	0.880828738
CCDC47	Coiled-Coil Domain Containing 47	Protein Coding	34	GC17M063745	0.880828738
DEF8	Differentially Expressed In FDCP 8 Homolog	Protein Coding	34	GC16P091335	0.880828738
FAM83B	Family With Sequence Similarity 83 Member B	Protein Coding	34	GC06P054846	0.880828738
FKBP15	FKBP Prolyl Isomerase Family Member 15	Protein Coding	34	GC09M113161	0.880828738
FNDC3A	Fibronectin Type III Domain Containing 3A	Protein Coding	34	GC13P048975	0.880828738
GPRIN1	G Protein Regulated Inducer Of Neurite Outgrowth 1	Protein Coding	34	GC05M176595	0.880828738
HEATR5B	HEAT Repeat Containing 5B	Protein Coding	34	GC02M036949	0.880828738
KXD1	KxDL Motif Containing 1	Protein Coding	34	GC19P018557	0.880828738
LRCH4	Leucine Rich Repeats And Calponin Homology Domain Containing 4	Protein Coding	34	GC07M100574	0.880828738
MOSPD2	Motile Sperm Domain Containing 2	Protein Coding	34	GC0XP014891	0.880828738
PDZD8	PDZ Domain Containing 8	Protein Coding	34	GC10M117277	0.880828738
PLEKHM2	Pleckstrin Homology And RUN Domain Containing M2	Protein Coding	34	GC01P015826	0.880828738
RABAC1	Rab Acceptor 1	Protein Coding	34	GC19M041956	0.880828738
RABL3	RAB, Member Of RAS Oncogene Family Like 3	Protein Coding	34	GC03M120686	0.880828738
RUFY3	RUN And FYVE Domain Containing 3	Protein Coding	34	GC04P070716	0.880828738
SNX11	Sorting Nexin 11	Protein Coding	34	GC17P048103	0.880828738
SNX4	Sorting Nexin 4	Protein Coding	34	GC03M125446	0.880828738
STBD1	Starch Binding Domain 1	Protein Coding	34	GC04P076306	0.880828738
STK11IP	Serine/Threonine Kinase 11 Interacting Protein	Protein Coding	34	GC02P219597	0.880828738
TBC1D22A	TBC1 Domain Family Member 22A	Protein Coding	34	GC22P046762	0.880828738
TMED9	Transmembrane P24 Trafficking Protein 9	Protein Coding	34	GC05P177594	0.880828738
TMEM161A	Transmembrane Protein 161A	Protein Coding	34	GC19M019120	0.880828738
TMEM87A	Transmembrane Protein 87A	Protein Coding	34	GC15M042210	0.880828738
VEZT	Vezatin, Adherens Junctions Transmembrane Protein	Protein Coding	34	GC12P095217	0.880828738
VPS26B	VPS26 Retromer Complex Component B	Protein Coding	34	GC11P134224	0.880828738
WDR41	WD Repeat Domain 41	Protein Coding	34	GC05M077425	0.880828738
ANKRD46	Ankyrin Repeat Domain 46	Protein Coding	33	GC08M100510	0.880828738
ARMC10	Armadillo Repeat Containing 10	Protein Coding	33	GC07P103074	0.880828738
ARMCX2	Armadillo Repeat Containing X-Linked 2	Protein Coding	33	GC0XM101658	0.880828738
BLOC1S2	Biogenesis Of Lysosomal Organelles Complex 1 Subunit 2	Protein Coding	33	GC10M100273	0.880828738

DMXL1	Dmx Like 1	Protein Coding	33	GC05P119037	0.880828738
FAM135A	Family With Sequence Similarity 135 Member A	Protein Coding	33	GC06P070412	0.880828738
FAM171A1	Family With Sequence Similarity 171 Member A1	Protein Coding	33	GC10M015254	0.880828738
GRAMD1A	GRAM Domain Containing 1A	Protein Coding	33	GC19P066531	0.880828738
KHNYN	KH And NYN Domain Containing	Protein Coding	33	GC14P032725	0.880828738
LAMTOR1	Late Endosomal/Lysosomal Adaptor, MAPK And MTOR Activator 1	Protein Coding	33	GC11M072085	0.880828738
LRCH3	Leucine Rich Repeats And Calponin Homology Domain Containing 3	Protein Coding	33	GC03P197791	0.880828738
MYO19	Myosin XIX	Protein Coding	33	GC17M036495	0.880828738
NDC1	NDC1 Transmembrane Nucleoporin	Protein Coding	33	GC01M053765	0.880828738
OSBPL10	Oxysterol Binding Protein Like 10	Protein Coding	33	GC03M031677	0.880828738
PLEKHM3	Pleckstrin Homology Domain Containing M3	Protein Coding	33	GC02M207822	0.880828738
PRR11	Proline Rich 11	Protein Coding	33	GC17P059155	0.880828738
RGP1	RGP1 Homolog, RAB6A GEF Complex Partner 1	Protein Coding	33	GC09P035749	0.880828738
RHBDD3	Rhomboid Domain Containing 3	Protein Coding	33	GC22M029259	0.880828738
RNF103	Ring Finger Protein 103	Protein Coding	33	GC02M086603	0.880828738
SCFD2	Sec1 Family Domain Containing 2	Protein Coding	33	GC04M052872	0.880828738
SEH1L	SEH1 Like Nucleoporin	Protein Coding	33	GC18P012947	0.880828738
SLC35E1	Solute Carrier Family 35 Member E1	Protein Coding	33	GC19M016549	0.880828738
SNX29	Sorting Nexin 29	Protein Coding	33	GC16P011976	0.880828738
TEX2	Testis Expressed 2	Protein Coding	33	GC17M064147	0.880828738
TEX264	Testis Expressed 264, ER-Phagy Receptor	Protein Coding	33	GC03P051663	0.880828738
TMEM131	Transmembrane Protein 131	Protein Coding	33	GC02M098304	0.880828738
TMEM214	Transmembrane Protein 214	Protein Coding	33	GC02P027032	0.880828738
TOR1AIP2	Torsin 1A Interacting Protein 2	Protein Coding	33	GC01M183565	0.880828738
TRABD	TraB Domain Containing	Protein Coding	33	GC22P050185	0.880828738
WDR7	WD Repeat Domain 7	Protein Coding	33	GC18P056651	0.880828738
COA7	Cytochrome C Oxidase Assembly Factor 7	Protein Coding	32	GC01M052684	0.880828738
DENND4C	DENN Domain Containing 4C	Protein Coding	32	GC09P019230	0.880828738
EMC3	ER Membrane Protein Complex Subunit 3	Protein Coding	32	GC03M010424	0.880828738
EMC4	ER Membrane Protein Complex Subunit 4	Protein Coding	32	GC15P042292	0.880828738
EMC8	ER Membrane Protein Complex Subunit 8	Protein Coding	32	GC16M085771	0.880828738
EXD2	Exonuclease 3'-5' Domain Containing 2	Protein Coding	32	GC14P069191	0.880828738
GPAT3	Glycerol-3-Phosphate Acyltransferase 3	Protein Coding	32	GC04P083536	0.880828738
GPR89A	G Protein-Coupled Receptor 89A	Protein Coding	32	GC01P145607	0.880828738
MXRA7	Matrix Remodeling Associated 7	Protein Coding	32	GC17M076672	0.880828738
RAB29	RAB29, Member RAS Oncogene Family	Protein Coding	32	GC01M205768	0.880828738
RUNDC3B	RUN Domain Containing 3B	Protein Coding	32	GC07P087627	0.880828738
SNAP47	Synaptosome Associated Protein 47	Protein Coding	32	GC01P227730	0.880828738
SNX30	Sorting Nexin Family Member 30	Protein Coding	32	GC09P112753	0.880828738
TMEM106C	Transmembrane Protein 106C	Protein Coding	32	GC12P047963	0.880828738
TMEM201	Transmembrane Protein 201	Protein Coding	32	GC01P009588	0.880828738
TMEM209	Transmembrane Protein 209	Protein Coding	32	GC07M130164	0.880828738
UBXN8	UBX Domain Protein 8	Protein Coding	32	GC08P030723	0.880828738
WDR91	WD Repeat Domain 91	Protein Coding	32	GC07M135183	0.880828738
DNAJC16	DnaJ Heat Shock Protein Family (Hsp40) Member C16	Protein Coding	31	GC01P015526	0.880828738
EMC2	ER Membrane Protein Complex Subunit 2	Protein Coding	31	GC08P108443	0.880828738
RIC1	RIC1 Homolog, RAB6A GEF Complex Partner 1	Protein Coding	31	GC09P006538	0.880828738
ARFGEF3	ARFGEF Family Member 3	Protein Coding	30	GC06P138161	0.880828738
C19orf25	Chromosome 19 Open Reading Frame 25	Protein Coding	30	GC19M005285	0.880828738
EMC7	ER Membrane Protein Complex Subunit 7	Protein Coding	30	GC15M034084	0.880828738
GPAT4	Glycerol-3-Phosphate Acyltransferase 4	Protein Coding	30	GC08P041577	0.880828738
SMCR8	SMCR8-C9orf72 Complex Subunit	Protein Coding	30	GC17P054654	0.880828738
SRPRA	SRP Receptor Subunit Alpha	Protein Coding	30	GC11M126255	0.880828738
TMED8	Transmembrane P24 Trafficking Protein Family Member 8	Protein Coding	30	GC14M077335	0.880828738
WASHC5	WASH Complex Subunit 5	Protein Coding	30	GC08M132157	0.880828738
CEMIP2	Cell Migration Inducing Hyaluronidase 2	Protein Coding	29	GC09M071684	0.880828738
LAMTOR4	Late Endosomal/Lysosomal Adaptor, MAPK And MTOR Activator 4	Protein Coding	29	GC07P100148	0.880828738
NUP42	Nucleoporin 42	Protein Coding	29	GC07P023182	0.880828738
TYW1B	TRNA-YW Synthesizing Protein 1 Homolog B	Protein Coding	29	GC07M072559	0.880828738
MACO1	Macoilin 1	Protein Coding	28	GC01P025430	0.880828738
VPS35L	VPS35 Endosomal Protein Sorting Factor Like	Protein Coding	27	GC16P019555	0.880828738
WASHC4	WASH Complex Subunit 4	Protein Coding	27	GC12P105108	0.880828738
BLTP3A	Bridge-Like Lipid Transfer Protein Family Member 3A	Protein Coding	26	GC06P084361	0.880828738
BORCS5	BLOC-1 Related Complex Subunit 5	Protein Coding	26	GC12P021313	0.880828738
BORCS6	BLOC-1 Related Complex Subunit 6	Protein Coding	26	GC17M010441	0.880828738
DOP1B	DOP1 Leucine Zipper Like Protein B	Protein Coding	26	GC21P036170	0.880828738
LNPK	Lunapark, ER Junction Formation Factor	Protein Coding	26	GC02M175924	0.880828738
VPS26C	VPS26 Endosomal Protein Sorting Factor C	Protein Coding	26	GC21M037225	0.880828738
TMEM131L	Transmembrane 131 Like	Protein Coding	25	GC04P153467	0.880828738
WASHC2C	WASH Complex Subunit 2C	Protein Coding	25	GC10P045761	0.880828738
WASHC3	WASH Complex Subunit 3	Protein Coding	25	GC12M102013	0.880828738
MIGA1	Mitoguardin 1	Protein Coding	24	GC01P077844	0.880828738
NEAT1	Nuclear Paraspeckle Assembly Transcript 1	RNA Gene	24	GC11P069943	0.880828738
WASHC1	WASH Complex Subunit 1	Protein Coding	23	GC09M000016	0.880828738
MIR150	MicroRNA 150	RNA Gene	22	GC19M049500	0.880828738
WASHC2A	WASH Complex Subunit 2A	Protein Coding	22	GC10P050213	0.880828738
IGHV3-23	Immunoglobulin Heavy Variable 3-23	Protein Coding	20	GC14M106268	0.880828738
MIR137	MicroRNA 137	RNA Gene	20	GC01M098046	0.880828738
IGH	Immunoglobulin Heavy Locus	Protein Coding	18	GC14M112706	0.880828738
AR	Androgen Receptor	Protein Coding	50	GC0XP067544	0.849134505
PPP3CA	Protein Phosphatase 3 Catalytic Subunit Alpha	Protein Coding	50	GC04M101024	0.849134505
ALDH2	Aldehyde Dehydrogenase 2 Family Member	Protein Coding	49	GC12P111766	0.849134505
CTSK	Cathepsin K	Protein Coding	49	GC01M152112	0.849134505
HSPB1	Heat Shock Protein Family B (Small) Member 1	Protein Coding	49	GC07P076302	0.849134505
NFKB1	Nuclear Factor Kappa B Subunit 1	Protein Coding	49	GC04P102501	0.849134505
PRKACA	Protein Kinase CAMP-Activated Catalytic Subunit Alpha	Protein Coding	49	GC19M014497	0.849134505
MAPT	Microtubule Associated Protein Tau	Protein Coding	48	GC17P045894	0.849134505
FANCA	FA Complementation Group A	Protein Coding	47	GC16M089727	0.849134505
PGK1	Phosphoglycerate Kinase 1	Protein Coding	47	GC0XP077994	0.849134505
CCL2	C-C Motif Chemokine Ligand 2	Protein Coding	46	GC17P034255	0.849134505
GLUL	Glutamate-Ammonia Ligase	Protein Coding	46	GC01M182378	0.849134505
GSK3A	Glycogen Synthase Kinase 3 Alpha	Protein Coding	46	GC19M065831	0.849134505
SIRT2	Sirtuin 2	Protein Coding	46	GC19M038878	0.849134505
ENO2	Enolase 2	Protein Coding	45	GC12P006913	0.849134505
FZD5	Frizzled Class Receptor 5	Protein Coding	45	GC02M207762	0.849134505
GFAP	Glial Fibrillary Acidic Protein	Protein Coding	45	GC17M044917	0.849134505
GOT2	Glutamic-Oxaloacetic Transaminase 2	Protein Coding	45	GC16M058707	0.849134505
KAT2A	Lysine Acetyltransferase 2A	Protein Coding	45	GC17M042113	0.849134505

PDHA1	Pyruvate Dehydrogenase E1 Subunit Alpha 1	Protein Coding	45	GC0XP019343	0.849134505
ISG15	ISG15 Ubiquitin Like Modifier	Protein Coding	44	GC01P001001	0.849134505
SREBF1	Sterol Regulatory Element Binding Transcription Factor 1	Protein Coding	44	GC17M017810	0.849134505
TDP1	Tyrosyl-DNA Phosphodiesterase 1	Protein Coding	44	GC14P089954	0.849134505
TUBA8	Tubulin Alpha 8	Protein Coding	44	GC22P018110	0.849134505
UBE2D3	Ubiquitin Conjugating Enzyme E2 D3	Protein Coding	44	GC04M102794	0.849134505
CKB	Creatine Kinase B	Protein Coding	43	GC14M103519	0.849134505
PSMB10	Proteasome 20S Subunit Beta 10	Protein Coding	43	GC16M067937	0.849134505
STK3	Serine/Threonine Kinase 3	Protein Coding	43	GC08M098343	0.849134505
CNP	2',3'-Cyclic Nucleotide 3' Phosphodiesterase	Protein Coding	42	GC17P041966	0.849134505
CORO1A	Coronin 1A	Protein Coding	42	GC16P041041	0.849134505
E2F1	E2F Transcription Factor 1	Protein Coding	42	GC20M033675	0.849134505
NPEPPS	Aminopeptidase Puromycin Sensitive	Protein Coding	42	GC17P047522	0.849134505
POLR3A	RNA Polymerase III Subunit A	Protein Coding	42	GC10M078577	0.849134505
U2AF1	U2 Small Nuclear RNA Auxiliary Factor 1	Protein Coding	42	GC21M043092	0.849134505
ATP1B2	ATPase Na+/K+ Transporting Subunit Beta 2	Protein Coding	41	GC17P011175	0.849134505
GPM6A	Glycoprotein M6A	Protein Coding	41	GC04M175632	0.849134505
UBE2G1	Ubiquitin Conjugating Enzyme E2 G1	Protein Coding	41	GC17M004569	0.849134505
ACTL6A	Actin Like 6A	Protein Coding	40	GC03P179562	0.849134505
CLN5	CLN5 Intracellular Trafficking Protein	Protein Coding	40	GC13P076990	0.849134505
ZBTB17	Zinc Finger And BTB Domain Containing 17	Protein Coding	40	GC01M015943	0.849134505
UBD	Ubiquitin D	Protein Coding	38	GC06M065827	0.849134505
UBE2Z	Ubiquitin Conjugating Enzyme E2 Z	Protein Coding	38	GC17P048908	0.849134505
ZDHHC8	Zinc Finger DHHC-Type Palmitoyltransferase 8	Protein Coding	38	GC22P020129	0.849134505
SLC17A9	Solute Carrier Family 17 Member 9	Protein Coding	37	GC20P062952	0.849134505
U2AF2	U2 Small Nuclear RNA Auxiliary Factor 2	Protein Coding	36	GC19P055654	0.849134505
WBP11	WW Domain Binding Protein 11	Protein Coding	36	GC12M014784	0.849134505
LRRC17	Leucine Rich Repeat Containing 17	Protein Coding	33	GC07P102992	0.849134505
NAXE	NAD(P)HX Epimerase	Protein Coding	33	GC01P156591	0.849134505
WDR75	WD Repeat Domain 75	Protein Coding	33	GC02P189441	0.849134505
DRAM1	DNA Damage Regulated Autophagy Modulator 1	Protein Coding	30	GC12P101877	0.849134505
EMC6	ER Membrane Protein Complex Subunit 6	Protein Coding	30	GC17P003668	0.849134505
KRT6A	Keratin 6A	Protein Coding	42	GC12M052488	0.836693645
MAPK8	Mitogen-Activated Protein Kinase 8	Protein Coding	47	GC10P048306	0.822651505
MAPK11	Mitogen-Activated Protein Kinase 11	Protein Coding	45	GC22M050263	0.822651505
MAP2K5	Mitogen-Activated Protein Kinase Kinase 5	Protein Coding	44	GC15P117541	0.822651505
MAP2K7	Mitogen-Activated Protein Kinase Kinase 7	Protein Coding	44	GC19P007903	0.822651505
NAA10	N-Alpha-Acetyltransferase 10, NatA Catalytic Subunit	Protein Coding	41	GC0XM153929	0.822651505
PPM1K	Protein Phosphatase, Mg2+/Mn2+ Dependent 1K	Protein Coding	41	GC04M088258	0.822651505
DOCK3	Dedicator Of Cytokinesis 3	Protein Coding	40	GC03P050675	0.822651505
WSCD1	WSC Domain Containing 1	Protein Coding	34	GC17P006057	0.822651505
NAA16	N-Alpha-Acetyltransferase 16, NatA Auxiliary Subunit	Protein Coding	32	GC13P041311	0.822651505
MDM2	MDM2 Proto-Oncogene	Protein Coding	51	GC12P068808	0.807829857
LEPR	Leptin Receptor	Protein Coding	48	GC01P065421	0.807829857
PSMB7	Proteasome 20S Subunit Beta 7	Protein Coding	44	GC09M124353	0.807829857
UBE2A	Ubiquitin Conjugating Enzyme E2 A	Protein Coding	43	GC0XP119618	0.807829857
CYB5R1	Cytochrome B5 Reductase 1	Protein Coding	40	GC01M202964	0.807829857
UBE2J2	Ubiquitin Conjugating Enzyme E2 J2	Protein Coding	39	GC01M005950	0.807829857
PSMD8	Proteasome 26S Subunit, Non-ATPase 8	Protein Coding	38	GC19P038374	0.807829857
RNF34	Ring Finger Protein 34	Protein Coding	38	GC12P121400	0.807829857
TRIM31	Tripartite Motif Containing 31	Protein Coding	37	GC06M065862	0.807829857
SNPH	Syntaphilin	Protein Coding	34	GC20P001266	0.807829857
CLUH	Clustered Mitochondria Homolog	Protein Coding	32	GC17M002689	0.807829857
MIR423	MicroRNA 423	RNA Gene	19	GC17P030117	0.807829857
NME2	NME/NM23 Nucleoside Diphosphate Kinase 2	Protein Coding	45	GC17P051165	0.79584074
EIF2S1	Eukaryotic Translation Initiation Factor 2 Subunit Alpha	Protein Coding	42	GC14P067359	0.79584074
NEFM	Neurofilament Medium Chain	Protein Coding	41	GC08P024913	0.79584074
NUP93	Nucleoporin 93	Protein Coding	40	GC16P057060	0.79584074
ACIN1	Apoptotic Chromatin Condensation Inducer 1	Protein Coding	39	GC14M023058	0.79584074
CRNKL1	Crooked Neck Pre-MRNA Splicing Factor 1	Protein Coding	37	GC20M020034	0.79584074
TXLNA	Taxilin Alpha	Protein Coding	36	GC01P032179	0.79584074
TEAD4	TEA Domain Transcription Factor 4	Protein Coding	40	GC12P002959	0.772300422
MBD5	Methyl-CpG Binding Domain Protein 5	Protein Coding	37	GC02P148021	0.772300422
SLC35B3	Solute Carrier Family 35 Member B3	Protein Coding	35	GC06M008413	0.772300422
HSP90AA1	Heat Shock Protein 90 Alpha Family Class A Member 1	Protein Coding	46	GC14M102080	0.757606506
TCP1	T-Complex 1	Protein Coding	42	GC06M159778	0.757606506
PIK3CA	Phosphatidylinositol-4,5-Bisphosphate 3-Kinase Catalytic Subunit Alpha	Protein Coding	50	GC03P179148	0.745580971
MYLK	Myosin Light Chain Kinase	Protein Coding	49	GC03M123610	0.745580971
KCNK3	Potassium Two Pore Domain Channel Subfamily K Member 3	Protein Coding	48	GC02P026692	0.745580971
KCNQ1	Potassium Voltage-Gated Channel Subfamily Q Member 1	Protein Coding	48	GC11P002444	0.745580971
PRKG1	Protein Kinase CGMP-Dependent 1	Protein Coding	48	GC10P050991	0.745580971
SLC1A3	Solute Carrier Family 1 Member 3	Protein Coding	48	GC05P036646	0.745580971
ADRB2	Adrenoceptor Beta 2	Protein Coding	47	GC05P148825	0.745580971
GABRA5	Gamma-Aminobutyric Acid Type A Receptor Subunit Alpha5	Protein Coding	46	GC15P026866	0.745580971
SCN1A	Sodium Voltage-Gated Channel Alpha Subunit 1	Protein Coding	46	GC02M165989	0.745580971
C5	Complement C5	Protein Coding	45	GC09M120933	0.745580971
GDF5	Growth Differentiation Factor 5	Protein Coding	45	GC20M035433	0.745580971
MSTN	Myostatin	Protein Coding	45	GC02M190055	0.745580971
MYH11	Myosin Heavy Chain 11	Protein Coding	45	GC16M015705	0.745580971
PDK1	Pyruvate Dehydrogenase Kinase 1	Protein Coding	45	GC02P172555	0.745580971
STAT2	Signal Transducer And Activator Of Transcription 2	Protein Coding	45	GC12M056341	0.745580971
ANXA5	Annexin A5	Protein Coding	44	GC04M121667	0.745580971
FANCL	FA Complementation Group L	Protein Coding	44	GC02M058127	0.745580971
MAP3K12	Mitogen-Activated Protein Kinase Kinase Kinase 12	Protein Coding	44	GC12M053479	0.745580971
PFKP	Phosphofructokinase, Platelet	Protein Coding	44	GC10P003066	0.745580971
PLOD2	Procollagen-Lysine,2-Oxoglutarate 5-Dioxygenase 2	Protein Coding	44	GC03M146069	0.745580971
PNPO	Pyridoxamine 5'-Phosphate Oxidase	Protein Coding	44	GC17P047941	0.745580971
PRKD2	Protein Kinase D2	Protein Coding	44	GC19M046674	0.745580971
SLC1A4	Solute Carrier Family 1 Member 4	Protein Coding	44	GC02P064988	0.745580971
NDUFB9	NADH:Ubiquinone Oxidoreductase Subunit B9	Protein Coding	43	GC08P124539	0.745580971
NTHL1	Nth Like DNA Glycosylase 1	Protein Coding	43	GC16M007105	0.745580971
PDK4	Pyruvate Dehydrogenase Kinase 4	Protein Coding	43	GC07M095583	0.745580971
SLC22A3	Solute Carrier Family 22 Member 3	Protein Coding	43	GC06P160348	0.745580971
SMURF1	SMAD Specific E3 Ubiquitin Protein Ligase 1	Protein Coding	43	GC07M099027	0.745580971
CHST3	Carbohydrate Sulfotransferase 3	Protein Coding	42	GC10P071964	0.745580971
FABP1	Fatty Acid Binding Protein 1	Protein Coding	42	GC02M088122	0.745580971
FCGR3B	Fc Gamma Receptor IIIb	Protein Coding	42	GC01M161623	0.745580971
FGF14	Fibroblast Growth Factor 14	Protein Coding	42	GC13M101710	0.745580971

NR2C2	Nuclear Receptor Subfamily 2 Group C Member 2	Protein Coding	42	GC03P014947	0.745580971
SFRP4	Secreted Frizzled Related Protein 4	Protein Coding	42	GC07M037912	0.745580971
SLC25A19	Solute Carrier Family 25 Member 19	Protein Coding	42	GC17M075273	0.745580971
SLC37A4	Solute Carrier Family 37 Member 4	Protein Coding	42	GC11M119024	0.745580971
TNIK	TRAF2 And NCK Interacting Kinase	Protein Coding	42	GC03M171061	0.745580971
HAPLN1	Hyaluronan And Proteoglycan Link Protein 1	Protein Coding	41	GC05M083637	0.745580971
ITPKC	Inositol-Trisphosphate 3-Kinase C	Protein Coding	41	GC19P066710	0.745580971
PEX3	Peroxisomal Biogenesis Factor 3	Protein Coding	41	GC06P143450	0.745580971
ACKR3	Atypical Chemokine Receptor 3	Protein Coding	40	GC02P236537	0.745580971
ADAMTS7	ADAM Metallopeptidase With Thrombospondin Type 1 Motif 7	Protein Coding	40	GC15M078759	0.745580971
ALPK1	Alpha Kinase 1	Protein Coding	40	GC04P112285	0.745580971
CLDN7	Claudin 7	Protein Coding	40	GC17M007259	0.745580971
CPA3	Carboxypeptidase A3	Protein Coding	40	GC03P148865	0.745580971
FANCF	FA Complementation Group F	Protein Coding	40	GC11M022600	0.745580971
FGF7	Fibroblast Growth Factor 7	Protein Coding	40	GC15P049423	0.745580971
MAP1A	Microtubule Associated Protein 1A	Protein Coding	40	GC15P043550	0.745580971
REEP2	Receptor Accessory Protein 2	Protein Coding	40	GC05P138439	0.745580971
TREM1	Triggering Receptor Expressed On Myeloid Cells 1	Protein Coding	40	GC06M041267	0.745580971
BOC	BOC Cell Adhesion Associated, Oncogene Regulated	Protein Coding	39	GC03P113211	0.745580971
CD93	CD93 Molecule	Protein Coding	39	GC20M023079	0.745580971
CHAF1B	Chromatin Assembly Factor 1 Subunit B	Protein Coding	39	GC21P036385	0.745580971
COX8A	Cytochrome C Oxidase Subunit 8A	Protein Coding	39	GC11P064049	0.745580971
FGFBP1	Fibroblast Growth Factor Binding Protein 1	Protein Coding	39	GC04M015937	0.745580971
HPR	Haptoglobin-Related Protein	Protein Coding	39	GC16P072097	0.745580971
SLC35C1	Solute Carrier Family 35 Member C1	Protein Coding	39	GC11P046498	0.745580971
SUPT3H	SPT3 Homolog, SAGA And STAGA Complex Component	Protein Coding	39	GC06M066240	0.745580971
ASB2	Ankyrin Repeat And SOCS Box Containing 2	Protein Coding	38	GC14M093934	0.745580971
CSPG5	Chondroitin Sulfate Proteoglycan 5	Protein Coding	38	GC03M047562	0.745580971
FXR2	FMR1 Autosomal Homolog 2	Protein Coding	38	GC17M007781	0.745580971
LMCD1	LIM And Cysteine Rich Domains 1	Protein Coding	38	GC03P008603	0.745580971
LSM4	LSM4 Homolog, U6 Small Nuclear RNA And MRNA Degradation Associated	Protein Coding	38	GC19M018306	0.745580971
MTSS1	MTSS I-BAR Domain Containing 1	Protein Coding	38	GC08M124550	0.745580971
MYOM1	Myomesin 1	Protein Coding	38	GC18M003066	0.745580971
OBSCN	Obscurin, Cytoskeletal Calmodulin And Titin-Interacting RhoGEF	Protein Coding	38	GC01P228208	0.745580971
P2RX5	Purinergic Receptor P2X 5	Protein Coding	38	GC17M003672	0.745580971
PGK2	Phosphoglycerate Kinase 2	Protein Coding	38	GC06M049785	0.745580971
RFWD3	Ring Finger And WD Repeat Domain 3	Protein Coding	38	GC16M074621	0.745580971
SLCO1A2	Solute Carrier Organic Anion Transporter Family Member 1A2	Protein Coding	38	GC12M021264	0.745580971
AKR1E2	Aldo-Keto Reductase Family 1 Member E2	Protein Coding	37	GC10P004786	0.745580971
CALCB	Calcitonin Related Polypeptide Beta	Protein Coding	37	GC11P014904	0.745580971
CAPS	Calcyphosine	Protein Coding	37	GC19P005920	0.745580971
DKKL1	Dickkopf Like Acrosomal Protein 1	Protein Coding	37	GC19P049360	0.745580971
GMIP	GEM Interacting Protein	Protein Coding	37	GC19M019629	0.745580971
MEX3C	Mex-3 RNA Binding Family Member C	Protein Coding	37	GC18M051174	0.745580971
RIMS3	Regulating Synaptic Membrane Exocytosis 3	Protein Coding	37	GC01M040621	0.745580971
SNRPD1	Small Nuclear Ribonucleoprotein D1 Polypeptide	Protein Coding	37	GC18P021612	0.745580971
SNRPF	Small Nuclear Ribonucleoprotein Polypeptide F	Protein Coding	37	GC12P095858	0.745580971
CD163L1	CD163 Molecule Like 1	Protein Coding	36	GC12M007727	0.745580971
CLVS1	Clavesin 1	Protein Coding	36	GC08P060966	0.745580971
DNAAF2	Dynein Axonemal Assembly Factor 2	Protein Coding	36	GC14M049625	0.745580971
HCAR1	Hydroxycarboxylic Acid Receptor 1	Protein Coding	36	GC12M122726	0.745580971
IPPK	Inositol-Pentakisphosphate 2-Kinase	Protein Coding	36	GC09M092613	0.745580971
MRPS10	Mitochondrial Ribosomal Protein S10	Protein Coding	36	GC06M042206	0.745580971
SERPINB10	Serpin Family B Member 10	Protein Coding	36	GC18P063897	0.745580971
SNRPB2	Small Nuclear Ribonucleoprotein Polypeptide B2	Protein Coding	36	GC20P016730	0.745580971
HSF2BP	Heat Shock Transcription Factor 2 Binding Protein	Protein Coding	35	GC21M043453	0.745580971
YIPF1	Yip1 Domain Family Member 1	Protein Coding	35	GC01M053851	0.745580971
ZNF189	Zinc Finger Protein 189	Protein Coding	35	GC09P101398	0.745580971
PHYHIP	Phytanoyl-CoA 2-Hydroxylase Interacting Protein	Protein Coding	34	GC08M022219	0.745580971
ALKBH5	AlkB Homolog 5, RNA Demethylase	Protein Coding	33	GC17P018183	0.745580971
DPF3	Double PHD Fingers 3	Protein Coding	33	GC14M072610	0.745580971
HILPDA	Hypoxia Inducible Lipid Droplet Associated	Protein Coding	33	GC07P131916	0.745580971
KRT73	Keratin 73	Protein Coding	33	GC12M052607	0.745580971
SNTG1	Syntrophin Gamma 1	Protein Coding	33	GC08P049909	0.745580971
TMEM39A	Transmembrane Protein 39A	Protein Coding	33	GC03M119428	0.745580971
ZCCHC17	Zinc Finger CCHC-Type Containing 17	Protein Coding	33	GC01P031298	0.745580971
ZNF593	Zinc Finger Protein 593	Protein Coding	33	GC01P026171	0.745580971
ACTRT1	Actin Related Protein T1	Protein Coding	32	GC0XM128050	0.745580971
FAM13B	Family With Sequence Similarity 13 Member B	Protein Coding	32	GC05M137937	0.745580971
LARP1B	La Ribonucleoprotein 1B	Protein Coding	32	GC04P128061	0.745580971
LENG9	Leukocyte Receptor Cluster Member 9	Protein Coding	32	GC19M054461	0.745580971
KRCC1	Lysine Rich Coiled-Coil 1	Protein Coding	30	GC02M088027	0.745580971
RBM18	RNA Binding Motif Protein 18	Protein Coding	30	GC09M122237	0.745580971
TMEM39B	Transmembrane Protein 39B	Protein Coding	30	GC01P032073	0.745580971
C1orf210	Chromosome 1 Open Reading Frame 210	Protein Coding	29	GC01M043281	0.745580971
DZANK1	Double Zinc Ribbon And Ankyrin Repeat Domains 1	Protein Coding	29	GC20M018383	0.745580971
KIAA1549L	KIAA1549 Like	Protein Coding	29	GC11P033377	0.745580971
REP15	RAB15 Effector Protein	Protein Coding	29	GC12P027696	0.745580971
TMEM203	Transmembrane Protein 203	Protein Coding	29	GC09M137204	0.745580971
EVA1B	Eva-1 Homolog B	Protein Coding	28	GC01M036322	0.745580971
ZNF681	Zinc Finger Protein 681	Protein Coding	28	GC19M033716	0.745580971
TEX38	Testis Expressed 38	Protein Coding	26	GC01P046668	0.745580971
IHO1	Interactor Of HORMAD1 1	Protein Coding	25	GC03P049635	0.745580971
RBIS	Ribosomal Biogenesis Factor	Protein Coding	25	GC08M085215	0.745580971
BMP2KL	BMP2 Inducible Kinase Like (Pseudogene)	Pseudogene	9	GC0XP074185	0.745580971
TUBB	Tubulin Beta Class I	Protein Coding	48	GC06P083675	0.739390373
HSP90AB1	Heat Shock Protein 90 Alpha Family Class B Member 1	Protein Coding	46	GC06P044246	0.719819486
BAG3	BAG Cochaperone 3	Protein Coding	42	GC10P119651	0.719819486
OMA1	OMA1 Zinc Metallopeptidase	Protein Coding	36	GC01M058415	0.719819486
CLINT1	Clathrin Interactor 1	Protein Coding	38	GC05M157785	0.713886738
SRSF4	Serine And Arginine Rich Splicing Factor 4	Protein Coding	37	GC01M029147	0.713886738
NDUFA4L2	NDUFA4 Mitochondrial Complex Associated Like 2	Protein Coding	35	GC12M057234	0.713886738
RAC2	Rac Family Small GTPase 2	Protein Coding	49	GC22M037489	0.708111525
SMAD4	SMAD Family Member 4	Protein Coding	48	GC18P051028	0.708111525
BAP1	BRCA1 Associated Protein 1	Protein Coding	46	GC03M052401	0.708111525
NRG1	Neuregulin 1	Protein Coding	45	GC08P031639	0.708111525
ITGAX	Integrin Subunit Alpha X	Protein Coding	44	GC16P041137	0.708111525
SAE1	SUMO1 Activating Enzyme Subunit 1	Protein Coding	44	GC19P066970	0.708111525

ALAS2	5'-Aminolevulinate Synthase 2	Protein Coding	43	GC0XM055009	0.708111525
HNMT	Histamine N-Methyltransferase	Protein Coding	43	GC02P137964	0.708111525
KCNN3	Potassium Calcium-Activated Channel Subfamily N Member 3	Protein Coding	43	GC01M154697	0.708111525
MYBPC1	Myosin Binding Protein C1	Protein Coding	42	GC12P101568	0.708111525
UGCG	UDP-Glucose Ceramide Glucosyltransferase	Protein Coding	42	GC09P111896	0.708111525
HES1	Hes Family BHLH Transcription Factor 1	Protein Coding	41	GC03P194136	0.708111525
HHAT	Hedgehog Acyltransferase	Protein Coding	41	GC01P210328	0.708111525
VPS11	VPS11 Core Subunit Of CORVET And HOPS Complexes	Protein Coding	41	GC11P119067	0.708111525
GLS2	Glutaminase 2	Protein Coding	40	GC12M056470	0.708111525
PEMT	Phosphatidylethanolamine N-Methyltransferase	Protein Coding	39	GC17M017562	0.708111525
RNASE2	Ribonuclease A Family Member 2	Protein Coding	39	GC14P032471	0.708111525
HPS4	HPS4 Biogenesis Of Lysosomal Organelles Complex 3 Subunit 2	Protein Coding	38	GC22M026443	0.708111525
NMUR1	Neuromedin U Receptor 1	Protein Coding	38	GC02M231930	0.708111525
SNIP1	Smad Nuclear Interacting Protein 1	Protein Coding	38	GC01M037534	0.708111525
CDKL2	Cyclin Dependent Kinase Like 2	Protein Coding	37	GC04M075576	0.708111525
FAM20B	FAM20B Glycosaminoglycan Xylosylkinase	Protein Coding	37	GC01P179025	0.708111525
ABLIM3	Actin Binding LIM Protein Family Member 3	Protein Coding	36	GC05P149141	0.708111525
GPR26	G Protein-Coupled Receptor 26	Protein Coding	36	GC10P123666	0.708111525
NKAP	NFKB Activating Protein	Protein Coding	36	GC0XM120138	0.708111525
PLAGL2	PLAG1 Like Zinc Finger 2	Protein Coding	36	GC20M032192	0.708111525
RHOU	Ras Homolog Family Member U	Protein Coding	36	GC01P228644	0.708111525
IL9R	Interleukin 9 Receptor	Protein Coding	35	GC0XP155997	0.708111525
WDR46	WD Repeat Domain 46	Protein Coding	35	GC06M033279	0.708111525
GPHA2	Glycoprotein Hormone Subunit Alpha 2	Protein Coding	34	GC11M089739	0.708111525
MICALL2	MICAL Like 2	Protein Coding	34	GC07M001650	0.708111525
NBPF3	NBPF Member 3	Protein Coding	34	GC01P021857	0.708111525
UBL5	Ubiquitin Like 5	Protein Coding	34	GC19P009827	0.708111525
ELMOD1	ELMO Domain Containing 1	Protein Coding	33	GC11P107592	0.708111525
IFNL3	Interferon Lambda 3	Protein Coding	33	GC19M039243	0.708111525
MT-ND5	Mitochondrially Encoded NADH:Ubiquinone Oxidoreductase Core Subunit 5	Protein Coding	33	GCMTP012339	0.708111525
DPH6	Diphthamine Biosynthesis 6	Protein Coding	32	GC15M035332	0.708111525
DTWD2	DTW Domain Containing 2	Protein Coding	32	GC05M118836	0.708111525
LEPROT	Leptin Receptor Overlapping Transcript	Protein Coding	32	GC01P065420	0.708111525
LRRC46	Leucine Rich Repeat Containing 46	Protein Coding	32	GC17P047831	0.708111525
MIS18A	MIS18 Kinetochore Protein A	Protein Coding	32	GC21M032154	0.708111525
OSCP1	Organic Solute Carrier Partner 1	Protein Coding	32	GC01M036415	0.708111525
ZBED3	Zinc Finger BED-Type Containing 3	Protein Coding	30	GC05M077072	0.708111525
ACSM6	Acyl-CoA Synthetase Medium Chain Family Member 6	Protein Coding	29	GC10P095194	0.708111525
SELENOI	Selenoprotein I	Protein Coding	28	GC02P026310	0.708111525
TDRD12	Tudor Domain Containing 12	Protein Coding	28	GC19P032719	0.708111525
UBL4B	Ubiquitin Like 4B	Protein Coding	28	GC01P110383	0.708111525
MRGPRE	MAS Related GPR Family Member E	Protein Coding	27	GC11M003562	0.708111525
MUCL3	Mucin Like 3	Protein Coding	27	GC06P085587	0.708111525
OR13C4	Olfactory Receptor Family 13 Subfamily C Member 4	Protein Coding	27	GC09M104526	0.708111525
TSGA13	Testis Specific 13	Protein Coding	26	GC07M130668	0.708111525
PRELID3A	PRELI Domain Containing 3A	Protein Coding	24	GC18P017447	0.708111525
MIR346	MicroRNA 346	RNA Gene	21	GC10M086264	0.708111525
MIR224	MicroRNA 224	RNA Gene	18	GC0XM151958	0.708111525
LINC01104	Long Intergenic Non-Protein Coding RNA 1104	RNA Gene	14	GC02P100208	0.708111525
EPHA3	EPH Receptor A3	Protein Coding	44	GC03P089077	0.698537469
DNAJB6	DnaJ Heat Shock Protein Family (Hsp40) Member B6	Protein Coding	41	GC07P157335	0.698537469
TUBA1B	Tubulin Alpha 1b	Protein Coding	41	GC12M049127	0.698537469
CANX	Calnexin	Protein Coding	42	GC05P179678	0.684836209
CNOT7	CCR4-NOT Transcription Complex Subunit 7	Protein Coding	38	GC08M017224	0.67258209
ACTG1	Actin Gamma 1	Protein Coding	47	GC17M081509	0.6482777
STUB1	STIP1 Homology And U-Box Containing Protein 1	Protein Coding	43	GC16P011630	0.6482777
CCT2	Chaperonin Containing TCP1 Subunit 2	Protein Coding	40	GC12P069585	0.6482777
TGM2	Transglutaminase 2	Protein Coding	46	GC20M038127	0.616583467
FOXM1	Forkhead Box M1	Protein Coding	42	GC12M002857	0.610922813
PFKFB3	6-Phosphofructo-2-Kinase/Fructose-2,6-Biphosphatase 3	Protein Coding	43	GC10P006144	0.575278819
ULK2	Unc-51 Like Autophagy Activating Kinase 2	Protein Coding	39	GC17M025780	0.575278819
KDR	Kinase Insert Domain Receptor	Protein Coding	51	GC04M055078	0.572863758
NR3C1	Nuclear Receptor Subfamily 3 Group C Member 1	Protein Coding	48	GC05M143277	0.572863758
NF2	NF2, Moesin-Ezrin-Radixin Like (MERLIN) Tumor Suppressor	Protein Coding	46	GC22P029603	0.572863758
NAGA	Alpha-N-Acetylgalactosaminidase	Protein Coding	44	GC22M042058	0.572863758
DHX38	DEAH-Box Helicase 38	Protein Coding	42	GC16P072127	0.572863758
EIF2AK1	Eukaryotic Translation Initiation Factor 2 Alpha Kinase 1	Protein Coding	42	GC07M006022	0.572863758
GNE	Glucosamine (UDP-N-Acetyl)-2-Epimerase/N-Acetylmannosamine Kinase	Protein Coding	42	GC09M036214	0.572863758
KCNH3	Potassium Voltage-Gated Channel Subfamily H Member 3	Protein Coding	42	GC12P049539	0.572863758
LRPAP1	LDL Receptor Related Protein Associated Protein 1	Protein Coding	42	GC04M003508	0.572863758
SATB1	SATB Homeobox 1	Protein Coding	42	GC03M020812	0.572863758
COX6B1	Cytochrome C Oxidase Subunit 6B1	Protein Coding	41	GC19P066543	0.572863758
GPC1	Glypican 1	Protein Coding	41	GC02P240435	0.572863758
KCNAB2	Potassium Voltage-Gated Channel Subfamily A Regulatory Beta Subunit 2	Protein Coding	41	GC01P006020	0.572863758
TRPC5	Transient Receptor Potential Cation Channel Subfamily C Member 5	Protein Coding	41	GC0XM111774	0.572863758
IL13RA1	Interleukin 13 Receptor Subunit Alpha 1	Protein Coding	40	GC0XP118727	0.572863758
MYLK3	Myosin Light Chain Kinase 3	Protein Coding	40	GC16M047008	0.572863758
NADSYN1	NAD Synthetase 1	Protein Coding	40	GC11P071454	0.572863758
P2RY4	Pyrimidinergic Receptor P2Y4	Protein Coding	39	GC0XM070258	0.572863758
DAAM2	Dishevelled Associated Activator Of Morphogenesis 2	Protein Coding	38	GC06P039792	0.572863758
DUSP22	Dual Specificity Phosphatase 22	Protein Coding	38	GC06P000302	0.572863758
GAS2	Growth Arrest Specific 2	Protein Coding	38	GC11P022626	0.572863758
MLLT3	MLLT3 Super Elongation Complex Subunit	Protein Coding	38	GC09M020341	0.572863758
NTN4	Netrin 4	Protein Coding	38	GC12M095657	0.572863758
RGS17	Regulator Of G Protein Signaling 17	Protein Coding	38	GC06M153004	0.572863758
SLC25A36	Solute Carrier Family 25 Member 36	Protein Coding	38	GC03P140941	0.572863758
CETN1	Centrin 1	Protein Coding	37	GC18P000580	0.572863758
CDK2AP1	Cyclin Dependent Kinase 2 Associated Protein 1	Protein Coding	36	GC12M123250	0.572863758
IMP3	IMP U3 Small Nucleolar Ribonucleoprotein 3	Protein Coding	36	GC15M075639	0.572863758
NLRP14	NLR Family Pyrin Domain Containing 14	Protein Coding	36	GC11P007020	0.572863758
SCRN1	Secernin 1	Protein Coding	36	GC07M030610	0.572863758
MS4A4A	Membrane Spanning 4-Domains A4A	Protein Coding	33	GC11P060185	0.572863758
HEATR6	HEAT Repeat Containing 6	Protein Coding	32	GC17M060041	0.572863758
KIAA0232	KIAA0232	Protein Coding	32	GC04P006863	0.572863758
FOXL2NB	FOXL2 Neighbor	Protein Coding	24	GC03P138947	0.572863758
LINC01205	Long Intergenic Non-Protein Coding RNA 1205	RNA Gene	13	GC03P109409	0.572863758
TPI1P1	Triosephosphate Isomerase 1 Pseudogene 1	Pseudogene	9	GC01P076699	0.572863758
PRDX6	Peroxiredoxin 6	Protein Coding	44	GC01P173477	0.531706035

LONP1	Lon Peptidase 1, Mitochondrial	Protein Coding	45	GC19M005691	0.528313458
RPL29P31	Ribosomal Protein L29 Pseudogene 31	Pseudogene	6	GC17P043476	0.528313458
AIFM1	Apoptosis Inducing Factor Mitochondria Associated 1	Protein Coding	46	GC0XM130129	0.496380925
TNF	Tumor Necrosis Factor	Protein Coding	49	GC06P083695	0.469899833
EPG5	Ectopic P-Granules 5 Autophagy Tethering Factor	Protein Coding	37	GC18M045800	0.469899833
HMOX1	Heme Oxygenase 1	Protein Coding	49	GC22P035380	0.455527961
APOE	Apolipoprotein E	Protein Coding	48	GC19P066879	0.455527961
TRPM2	Transient Receptor Potential Cation Channel Subfamily M Member 2	Protein Coding	40	GC21P044350	0.455527961
MGRN1	Mahogunin Ring Finger 1	Protein Coding	38	GC16P011777	0.455527961
ITGB4	Integrin Subunit Beta 4	Protein Coding	47	GC17P075721	0.431987673
XIAP	X-Linked Inhibitor Of Apoptosis	Protein Coding	46	GC0XP123859	0.431987673
DIABLO	Diablo IAP-Binding Mitochondrial Protein	Protein Coding	45	GC12M122300	0.431987673
MYO6	Myosin VI	Protein Coding	44	GC06P075749	0.431987673
HSPA2	Heat Shock Protein Family A (Hsp70) Member 2	Protein Coding	43	GC14P064535	0.431987673
PDK2	Pyruvate Dehydrogenase Kinase 2	Protein Coding	42	GC17P056029	0.431987673
SSBP1	Single Stranded DNA Binding Protein 1	Protein Coding	39	GC07P148685	0.431987673
MIR218-2	MicroRNA 218-2	RNA Gene	20	GC05M168768	0.431987673
MIR218-1	MicroRNA 218-1	RNA Gene	18	GC04P020849	0.431987673
USP30-AS1	USP30 Antisense RNA 1	RNA Gene	13	GC12M109052	0.431987673
MIEF2	Mitochondrial Elongation Factor 2	Protein Coding	33	GC17P018262	0.428595155
FOS	Fos Proto-Oncogene, AP-1 Transcription Factor Subunit	Protein Coding	49	GC14P075278	0.405268252
PARP1	Poly(ADP-Ribose) Polymerase 1	Protein Coding	47	GC01M226360	0.405268252
RPS6KB1	Ribosomal Protein S6 Kinase B1	Protein Coding	46	GC17P059893	0.405268252
CLU	Clusterin	Protein Coding	45	GC08M027596	0.405268252
MYCN	MYCN Proto-Oncogene, BHLH Transcription Factor	Protein Coding	45	GC02P015949	0.405268252
IGFBP3	Insulin Like Growth Factor Binding Protein 3	Protein Coding	44	GC07M045912	0.405268252
PMPCB	Peptidase, Mitochondrial Processing Subunit Beta	Protein Coding	43	GC07P103297	0.405268252
RACGAP1	Rac GTPase Activating Protein 1	Protein Coding	43	GC12M049978	0.405268252
STK38	Serine/Threonine Kinase 38	Protein Coding	42	GC06M036493	0.405268252
TMSB4X	Thymosin Beta 4 X-Linked	Protein Coding	39	GC0XP012975	0.405268252
MRPS7	Mitochondrial Ribosomal Protein S7	Protein Coding	38	GC17P075579	0.405268252
IDH2	Isocitrate Dehydrogenase (NADP(+)) 2	Protein Coding	50	GC15M090083	0.373574018
CASP8	Caspase 8	Protein Coding	49	GC02P201233	0.373574018
IKBKG	Inhibitor Of Nuclear Factor Kappa B Kinase Regulatory Subunit Gamma	Protein Coding	46	GC0XP154541	0.373574018
NBN	Nibrin	Protein Coding	45	GC08M089933	0.373574018
IRF3	Interferon Regulatory Factor 3	Protein Coding	44	GC19M049659	0.373574018
KMO	Kynurenine 3-Monooxygenase	Protein Coding	44	GC01P241532	0.373574018
CHKB	Choline Kinase Beta	Protein Coding	43	GC22M050578	0.373574018
HSPA6	Heat Shock Protein Family A (Hsp70) Member 6	Protein Coding	43	GC01P161524	0.373574018
PMPCA	Peptidase, Mitochondrial Processing Subunit Alpha	Protein Coding	43	GC09P136410	0.373574018
SNCAIP	Synuclein Alpha Interacting Protein	Protein Coding	42	GC05P122311	0.373574018
TRAF2	TNF Receptor Associated Factor 2	Protein Coding	42	GC09P136881	0.373574018
RBCK1	RANBP2-Type And C3HC4-Type Zinc Finger Containing 1	Protein Coding	41	GC20P000407	0.373574018
UTRN	Utrophin	Protein Coding	40	GC06P144285	0.373574018
PACRG	Parkin Coregulated	Protein Coding	38	GC06P162727	0.373574018
PACS2	Phosphofurin Acidic Cluster Sorting Protein 2	Protein Coding	38	GC14P105300	0.373574018
SLC39A1	Solute Carrier Family 39 Member 1	Protein Coding	38	GC01M153960	0.373574018
TMEM59	Transmembrane Protein 59	Protein Coding	38	GC01M054031	0.373574018
MIR204	MicroRNA 204	RNA Gene	24	GC09M070809	0.373574018
MIR4673	MicroRNA 4673	RNA Gene	12	GC09M137528	0.373574018
MMP2	Matrix Metallopeptidase 2	Protein Coding	50	GC16P055390	0.332269341
CCND1	Cyclin D1	Protein Coding	49	GC11P069641	0.332269341
MAOA	Monoamine Oxidase A	Protein Coding	48	GC0XP043654	0.332269341
ALDH7A1	Aldehyde Dehydrogenase 7 Family Member A1	Protein Coding	46	GC05M126541	0.332269341
IFIH1	Interferon Induced With Helicase C Domain 1	Protein Coding	45	GC02M162267	0.332269341
P2RX7	Purinergic Receptor P2X 7	Protein Coding	45	GC12P126220	0.332269341
SCO1	Synthesis Of Cytochrome C Oxidase 1	Protein Coding	45	GC17M010672	0.332269341
SUCLA2	Succinate-CoA Ligase ADP-Forming Subunit Beta	Protein Coding	45	GC13M047745	0.332269341
UQCRFS1	Ubiquinol-Cytochrome C Reductase, Rieske Iron-Sulfur Polypeptide 1	Protein Coding	45	GC19M029205	0.332269341
CYC1	Cytochrome C1	Protein Coding	44	GC08P144095	0.332269341
APOL1	Apolipoprotein L1	Protein Coding	43	GC22P036253	0.332269341
APTX	Aprataxin	Protein Coding	43	GC09M032886	0.332269341
CLPB	Caseinolytic Mitochondrial Matrix Peptidase Chaperone Subunit B	Protein Coding	43	GC11M090311	0.332269341
NR4A3	Nuclear Receptor Subfamily 4 Group A Member 3	Protein Coding	43	GC09P099821	0.332269341
ACAA2	Acetyl-CoA Acyltransferase 2	Protein Coding	42	GC18M049782	0.332269341
POLRMT	RNA Polymerase Mitochondrial	Protein Coding	42	GC19M000617	0.332269341
USP4	Ubiquitin Specific Peptidase 4	Protein Coding	42	GC03M049277	0.332269341
APLNR	Apelin Receptor	Protein Coding	41	GC11M057233	0.332269341
LGALS1	Galectin 1	Protein Coding	41	GC22P037675	0.332269341
MRPL3	Mitochondrial Ribosomal Protein L3	Protein Coding	41	GC03M131462	0.332269341
EDC3	Enhancer Of MRNA Decapping 3	Protein Coding	40	GC15M090236	0.332269341
IDH3G	Isocitrate Dehydrogenase (NAD(+)) 3 Non-Catalytic Subunit Gamma	Protein Coding	40	GC0XM153785	0.332269341
SUCLG2	Succinate-CoA Ligase GDP-Forming Subunit Beta	Protein Coding	40	GC03M067358	0.332269341
AK4	Adenylate Kinase 4	Protein Coding	39	GC01P065147	0.332269341
DNAJC3	DnaJ Heat Shock Protein Family (Hsp40) Member C3	Protein Coding	39	GC13P095677	0.332269341
GLRX5	Glutaredoxin 5	Protein Coding	39	GC14P095533	0.332269341
SHARPIN	SHANK Associated RH Domain Interactor	Protein Coding	39	GC08M144098	0.332269341
DNAJA3	DnaJ Heat Shock Protein Family (Hsp40) Member A3	Protein Coding	38	GC16P004425	0.332269341
ERAL1	Era Like 12S Mitochondrial RRNA Chaperone 1	Protein Coding	38	GC17P054927	0.332269341
NLRX1	NLR Family Member X1	Protein Coding	38	GC11P119166	0.332269341
ABHD11	Abhydrolase Domain Containing 11	Protein Coding	37	GC07M073736	0.332269341
CHD6	Chromodomain Helicase DNA Binding Protein 6	Protein Coding	37	GC20M041402	0.332269341
COG1	Component Of Oligomeric Golgi Complex 1	Protein Coding	37	GC17P073193	0.332269341
EDC4	Enhancer Of MRNA Decapping 4	Protein Coding	37	GC16P067873	0.332269341
IMMP2L	Inner Mitochondrial Membrane Peptidase Subunit 2	Protein Coding	37	GC07M110663	0.332269341
MRPL13	Mitochondrial Ribosomal Protein L13	Protein Coding	37	GC08M120377	0.332269341
RASD2	RASD Family Member 2	Protein Coding	37	GC22P037035	0.332269341
APLN	Apelin	Protein Coding	36	GC0XM129645	0.332269341
CNTNAP4	Contactin Associated Protein Family Member 4	Protein Coding	36	GC16P076278	0.332269341
IMMP1L	Inner Mitochondrial Membrane Peptidase Subunit 1	Protein Coding	36	GC11M031432	0.332269341
MRPL28	Mitochondrial Ribosomal Protein L28	Protein Coding	36	GC16M000366	0.332269341
JMY	Junction Mediating And Regulatory Protein, P53 Cofactor	Protein Coding	35	GC05P079236	0.332269341
MRPL37	Mitochondrial Ribosomal Protein L37	Protein Coding	35	GC01P054185	0.332269341
OCIAD2	OCIA Domain Containing 2	Protein Coding	35	GC04M048887	0.332269341
ZNF185	Zinc Finger Protein 185 With LIM Domain	Protein Coding	35	GC0XP152898	0.332269341
ATG101	Autophagy Related 101	Protein Coding	34	GC12P052069	0.332269341
MRPL35	Mitochondrial Ribosomal Protein L35	Protein Coding	34	GC02P086199	0.332269341
MRPL41	Mitochondrial Ribosomal Protein L41	Protein Coding	34	GC09P137551	0.332269341

MRPL49	Mitochondrial Ribosomal Protein L49	Protein Coding	34	GC11P069948	0.332269341
SPC24	SPC24 Component Of NDC80 Kinetochore Complex	Protein Coding	33	GC19M011131	0.332269341
WHAMM	WASP Homolog Associated With Actin, Golgi Membranes And Microtubules	Protein Coding	33	GC15P117911	0.332269341
STING1	Stimulator Of Interferon Response CGAMP Interactor 1	Protein Coding	32	GC05M139476	0.332269341
ZNF787	Zinc Finger Protein 787	Protein Coding	30	GC19M066427	0.332269341
CISD3	CDGSH Iron Sulfur Domain 3	Protein Coding	29	GC17P038730	0.332269341
HGH1	HGH1 Homolog	Protein Coding	28	GC08P144137	0.332269341
HDHD5	Haloacid Dehalogenase Like Hydrolase Domain Containing 5	Protein Coding	26	GC22M017195	0.332269341
MIGA2	Mitoguardin 2	Protein Coding	25	GC09P129037	0.332269341
MIR33A	MicroRNA 33a	RNA Gene	20	GC22P041900	0.332269341
NUPR1	Nuclear Protein 1, Transcriptional Regulator	Protein Coding	34	GC16M028532	0.328876823
TMEM150B	Transmembrane Protein 150B	Protein Coding	28	GC19M066391	0.328876823
CD44	CD44 Molecule (Indian Blood Group)	Protein Coding	45	GC11P035139	0.299154997
HDAC3	Histone Deacetylase 3	Protein Coding	47	GC05M141583	0.28204602
RHOT2	Ras Homolog Family Member T2	Protein Coding	37	GC16P011626	0.28204602
ERCC8	ERCC Excision Repair 8, CSA Ubiquitin Ligase Complex Subunit	Protein Coding	41	GC05M060894	0.263829887
LMNA	Lamin A/C	Protein Coding	48	GC01P156082	0.244259015
SLC25A3	Solute Carrier Family 25 Member 3	Protein Coding	43	GC12P098593	0.244259015
HNRNPM	Heterogeneous Nuclear Ribonucleoprotein M	Protein Coding	38	GC19P008444	0.244259015
CAPN1	Calpain 1	Protein Coding	48	GC11P069958	0.232551023
HSPG2	Heparan Sulfate Proteoglycan 2	Protein Coding	46	GC01M021822	0.232551023
TNFRSF1A	TNF Receptor Superfamily Member 1A	Protein Coding	46	GC12M006328	0.232551023
CPT1C	Carnitine Palmitoyltransferase 1C	Protein Coding	41	GC19P049690	0.232551023
USP19	Ubiquitin Specific Peptidase 19	Protein Coding	38	GC03M051164	0.232551023
PPAN	Peter Pan Homolog	Protein Coding	33	GC19P010452	0.232551023
PVT1	Pvt1 Oncogene	RNA Gene	26	GC08P127869	0.232551023
MIR195	MicroRNA 195	RNA Gene	20	GC17M007018	0.232551023
NPM1	Nucleophosmin 1	Protein Coding	48	GC05P171387	0.222976953
HSPA5	Heat Shock Protein Family A (Hsp70) Member 5	Protein Coding	46	GC09M125234	0.222976953
DDX5	DEAD-Box Helicase 5	Protein Coding	44	GC17M064498	0.222976953
HNRNPA2B1	Heterogeneous Nuclear Ribonucleoprotein A2/B1	Protein Coding	44	GC07M026174	0.222976953
PKM	Pyruvate Kinase M1/2	Protein Coding	44	GC15M072199	0.222976953
HNRNPK	Heterogeneous Nuclear Ribonucleoprotein K	Protein Coding	43	GC09M093950	0.222976953
ATP13A2	ATPase Cation Transporting 13A2	Protein Coding	42	GC01M016985	0.222976953
TUBB4B	Tubulin Beta 4B Class IVb	Protein Coding	42	GC09P137241	0.222976953
HNRNPU	Heterogeneous Nuclear Ribonucleoprotein U	Protein Coding	41	GC01M244844	0.222976953
MATR3	Matrin 3	Protein Coding	41	GC05P139274	0.222976953
TUBB6	Tubulin Beta 6 Class V	Protein Coding	40	GC18P012307	0.222976953
DDX17	DEAD-Box Helicase 17	Protein Coding	39	GC22M038483	0.222976953
RPS16	Ribosomal Protein S16	Protein Coding	39	GC19M039433	0.222976953
HNRNPA3	Heterogeneous Nuclear Ribonucleoprotein A3	Protein Coding	38	GC02P177212	0.222976953
HNRNPH1	Heterogeneous Nuclear Ribonucleoprotein H1	Protein Coding	38	GC05M179614	0.222976953
ADRM1	ADRM1 26S Proteasome Ubiquitin Receptor	Protein Coding	37	GC20P062302	0.222976953
ATP5F1A	ATP Synthase F1 Subunit Alpha	Protein Coding	36	GC18M046081	0.222976953
ABCB1	ATP Binding Cassette Subfamily B Member 1	Protein Coding	49	GC07M087504	0.19943665
CALR	Calreticulin	Protein Coding	49	GC19P012938	0.19943665
FOXO1	Forkhead Box O1	Protein Coding	47	GC13M040555	0.19943665
IMPDH2	Inosine Monophosphate Dehydrogenase 2	Protein Coding	46	GC03M051162	0.19943665
PRDX1	Peroxiredoxin 1	Protein Coding	46	GC01M045511	0.19943665
CASP14	Caspase 14	Protein Coding	45	GC19P015049	0.19943665
HSP90B1	Heat Shock Protein 90 Beta Family Member 1	Protein Coding	45	GC12P103930	0.19943665
LMNB1	Lamin B1	Protein Coding	45	GC05P126776	0.19943665
DDX3X	DEAD-Box Helicase 3 X-Linked	Protein Coding	44	GC0XP041333	0.19943665
HNRNPA1	Heterogeneous Nuclear Ribonucleoprotein A1	Protein Coding	44	GC12P054280	0.19943665
PSMA7	Proteasome 20S Subunit Alpha 7	Protein Coding	44	GC20M062136	0.19943665
ABHD5	Abhydrolase Domain Containing 5, Lysophosphatidic Acid Acyltransferase	Protein Coding	43	GC03P043707	0.19943665
C1QBP	Complement C1q Binding Protein	Protein Coding	43	GC17M005432	0.19943665
PRKCSH	Protein Kinase C Substrate 80K-H	Protein Coding	43	GC19P011435	0.19943665
PSMB5	Proteasome 20S Subunit Beta 5	Protein Coding	43	GC14M023016	0.19943665
XRCC6	X-Ray Repair Cross Complementing 6	Protein Coding	43	GC22P041622	0.19943665
RPN1	Ribophorin I	Protein Coding	42	GC03M128619	0.19943665
RPS14	Ribosomal Protein S14	Protein Coding	42	GC05M150443	0.19943665
SFPQ	Splicing Factor Proline And Glutamine Rich	Protein Coding	42	GC01M035176	0.19943665
SNRNP200	Small Nuclear Ribonucleoprotein U5 Subunit 200	Protein Coding	42	GC02M098261	0.19943665
TGM3	Transglutaminase 3	Protein Coding	42	GC20P002296	0.19943665
PABPC1	Poly(A) Binding Protein Cytoplasmic 1	Protein Coding	41	GC08M100685	0.19943665
PSMD3	Proteasome 26S Subunit, Non-ATPase 3	Protein Coding	41	GC17P039980	0.19943665
RPL13	Ribosomal Protein L13	Protein Coding	41	GC16P091315	0.19943665
RPL22	Ribosomal Protein L22	Protein Coding	41	GC01M006189	0.19943665
RPL7	Ribosomal Protein L7	Protein Coding	41	GC08M073290	0.19943665
RPS6	Ribosomal Protein S6	Protein Coding	41	GC09M019375	0.19943665
UBQLN2	Ubiquilin 2	Protein Coding	41	GC0XP056563	0.19943665
CCT3	Chaperonin Containing TCP1 Subunit 3	Protein Coding	40	GC01M156308	0.19943665
DHX9	DExH-Box Helicase 9	Protein Coding	40	GC01P182839	0.19943665
EIF4B	Eukaryotic Translation Initiation Factor 4B	Protein Coding	40	GC12P053006	0.19943665
HNRNPC	Heterogeneous Nuclear Ribonucleoprotein C	Protein Coding	40	GC14M022503	0.19943665
PDIA4	Protein Disulfide Isomerase Family A Member 4	Protein Coding	40	GC07M149003	0.19943665
PSMC1	Proteasome 26S Subunit, ATPase 1	Protein Coding	40	GC14P090256	0.19943665
RPL12	Ribosomal Protein L12	Protein Coding	40	GC09M127447	0.19943665
RPL23A	Ribosomal Protein L23a	Protein Coding	40	GC17P028719	0.19943665
SART1	Spliceosome Associated Factor 1, Recruiter Of U4/U6.U5 Tri-SnRNP	Protein Coding	40	GC11P070020	0.19943665
IGF2BP1	Insulin Like Growth Factor 2 MRNA Binding Protein 1	Protein Coding	39	GC17P055998	0.19943665
IRS4	Insulin Receptor Substrate 4	Protein Coding	39	GC0XM108720	0.19943665
SYNCRIP	Synaptotagmin Binding Cytoplasmic RNA Interacting Protein	Protein Coding	39	GC06M085607	0.19943665
HNRNPF	Heterogeneous Nuclear Ribonucleoprotein F	Protein Coding	38	GC10M043385	0.19943665
HNRNPR	Heterogeneous Nuclear Ribonucleoprotein R	Protein Coding	38	GC01M023303	0.19943665
MARCKS	Myristoylated Alanine Rich Protein Kinase C Substrate	Protein Coding	38	GC06P113857	0.19943665
PSMD1	Proteasome 26S Subunit, Non-ATPase 1	Protein Coding	38	GC02P231056	0.19943665
RPS8	Ribosomal Protein S8	Protein Coding	38	GC01P044775	0.19943665
NACA	Nascent Polypeptide Associated Complex Subunit Alpha	Protein Coding	37	GC12M056712	0.19943665
PTCD3	Pentatricopeptide Repeat Domain 3	Protein Coding	37	GC02P086106	0.19943665
CHCHD6	Coiled-Coil-Helix-Coiled-Coil-Helix Domain Containing 6	Protein Coding	34	GC03P126704	0.19943665
MIR27A	MicroRNA 27a	RNA Gene	23	GC19M014484	0.19943665
MIR27B	MicroRNA 27b	RNA Gene	21	GC09P095097	0.19943665
EEF1A1P5	Eukaryotic Translation Elongation Factor 1 Alpha 1 Pseudogene 5	Pseudogene	14	GC09P133019	0.19943665
ABL1	ABL Proto-Oncogene 1, Non-Receptor Tyrosine Kinase	Protein Coding	50	GC09P130713	0.172717214
ATP1A1	ATPase Na+/K+ Transporting Subunit Alpha 1	Protein Coding	48	GC01P116372	0.172717214
CAD	Carbamoyl-Phosphate Synthetase 2, Aspartate Transcarbamylase, And Dihydroorotase	Protein Coding	48	GC02P027217	0.172717214

FASN	Fatty Acid Synthase	Protein Coding	47	GC17M082078	0.172717214
ANXA2	Annexin A2	Protein Coding	46	GC15M060347	0.172717214
MDH2	Malate Dehydrogenase 2	Protein Coding	46	GC07P076048	0.172717214
RPL11	Ribosomal Protein L11	Protein Coding	46	GC01P023691	0.172717214
SLC6A1	Solute Carrier Family 6 Member 1	Protein Coding	46	GC03P012472	0.172717214
ACTN4	Actinin Alpha 4	Protein Coding	45	GC19P038647	0.172717214
CLTC	Clathrin Heavy Chain	Protein Coding	45	GC17P059619	0.172717214
ERCC6	ERCC Excision Repair 6, Chromatin Remodeling Factor	Protein Coding	45	GC10M049454	0.172717214
IL1B	Interleukin 1 Beta	Protein Coding	45	GC02M112829	0.172717214
MCM2	Minichromosome Maintenance Complex Component 2	Protein Coding	45	GC03P127598	0.172717214
MTHFD1	Methylenetetrahydrofolate Dehydrogenase, Cyclohydrolase And Formyltetrahydrofolate Synthetase 1	Protein Coding	45	GC14P064388	0.172717214
PPP2R1A	Protein Phosphatase 2 Scaffold Subunit Aalpha	Protein Coding	45	GC19P067249	0.172717214
SLC25A1	Solute Carrier Family 25 Member 1	Protein Coding	45	GC22M019355	0.172717214
SLC25A13	Solute Carrier Family 25 Member 13	Protein Coding	45	GC07M096120	0.172717214
CFL1	Cofilin 1	Protein Coding	44	GC11M065823	0.172717214
CLPP	Caseinolytic Mitochondrial Matrix Peptidase Proteolytic Subunit	Protein Coding	44	GC19P006629	0.172717214
HSD17B10	Hydroxysteroid 17-Beta Dehydrogenase 10	Protein Coding	44	GC0XM053431	0.172717214
MCM5	Minichromosome Maintenance Complex Component 5	Protein Coding	44	GC22P035400	0.172717214
RPSA	Ribosomal Protein SA	Protein Coding	44	GC03P039406	0.172717214
SET	SET Nuclear Proto-Oncogene	Protein Coding	44	GC09P128960	0.172717214
TLR9	Toll Like Receptor 9	Protein Coding	44	GC03M052222	0.172717214
DYNC1H1	Dynein Cytoplasmic 1 Heavy Chain 1	Protein Coding	43	GC14P109704	0.172717214
MCM7	Minichromosome Maintenance Complex Component 7	Protein Coding	43	GC07M100092	0.172717214
RPL10	Ribosomal Protein L10	Protein Coding	43	GC0XP154389	0.172717214
RPL21	Ribosomal Protein L21	Protein Coding	43	GC13P027251	0.172717214
RUVBL2	RuvB Like AAA ATPase 2	Protein Coding	43	GC19P048993	0.172717214
PSMD2	Proteasome 26S Subunit Ubiquitin Receptor, Non-ATPase 2	Protein Coding	42	GC03P184298	0.172717214
PSMD4	Proteasome 26S Subunit Ubiquitin Receptor, Non-ATPase 4	Protein Coding	42	GC01P151256	0.172717214
RPS20	Ribosomal Protein S20	Protein Coding	42	GC08M056067	0.172717214
RPS3	Ribosomal Protein S3	Protein Coding	42	GC11P077882	0.172717214
SNRPB	Small Nuclear Ribonucleoprotein Polypeptides B And B1	Protein Coding	42	GC20M002461	0.172717214
HDLBP	High Density Lipoprotein Binding Protein	Protein Coding	41	GC02M241227	0.172717214
NCL	Nucleolin	Protein Coding	41	GC02M231453	0.172717214
RBBP4	RB Binding Protein 4, Chromatin Remodeling Factor	Protein Coding	41	GC01P032651	0.172717214
RPL7A	Ribosomal Protein L7a	Protein Coding	41	GC09P133348	0.172717214
RPS15A	Ribosomal Protein S15a	Protein Coding	41	GC16M018781	0.172717214
RPS2	Ribosomal Protein S2	Protein Coding	41	GC16M007099	0.172717214
SNAP29	Synaptosome Associated Protein 29	Protein Coding	41	GC22P020859	0.172717214
SON	SON DNA And RNA Binding Protein	Protein Coding	41	GC21P033542	0.172717214
CCT4	Chaperonin Containing TCP1 Subunit 4	Protein Coding	40	GC02M061868	0.172717214
DNAJA1	DnaJ Heat Shock Protein Family (Hsp40) Member A1	Protein Coding	40	GC09P033025	0.172717214
MAGED1	MAGE Family Member D1	Protein Coding	40	GC0XP051803	0.172717214
RPL8	Ribosomal Protein L8	Protein Coding	40	GC08M145452	0.172717214
RPLP0	Ribosomal Protein Lateral Stalk Subunit P0	Protein Coding	40	GC12M120196	0.172717214
RPS3A	Ribosomal Protein S3A	Protein Coding	40	GC04P151099	0.172717214
CCT6A	Chaperonin Containing TCP1 Subunit 6A	Protein Coding	39	GC07P056051	0.172717214
PPY	Pancreatic Polypeptide	Protein Coding	39	GC17M043940	0.172717214
PSMC2	Proteasome 26S Subunit, ATPase 2	Protein Coding	39	GC07P103328	0.172717214
RCN2	Reticulocalbin 2	Protein Coding	39	GC15P076931	0.172717214
AMOT	Angiomotin	Protein Coding	38	GC0XM112774	0.172717214
BAG2	BAG Cochaperone 2	Protein Coding	38	GC06P057172	0.172717214
LRRFIP1	LRR Binding FLII Interacting Protein 1	Protein Coding	38	GC02P237627	0.172717214
PDIA6	Protein Disulfide Isomerase Family A Member 6	Protein Coding	38	GC02M010784	0.172717214
RPL14	Ribosomal Protein L14	Protein Coding	38	GC03P040458	0.172717214
RPL6	Ribosomal Protein L6	Protein Coding	38	GC12M112320	0.172717214
RPS18	Ribosomal Protein S18	Protein Coding	38	GC06P083759	0.172717214
SF3B3	Splicing Factor 3b Subunit 3	Protein Coding	38	GC16P070523	0.172717214
SRSF7	Serine And Arginine Rich Splicing Factor 7	Protein Coding	38	GC02M038743	0.172717214
CA7	Carbonic Anhydrase 7	Protein Coding	37	GC16P066844	0.172717214
CACYBP	Calcyclin Binding Protein	Protein Coding	37	GC01P175001	0.172717214
CIRBP	Cold Inducible RNA Binding Protein	Protein Coding	37	GC19P001259	0.172717214
HNRNPH2	Heterogeneous Nuclear Ribonucleoprotein H2	Protein Coding	37	GC0XP101408	0.172717214
IST1	IST1 Factor Associated With ESCRT-III	Protein Coding	37	GC16P072066	0.172717214
RBM14	RNA Binding Motif Protein 14	Protein Coding	37	GC11P070099	0.172717214
RBM39	RNA Binding Motif Protein 39	Protein Coding	37	GC20M035703	0.172717214
RPL17	Ribosomal Protein L17	Protein Coding	37	GC18M049488	0.172717214
PABPC3	Poly(A) Binding Protein Cytoplasmic 3	Protein Coding	36	GC13P025445	0.172717214
RPS25	Ribosomal Protein S25	Protein Coding	36	GC11M119015	0.172717214
STK32A	Serine/Threonine Kinase 32A	Protein Coding	36	GC05P147234	0.172717214
TP53INP1	Tumor Protein P53 Inducible Nuclear Protein 1	Protein Coding	36	GC08M094925	0.172717214
EPRS1	Glutamyl-Prolyl-TRNA Synthetase 1	Protein Coding	35	GC01M219969	0.172717214
RACK1	Receptor For Activated C Kinase 1	Protein Coding	35	GC05M181795	0.172717214
SERBP1	SERPINE1 MRNA Binding Protein 1	Protein Coding	35	GC01M067407	0.172717214
TMEM11	Transmembrane Protein 11	Protein Coding	35	GC17M021197	0.172717214
ATG9B	Autophagy Related 9B	Protein Coding	34	GC07M151012	0.172717214
NTPCR	Nucleoside-Triphosphatase, Cancer-Related	Protein Coding	34	GC01P232950	0.172717214
RPL26L1	Ribosomal Protein L26 Like 1	Protein Coding	34	GC05P172958	0.172717214
FAM131B	Family With Sequence Similarity 131 Member B	Protein Coding	33	GC07M143353	0.172717214
H1-0	H1.0 Linker Histone	Protein Coding	31	GC22P039222	0.172717214
H3C1	H3 Clustered Histone 1	Protein Coding	31	GC06P083483	0.172717214
ATP5F1C	ATP Synthase F1 Subunit Gamma	Protein Coding	30	GC10P007789	0.172717214
H3C2	H3 Clustered Histone 2	Protein Coding	29	GC06M026032	0.172717214
H3C3	H3 Clustered Histone 3	Protein Coding	29	GC06P083486	0.172717214
H3C12	H3 Clustered Histone 12	Protein Coding	28	GC06M066514	0.172717214
H3C4	H3 Clustered Histone 4	Protein Coding	28	GC06M066515	0.172717214
H3C10	H3 Clustered Histone 10	Protein Coding	27	GC06P083548	0.172717214
H2AC18	H2A Clustered Histone 18	Protein Coding	26	GC01M152015	0.172717214
H3C11	H3 Clustered Histone 11	Protein Coding	26	GC06M066513	0.172717214
H3C6	H3 Clustered Histone 6	Protein Coding	26	GC06P084429	0.172717214
H3C7	H3 Clustered Histone 7	Protein Coding	26	GC06M066516	0.172717214
H3C8	H3 Clustered Histone 8	Protein Coding	26	GC06M066517	0.172717214
ATR	ATR Serine/Threonine Kinase	Protein Coding	50	GC03M142449	0.14102301
CTSD	Cathepsin D	Protein Coding	50	GC11M001752	0.14102301
PPARG	Peroxisome Proliferator Activated Receptor Gamma	Protein Coding	50	GC03P012287	0.14102301
ATP2A2	ATPase Sarcoplasmic/Endoplasmic Reticulum Ca2+ Transporting 2	Protein Coding	49	GC12P110280	0.14102301
CA2	Carbonic Anhydrase 2	Protein Coding	49	GC08P085463	0.14102301
SCN5A	Sodium Voltage-Gated Channel Alpha Subunit 5	Protein Coding	49	GC03M038549	0.14102301
BRCA1	BRCA1 DNA Repair Associated	Protein Coding	48	GC17M043044	0.14102301

COL1A1	Collagen Type I Alpha 1 Chain	Protein Coding	48	GC17M050183	0.14102301
EPAS1	Endothelial PAS Domain Protein 1	Protein Coding	48	GC02P046293	0.14102301
MSH6	MutS Homolog 6	Protein Coding	48	GC02P047695	0.14102301
PPIB	Peptidylprolyl Isomerase B	Protein Coding	48	GC15M064155	0.14102301
PRKDC	Protein Kinase, DNA-Activated, Catalytic Subunit	Protein Coding	48	GC08M047773	0.14102301
AHCY	Adenosylhomocysteinase	Protein Coding	47	GC20M034827	0.14102301
EEF2	Eukaryotic Translation Elongation Factor 2	Protein Coding	47	GC19M003976	0.14102301
IL6	Interleukin 6	Protein Coding	47	GC07P022725	0.14102301
IRAK1	Interleukin 1 Receptor Associated Kinase 1	Protein Coding	47	GC0XM154010	0.14102301
RPS19	Ribosomal Protein S19	Protein Coding	47	GC19P066764	0.14102301
YWHAG	Tyrosine 3-Monooxygenase/Tryptophan 5-Monooxygenase Activation Protein Gamma	Protein Coding	47	GC07M076839	0.14102301
ACACA	Acetyl-CoA Carboxylase Alpha	Protein Coding	46	GC17M037084	0.14102301
ALDOA	Aldolase, Fructose-Bisphosphate A	Protein Coding	46	GC16P030064	0.14102301
ATP2B3	ATPase Plasma Membrane Ca2+ Transporting 3	Protein Coding	46	GC0XP153517	0.14102301
FLNA	Filamin A	Protein Coding	46	GC0XM154348	0.14102301
FLNB	Filamin B	Protein Coding	46	GC03P058008	0.14102301
GPI	Glucose-6-Phosphate Isomerase	Protein Coding	46	GC19P034359	0.14102301
GSN	Gelsolin	Protein Coding	46	GC09P121201	0.14102301
IL6R	Interleukin 6 Receptor	Protein Coding	46	GC01P154405	0.14102301
P4HB	Prolyl 4-Hydroxylase Subunit Beta	Protein Coding	46	GC17M081843	0.14102301
RANBP2	RAN Binding Protein 2	Protein Coding	46	GC02P108719	0.14102301
RDX	Radixin	Protein Coding	46	GC11M109864	0.14102301
SDHA	Succinate Dehydrogenase Complex Flavoprotein Subunit A	Protein Coding	46	GC05P000208	0.14102301
TPM1	Tropomyosin 1	Protein Coding	46	GC15P120433	0.14102301
TUBB1	Tubulin Beta 1 Class VI	Protein Coding	46	GC20P059020	0.14102301
ACLY	ATP Citrate Lyase	Protein Coding	45	GC17M041866	0.14102301
ACTA2	Actin Alpha 2, Smooth Muscle	Protein Coding	45	GC10M088935	0.14102301
AFG3L2	AFG3 Like Matrix AAA Peptidase Subunit 2	Protein Coding	45	GC18M012328	0.14102301
AKR1B1	Aldo-Keto Reductase Family 1 Member B	Protein Coding	45	GC07M134442	0.14102301
ARHGDIA	Rho GDP Dissociation Inhibitor Alpha	Protein Coding	45	GC17M081867	0.14102301
ASNS	Asparagine Synthetase (Glutamine-Hydrolyzing)	Protein Coding	45	GC07M097854	0.14102301
GANAB	Glucosidase II Alpha Subunit	Protein Coding	45	GC11M089627	0.14102301
GJB1	Gap Junction Protein Beta 1	Protein Coding	45	GC0XP071212	0.14102301
HADHA	Hydroxyacyl-CoA Dehydrogenase Trifunctional Multienzyme Complex Subunit Alpha	Protein Coding	45	GC02M026190	0.14102301
HADHB	Hydroxyacyl-CoA Dehydrogenase Trifunctional Multienzyme Complex Subunit Beta	Protein Coding	45	GC02P026243	0.14102301
LDHB	Lactate Dehydrogenase B	Protein Coding	45	GC12M021635	0.14102301
MCM4	Minichromosome Maintenance Complex Component 4	Protein Coding	45	GC08P047965	0.14102301
MSN	Moesin	Protein Coding	45	GC0XP065588	0.14102301
MYH10	Myosin Heavy Chain 10	Protein Coding	45	GC17M008474	0.14102301
NDUFS3	NADH:Ubiquinone Oxidoreductase Core Subunit S3	Protein Coding	45	GC11P047567	0.14102301
PCCA	Propionyl-CoA Carboxylase Subunit Alpha	Protein Coding	45	GC13P100089	0.14102301
PCCB	Propionyl-CoA Carboxylase Subunit Beta	Protein Coding	45	GC03P136250	0.14102301
PCK2	Phosphoenolpyruvate Carboxykinase 2, Mitochondrial	Protein Coding	45	GC14P024094	0.14102301
PFN1	Profilin 1	Protein Coding	45	GC17M004945	0.14102301
PPIA	Peptidylprolyl Isomerase A	Protein Coding	45	GC07P044807	0.14102301
PRSS1	Serine Protease 1	Protein Coding	45	GC07P148339	0.14102301
PSAT1	Phosphoserine Aminotransferase 1	Protein Coding	45	GC09P078297	0.14102301
RPL5	Ribosomal Protein L5	Protein Coding	45	GC01P092832	0.14102301
TKT	Transketolase	Protein Coding	45	GC03M053224	0.14102301
TPI1	Triosephosphate Isomerase 1	Protein Coding	45	GC12P006867	0.14102301
TPM3	Tropomyosin 3	Protein Coding	45	GC01M154127	0.14102301
UBA1	Ubiquitin Like Modifier Activating Enzyme 1	Protein Coding	45	GC0XP047190	0.14102301
VCL	Vinculin	Protein Coding	45	GC10P073995	0.14102301
ACTG2	Actin Gamma 2, Smooth Muscle	Protein Coding	44	GC02P073892	0.14102301
ARAF	A-Raf Proto-Oncogene, Serine/Threonine Kinase	Protein Coding	44	GC0XP047572	0.14102301
ARF1	ADP Ribosylation Factor 1	Protein Coding	44	GC01P228082	0.14102301
ATIC	5-Aminoimidazole-4-Carboxamide Ribonucleotide Formyltransferase/IMP Cyclohydrolase	Protein Coding	44	GC02P215311	0.14102301
CALM1	Calmodulin 1	Protein Coding	44	GC14P090396	0.14102301
CTPS1	CTP Synthase 1	Protein Coding	44	GC01P040979	0.14102301
DDX6	DEAD-Box Helicase 6	Protein Coding	44	GC11M118748	0.14102301
DLAT	Dihydrolipoamide S-Acetyltransferase	Protein Coding	44	GC11P112026	0.14102301
EEF1A2	Eukaryotic Translation Elongation Factor 1 Alpha 2	Protein Coding	44	GC20M063488	0.14102301
EIF4A1	Eukaryotic Translation Initiation Factor 4A1	Protein Coding	44	GC17P007572	0.14102301
FLNC	Filamin C	Protein Coding	44	GC07P128830	0.14102301
HYOU1	Hypoxia Up-Regulated 1	Protein Coding	44	GC11M119206	0.14102301
MCM3	Minichromosome Maintenance Complex Component 3	Protein Coding	44	GC06M052264	0.14102301
MTNR1B	Melatonin Receptor 1B	Protein Coding	44	GC11P092969	0.14102301
NDUFS1	NADH:Ubiquinone Oxidoreductase Core Subunit S1	Protein Coding	44	GC02M206114	0.14102301
NME1	NME/NM23 Nucleoside Diphosphate Kinase 1	Protein Coding	44	GC17P056083	0.14102301
PABPN1	Poly(A) Binding Protein Nuclear 1	Protein Coding	44	GC14P033039	0.14102301
PARK7	Parkinsonism Associated Deglycase	Protein Coding	44	GC01P008102	0.14102301
PRDX2	Peroxiredoxin 2	Protein Coding	44	GC19M012796	0.14102301
PSMA6	Proteasome 20S Subunit Alpha 6	Protein Coding	44	GC14P035278	0.14102301
SF3B1	Splicing Factor 3b Subunit 1	Protein Coding	44	GC02M197427	0.14102301
SMC1A	Structural Maintenance Of Chromosomes 1A	Protein Coding	44	GC0XM053374	0.14102301
SMC3	Structural Maintenance Of Chromosomes 3	Protein Coding	44	GC10P110567	0.14102301
TUBA4A	Tubulin Alpha 4a	Protein Coding	44	GC02M219249	0.14102301
TUBB4A	Tubulin Beta 4A Class IVa	Protein Coding	44	GC19M006496	0.14102301
YWHAQ	Tyrosine 3-Monooxygenase/Tryptophan 5-Monooxygenase Activation Protein Theta	Protein Coding	44	GC02M009583	0.14102301
C4A	Complement C4A (Rodgers Blood Group)	Protein Coding	43	GC06P083718	0.14102301
C4B	Complement C4B (Chido Blood Group)	Protein Coding	43	GC06P032014	0.14102301
CCT5	Chaperonin Containing TCP1 Subunit 5	Protein Coding	43	GC05P010236	0.14102301
CHD4	Chromodomain Helicase DNA Binding Protein 4	Protein Coding	43	GC12M006570	0.14102301
CHGA	Chromogranin A	Protein Coding	43	GC14P092944	0.14102301
CSTA	Cystatin A	Protein Coding	43	GC03P122325	0.14102301
CUX1	Cut Like Homeobox 1	Protein Coding	43	GC07P101815	0.14102301
DDX41	DEAD-Box Helicase 41	Protein Coding	43	GC05M177511	0.14102301
EZR	Ezrin	Protein Coding	43	GC06M158765	0.14102301
FKBP4	FKBP Prolyl Isomerase 4	Protein Coding	43	GC12P002795	0.14102301
GMPS	Guanine Monophosphate Synthase	Protein Coding	43	GC03P155870	0.14102301
HAND2	Heart And Neural Crest Derivatives Expressed 2	Protein Coding	43	GC04M173524	0.14102301
IGF2BP2	Insulin Like Growth Factor 2 MRNA Binding Protein 2	Protein Coding	43	GC03M185643	0.14102301
NDUFS2	NADH:Ubiquinone Oxidoreductase Core Subunit S2	Protein Coding	43	GC01P161197	0.14102301
NONO	Non-POU Domain Containing Octamer Binding	Protein Coding	43	GC0XP071255	0.14102301
NUMA1	Nuclear Mitotic Apparatus Protein 1	Protein Coding	43	GC11M072002	0.14102301
PKP2	Plakophilin 2	Protein Coding	43	GC12M032790	0.14102301
PRMT5	Protein Arginine Methyltransferase 5	Protein Coding	43	GC14M022920	0.14102301
PSMB1	Proteasome 20S Subunit Beta 1	Protein Coding	43	GC06M170535	0.14102301

RPL18	Ribosomal Protein L18	Protein Coding	43	GC19M048615	0.14102301
RPL35	Ribosomal Protein L35	Protein Coding	43	GC09M124857	0.14102301
STIP1	Stress Induced Phosphoprotein 1	Protein Coding	43	GC11P064382	0.14102301
TRPC4	Transient Receptor Potential Cation Channel Subfamily C Member 4	Protein Coding	43	GC13M037636	0.14102301
ZYX	Zyxin	Protein Coding	43	GC07P143381	0.14102301
ATP2B1	ATPase Plasma Membrane Ca2+ Transporting 1	Protein Coding	42	GC12M089588	0.14102301
DGKZ	Diacylglycerol Kinase Zeta	Protein Coding	42	GC11P046332	0.14102301
DSG1	Desmoglein 1	Protein Coding	42	GC18P031318	0.14102301
EIF4A3	Eukaryotic Translation Initiation Factor 4A3	Protein Coding	42	GC17M080135	0.14102301
FARSB	Phenylalanyl-TRNA Synthetase Subunit Beta	Protein Coding	42	GC02M222570	0.14102301
FXR1	FMR1 Autosomal Homolog 1	Protein Coding	42	GC03P180868	0.14102301
GART	Phosphoribosylglycinamide Formyltransferase, Phosphoribosylglycinamide Synthetase, Phosphoribosylaminoimidazole Synthetase	Protein Coding	42	GC21M033503	0.14102301
GCNT2	Glucosaminyl (N-Acetyl) Transferase 2 (I Blood Group)	Protein Coding	42	GC06P010492	0.14102301
LMNB2	Lamin B2	Protein Coding	42	GC19M005364	0.14102301
MARK2	Microtubule Affinity Regulating Kinase 2	Protein Coding	42	GC11P063838	0.14102301
MCCC2	Methylcrotonyl-CoA Carboxylase Subunit 2	Protein Coding	42	GC05P072983	0.14102301
MCM6	Minichromosome Maintenance Complex Component 6	Protein Coding	42	GC02M135839	0.14102301
NDUFS4	NADH:Ubiquinone Oxidoreductase Subunit S4	Protein Coding	42	GC05P053560	0.14102301
PDCD6IP	Programmed Cell Death 6 Interacting Protein	Protein Coding	42	GC03P033798	0.14102301
PPP1R12A	Protein Phosphatase 1 Regulatory Subunit 12A	Protein Coding	42	GC12M079773	0.14102301
PSMA1	Proteasome 20S Subunit Alpha 1	Protein Coding	42	GC11M014505	0.14102301
PSMA3	Proteasome 20S Subunit Alpha 3	Protein Coding	42	GC14P058244	0.14102301
PSMA4	Proteasome 20S Subunit Alpha 4	Protein Coding	42	GC15P078540	0.14102301
PSMA5	Proteasome 20S Subunit Alpha 5	Protein Coding	42	GC01M109399	0.14102301
RAN	RAN, Member RAS Oncogene Family	Protein Coding	42	GC12P130871	0.14102301
RBMX	RNA Binding Motif Protein X-Linked	Protein Coding	42	GC0XM136848	0.14102301
SLC1A5	Solute Carrier Family 1 Member 5	Protein Coding	42	GC19M065991	0.14102301
SLC25A11	Solute Carrier Family 25 Member 11	Protein Coding	42	GC17M004937	0.14102301
TACC3	Transforming Acidic Coiled-Coil Containing Protein 3	Protein Coding	42	GC04P001723	0.14102301
TRIM32	Tripartite Motif Containing 32	Protein Coding	42	GC09P116687	0.14102301
AGPS	Alkylglycerone Phosphate Synthase	Protein Coding	41	GC02P177392	0.14102301
ARCN1	Archain 1	Protein Coding	41	GC11P118572	0.14102301
ATRIP	ATR Interacting Protein	Protein Coding	41	GC03P048964	0.14102301
CALU	Calumenin	Protein Coding	41	GC07P128739	0.14102301
CCT7	Chaperonin Containing TCP1 Subunit 7	Protein Coding	41	GC02P073233	0.14102301
CDSN	Corneodesmosin	Protein Coding	41	GC06M031115	0.14102301
CHGB	Chromogranin B	Protein Coding	41	GC20P005911	0.14102301
CTTN	Cortactin	Protein Coding	41	GC11P070398	0.14102301
DBT	Dihydrolipoamide Branched Chain Transacylase E2	Protein Coding	41	GC01M100186	0.14102301
DIS3	DIS3 Homolog, Exosome Endoribonuclease And 3'-5' Exoribonuclease	Protein Coding	41	GC13M072752	0.14102301
DROSHA	Drosha Ribonuclease III	Protein Coding	41	GC05M031401	0.14102301
EFTUD2	Elongation Factor Tu GTP Binding Domain Containing 2	Protein Coding	41	GC17M044952	0.14102301
EIF3A	Eukaryotic Translation Initiation Factor 3 Subunit A	Protein Coding	41	GC10M119034	0.14102301
FABP5	Fatty Acid Binding Protein 5	Protein Coding	41	GC08P081282	0.14102301
FANCG	FA Complementation Group G	Protein Coding	41	GC09M035073	0.14102301
IQGAP1	IQ Motif Containing GTPase Activating Protein 1	Protein Coding	41	GC15P090388	0.14102301
KHDRBS1	KH RNA Binding Domain Containing, Signal Transduction Associated 1	Protein Coding	41	GC01P032013	0.14102301
KIF1C	Kinesin Family Member 1C	Protein Coding	41	GC17P005022	0.14102301
LARP7	La Ribonucleoprotein 7, Transcriptional Regulator	Protein Coding	41	GC04P112636	0.14102301
MRPS22	Mitochondrial Ribosomal Protein S22	Protein Coding	41	GC03P139005	0.14102301
MYO1C	Myosin IC	Protein Coding	41	GC17M001464	0.14102301
NPC2	NPC Intracellular Cholesterol Transporter 2	Protein Coding	41	GC14M074476	0.14102301
PAICS	Phosphoribosylaminoimidazole Carboxylase And Phosphoribosylaminoimidazolesuccinocarboxamide Synthase	Protein Coding	41	GC04P056410	0.14102301
PCM1	Pericentriolar Material 1	Protein Coding	41	GC08P017922	0.14102301
PLS3	Plastin 3	Protein Coding	41	GC0XP115560	0.14102301
PSMA2	Proteasome 20S Subunit Alpha 2	Protein Coding	41	GC07M042916	0.14102301
PSMB6	Proteasome 20S Subunit Beta 6	Protein Coding	41	GC17P004796	0.14102301
PSMC5	Proteasome 26S Subunit, ATPase 5	Protein Coding	41	GC17P063827	0.14102301
RPL19	Ribosomal Protein L19	Protein Coding	41	GC17P039200	0.14102301
RPL26	Ribosomal Protein L26	Protein Coding	41	GC17M008377	0.14102301
RPL27	Ribosomal Protein L27	Protein Coding	41	GC17P042998	0.14102301
RPS9	Ribosomal Protein S9	Protein Coding	41	GC19P069272	0.14102301
S100A8	S100 Calcium Binding Protein A8	Protein Coding	41	GC01M153391	0.14102301
S100A9	S100 Calcium Binding Protein A9	Protein Coding	41	GC01P153357	0.14102301
SSB	Small RNA Binding Exonuclease Protection Factor La	Protein Coding	41	GC02P169791	0.14102301
TRIM28	Tripartite Motif Containing 28	Protein Coding	41	GC19P058544	0.14102301
AGK	Acylglycerol Kinase	Protein Coding	40	GC07P141551	0.14102301
ANXA6	Annexin A6	Protein Coding	40	GC05M151100	0.14102301
ASPSCR1	ASPSCR1 Tether For SLC2A4, UBX Domain Containing	Protein Coding	40	GC17P081976	0.14102301
BCLAF1	BCL2 Associated Transcription Factor 1	Protein Coding	40	GC06M136256	0.14102301
CBX1	Chromobox 1	Protein Coding	40	GC17M048070	0.14102301
CBX3	Chromobox 3	Protein Coding	40	GC07P026201	0.14102301
CEP55	Centrosomal Protein 55	Protein Coding	40	GC10P093496	0.14102301
CLPX	Caseinolytic Mitochondrial Matrix Peptidase Chaperone Subunit X	Protein Coding	40	GC15M065148	0.14102301
DYNC1I2	Dynein Cytoplasmic 1 Intermediate Chain 2	Protein Coding	40	GC02P171687	0.14102301
ELAVL1	ELAV Like RNA Binding Protein 1	Protein Coding	40	GC19M007958	0.14102301
FLG	Filaggrin	Protein Coding	40	GC01M152274	0.14102301
IARS2	Isoleucyl-TRNA Synthetase 2, Mitochondrial	Protein Coding	40	GC01P220094	0.14102301
IGF2BP3	Insulin Like Growth Factor 2 MRNA Binding Protein 3	Protein Coding	40	GC07M023316	0.14102301
KHSRP	KH-Type Splicing Regulatory Protein	Protein Coding	40	GC19M006413	0.14102301
MRPS28	Mitochondrial Ribosomal Protein S28	Protein Coding	40	GC08M079942	0.14102301
NUP210	Nucleoporin 210	Protein Coding	40	GC03M020709	0.14102301
PA2G4	Proliferation-Associated 2G4	Protein Coding	40	GC12P057262	0.14102301
PABPC4	Poly(A) Binding Protein Cytoplasmic 4	Protein Coding	40	GC01M039560	0.14102301
PRPF4B	Pre-MRNA Processing Factor 4B	Protein Coding	40	GC06P004021	0.14102301
PRPF6	Pre-MRNA Processing Factor 6	Protein Coding	40	GC20P063981	0.14102301
PSMB2	Proteasome 20S Subunit Beta 2	Protein Coding	40	GC01M035599	0.14102301
PSMB3	Proteasome 20S Subunit Beta 3	Protein Coding	40	GC17P038752	0.14102301
RBBP7	RB Binding Protein 7, Chromatin Remodeling Factor	Protein Coding	40	GC0XM016839	0.14102301
RBM8A	RNA Binding Motif Protein 8A	Protein Coding	40	GC01M145921	0.14102301
RFC4	Replication Factor C Subunit 4	Protein Coding	40	GC03M186789	0.14102301
RNPEP	Arginyl Aminopeptidase	Protein Coding	40	GC01P201982	0.14102301
RPL27A	Ribosomal Protein L27a	Protein Coding	40	GC11P008682	0.14102301
RPL4	Ribosomal Protein L4	Protein Coding	40	GC15M066498	0.14102301
RPL9	Ribosomal Protein L9	Protein Coding	40	GC04M039452	0.14102301
RPN2	Ribophorin II	Protein Coding	40	GC20P037178	0.14102301
RPS7	Ribosomal Protein S7	Protein Coding	40	GC02P003575	0.14102301
SF3B2	Splicing Factor 3b Subunit 2	Protein Coding	40	GC11P066050	0.14102301

SMC4	Structural Maintenance Of Chromosomes 4	Protein Coding	40	GC03P160399	0.14102301
SND1	Staphylococcal Nuclease And Tudor Domain Containing 1	Protein Coding	40	GC07P127652	0.14102301
SPAG9	Sperm Associated Antigen 9	Protein Coding	40	GC17M050962	0.14102301
SRP72	Signal Recognition Particle 72	Protein Coding	40	GC04P056466	0.14102301
SRSF1	Serine And Arginine Rich Splicing Factor 1	Protein Coding	40	GC17M058000	0.14102301
SRSF2	Serine And Arginine Rich Splicing Factor 2	Protein Coding	40	GC17M076734	0.14102301
TLN1	Talin 1	Protein Coding	40	GC09M035696	0.14102301
UBA5	Ubiquitin Like Modifier Activating Enzyme 5	Protein Coding	40	GC03P132654	0.14102301
UBE2V1	Ubiquitin Conjugating Enzyme E2 V1	Protein Coding	40	GC20M050082	0.14102301
UBE4B	Ubiquitination Factor E4B	Protein Coding	40	GC01P010032	0.14102301
UCHL5	Ubiquitin C-Terminal Hydrolase L5	Protein Coding	40	GC01M193012	0.14102301
ACSL3	Acyl-CoA Synthetase Long Chain Family Member 3	Protein Coding	39	GC02P222860	0.14102301
AMY2B	Amylase Alpha 2B	Protein Coding	39	GC01P103554	0.14102301
CALML3	Calmodulin Like 3	Protein Coding	39	GC10P005556	0.14102301
CCT8	Chaperonin Containing TCP1 Subunit 8	Protein Coding	39	GC21M029055	0.14102301
CSE1L	Chromosome Segregation 1 Like	Protein Coding	39	GC20P049046	0.14102301
DAP3	Death Associated Protein 3	Protein Coding	39	GC01P155794	0.14102301
DHX16	DEAH-Box Helicase 16	Protein Coding	39	GC06M030653	0.14102301
GAN	Gigaxonin	Protein Coding	39	GC16P081319	0.14102301
GSPT1	G1 To S Phase Transition 1	Protein Coding	39	GC16M011868	0.14102301
LCN1	Lipocalin 1	Protein Coding	39	GC09P135521	0.14102301
MAGED2	MAGE Family Member D2	Protein Coding	39	GC0XP054807	0.14102301
MOV10	Mov10 RISC Complex RNA Helicase	Protein Coding	39	GC01P112673	0.14102301
MRE11	MRE11 Homolog, Double Strand Break Repair Nuclease	Protein Coding	39	GC11M096553	0.14102301
MRPL12	Mitochondrial Ribosomal Protein L12	Protein Coding	39	GC17P081704	0.14102301
RAB5C	RAB5C, Member RAS Oncogene Family	Protein Coding	39	GC17M042124	0.14102301
RPL24	Ribosomal Protein L24	Protein Coding	39	GC03M101681	0.14102301
S100A7	S100 Calcium Binding Protein A7	Protein Coding	39	GC01M153457	0.14102301
SUPT16H	SPT16 Homolog, Facilitates Chromatin Remodeling Subunit	Protein Coding	39	GC14M021351	0.14102301
ZC3H14	Zinc Finger CCCH-Type Containing 14	Protein Coding	39	GC14P088562	0.14102301
CDC40	Cell Division Cycle 40	Protein Coding	38	GC06P110180	0.14102301
DDX21	DExD-Box Helicase 21	Protein Coding	38	GC10P068956	0.14102301
DDX47	DEAD-Box Helicase 47	Protein Coding	38	GC12P021345	0.14102301
DSC1	Desmocollin 1	Protein Coding	38	GC18M031129	0.14102301
EEF1G	Eukaryotic Translation Elongation Factor 1 Gamma	Protein Coding	38	GC11M089615	0.14102301
EIF3B	Eukaryotic Translation Initiation Factor 3 Subunit B	Protein Coding	38	GC07P002354	0.14102301
EIF3L	Eukaryotic Translation Initiation Factor 3 Subunit L	Protein Coding	38	GC22P037848	0.14102301
FUBP1	Far Upstream Element Binding Protein 1	Protein Coding	38	GC01M077944	0.14102301
HNRNPH3	Heterogeneous Nuclear Ribonucleoprotein H3	Protein Coding	38	GC10P068331	0.14102301
HNRNPL	Heterogeneous Nuclear Ribonucleoprotein L	Protein Coding	38	GC19M038836	0.14102301
ILF3	Interleukin Enhancer Binding Factor 3	Protein Coding	38	GC19P010655	0.14102301
INA	Internexin Neuronal Intermediate Filament Protein Alpha	Protein Coding	38	GC10P103277	0.14102301
KLHL9	Kelch Like Family Member 9	Protein Coding	38	GC09M021329	0.14102301
LARP1	La Ribonucleoprotein 1, Translational Regulator	Protein Coding	38	GC05P154682	0.14102301
LIMA1	LIM Domain And Actin Binding 1	Protein Coding	38	GC12M050175	0.14102301
MRPS23	Mitochondrial Ribosomal Protein S23	Protein Coding	38	GC17M057834	0.14102301
MTA2	Metastasis Associated 1 Family Member 2	Protein Coding	38	GC11M089621	0.14102301
MYL12A	Myosin Light Chain 12A	Protein Coding	38	GC18P003238	0.14102301
NPLOC4	NPL4 Homolog, Ubiquitin Recognition Factor	Protein Coding	38	GC17M081556	0.14102301
PFN2	Profilin 2	Protein Coding	38	GC03M149964	0.14102301
PIP	Prolactin Induced Protein	Protein Coding	38	GC07P143132	0.14102301
RPL29	Ribosomal Protein L29	Protein Coding	38	GC03M052033	0.14102301
SARM1	Sterile Alpha And TIR Motif Containing 1	Protein Coding	38	GC17P028364	0.14102301
SFXN1	Sideroflexin 1	Protein Coding	38	GC05P175477	0.14102301
SPECC1L	Sperm Antigen With Calponin Homology And Coiled-Coil Domains 1 Like	Protein Coding	38	GC22P036098	0.14102301
SRSF3	Serine And Arginine Rich Splicing Factor 3	Protein Coding	38	GC06P083846	0.14102301
SRSF9	Serine And Arginine Rich Splicing Factor 9	Protein Coding	38	GC12M120461	0.14102301
TPM4	Tropomyosin 4	Protein Coding	38	GC19P066197	0.14102301
UBE2O	Ubiquitin Conjugating Enzyme E2 O	Protein Coding	38	GC17M076389	0.14102301
UFM1	Ubiquitin Fold Modifier 1	Protein Coding	38	GC13P038349	0.14102301
UFSP2	UFM1 Specific Peptidase 2	Protein Coding	38	GC04M185399	0.14102301
VGF	VGF Nerve Growth Factor Inducible	Protein Coding	38	GC07M101162	0.14102301
AHNAK	AHNAK Nucleoprotein	Protein Coding	37	GC11M089610	0.14102301
ATXN2L	Ataxin 2 Like	Protein Coding	37	GC16P040917	0.14102301
CGN	Cingulin	Protein Coding	37	GC01P151483	0.14102301
COX6A2	Cytochrome C Oxidase Subunit 6A2	Protein Coding	37	GC16M037237	0.14102301
DHX15	DEAH-Box Helicase 15	Protein Coding	37	GC04M024519	0.14102301
DHX8	DEAH-Box Helicase 8	Protein Coding	37	GC17P043483	0.14102301
DNAJC7	DnaJ Heat Shock Protein Family (Hsp40) Member C7	Protein Coding	37	GC17M042002	0.14102301
ECD	Ecdysoneless Cell Cycle Regulator	Protein Coding	37	GC10M073130	0.14102301
EIF5B	Eukaryotic Translation Initiation Factor 5B	Protein Coding	37	GC02P099320	0.14102301
FHOD1	Formin Homology 2 Domain Containing 1	Protein Coding	37	GC16M067230	0.14102301
FLG2	Filaggrin 2	Protein Coding	37	GC01M152321	0.14102301
GATAD2A	GATA Zinc Finger Domain Containing 2A	Protein Coding	37	GC19P066252	0.14102301
GGCT	Gamma-Glutamylcyclotransferase	Protein Coding	37	GC07M030496	0.14102301
IFT74	Intraflagellar Transport 74	Protein Coding	37	GC09P026947	0.14102301
KARS1	Lysyl-TRNA Synthetase 1	Protein Coding	37	GC16M075750	0.14102301
KCTD3	Potassium Channel Tetramerization Domain Containing 3	Protein Coding	37	GC01P215567	0.14102301
LARS1	Leucyl-TRNA Synthetase 1	Protein Coding	37	GC05M146114	0.14102301
MARCKSL1	MARCKS Like 1	Protein Coding	37	GC01M032334	0.14102301
MPRIP	Myosin Phosphatase Rho Interacting Protein	Protein Coding	37	GC17P017042	0.14102301
MTCH1	Mitochondrial Carrier 1	Protein Coding	37	GC06M066118	0.14102301
MYO1B	Myosin IB	Protein Coding	37	GC02P191246	0.14102301
RALY	RALY Heterogeneous Nuclear Ribonucleoprotein	Protein Coding	37	GC20P033993	0.14102301
RBM15	RNA Binding Motif Protein 15	Protein Coding	37	GC01P110338	0.14102301
SF3A1	Splicing Factor 3a Subunit 1	Protein Coding	37	GC22M030331	0.14102301
SHROOM3	Shroom Family Member 3	Protein Coding	37	GC04P076435	0.14102301
SRP68	Signal Recognition Particle 68	Protein Coding	37	GC17M076038	0.14102301
SRRM1	Serine And Arginine Repetitive Matrix 1	Protein Coding	37	GC01P024631	0.14102301
ST13	ST13 Hsp70 Interacting Protein	Protein Coding	37	GC22M057601	0.14102301
TBC1D9	TBC1 Domain Family Member 9	Protein Coding	37	GC04M140621	0.14102301
TCF25	Transcription Factor 25	Protein Coding	37	GC16P089873	0.14102301
THRAP3	Thyroid Hormone Receptor Associated Protein 3	Protein Coding	37	GC01P036224	0.14102301
YBX1	Y-Box Binding Protein 1	Protein Coding	37	GC01P042682	0.14102301
YBX3	Y-Box Binding Protein 3	Protein Coding	37	GC12M021116	0.14102301
APOO	Apolipoprotein O	Protein Coding	36	GC0XM023834	0.14102301
CEBPZ	CCAAT Enhancer Binding Protein Zeta	Protein Coding	36	GC02M037201	0.14102301
CILP2	Cartilage Intermediate Layer Protein 2	Protein Coding	36	GC19P019538	0.14102301

COL5A3	Collagen Type V Alpha 3 Chain	Protein Coding	36	GC19M009931	0.14102301
FUBP3	Far Upstream Element Binding Protein 3	Protein Coding	36	GC09P130580	0.14102301
GARS1	Glycyl-TRNA Synthetase 1	Protein Coding	36	GC07P030580	0.14102301
HTATSF1	HIV-1 Tat Specific Factor 1	Protein Coding	36	GC0XP136497	0.14102301
IRF2BP2	Interferon Regulatory Factor 2 Binding Protein 2	Protein Coding	36	GC01M234604	0.14102301
LGALS7	Galectin 7	Protein Coding	36	GC19M038770	0.14102301
MRPS27	Mitochondrial Ribosomal Protein S27	Protein Coding	36	GC05M073589	0.14102301
MRPS31	Mitochondrial Ribosomal Protein S31	Protein Coding	36	GC13M040729	0.14102301
NCDN	Neurochondrin	Protein Coding	36	GC01P035557	0.14102301
NKTR	Natural Killer Cell Triggering Receptor	Protein Coding	36	GC03P042600	0.14102301
NOP2	NOP2 Nucleolar Protein	Protein Coding	36	GC12M006556	0.14102301
PPFIA3	PTPRF Interacting Protein Alpha 3	Protein Coding	36	GC19P049119	0.14102301
PRPF19	Pre-MRNA Processing Factor 19	Protein Coding	36	GC11M060890	0.14102301
PRPF40A	Pre-MRNA Processing Factor 40 Homolog A	Protein Coding	36	GC02M152651	0.14102301
SHKBP1	SH3KBP1 Binding Protein 1	Protein Coding	36	GC19P040576	0.14102301
SMPDL3B	Sphingomyelin Phosphodiesterase Acid Like 3B	Protein Coding	36	GC01P028207	0.14102301
SRRM2	Serine/Arginine Repetitive Matrix 2	Protein Coding	36	GC16P011722	0.14102301
TIMD4	T Cell Immunoglobulin And Mucin Domain Containing 4	Protein Coding	36	GC05M156919	0.14102301
UFC1	Ubiquitin-Fold Modifier Conjugating Enzyme 1	Protein Coding	36	GC01P161152	0.14102301
VAT1	Vesicle Amine Transport 1	Protein Coding	36	GC17M043014	0.14102301
WRNIP1	WRN Helicase Interacting Protein 1	Protein Coding	36	GC06P002766	0.14102301
ZNF326	Zinc Finger Protein 326	Protein Coding	36	GC01P089995	0.14102301
DNAJC11	DnaJ Heat Shock Protein Family (Hsp40) Member C11	Protein Coding	35	GC01M006634	0.14102301
FBXL2	F-Box And Leucine Rich Repeat Protein 2	Protein Coding	35	GC03P033277	0.14102301
GSDMA	Gasdermin A	Protein Coding	35	GC17P055533	0.14102301
IARS1	Isoleucyl-TRNA Synthetase 1	Protein Coding	35	GC09M094184	0.14102301
LIMCH1	LIM And Calponin Homology Domains 1	Protein Coding	35	GC04P041362	0.14102301
LUZP1	Leucine Zipper Protein 1	Protein Coding	35	GC01M023085	0.14102301
MRPS18B	Mitochondrial Ribosomal Protein S18B	Protein Coding	35	GC06P030617	0.14102301
PNN	Pinin, Desmosome Associated Protein	Protein Coding	35	GC14P039175	0.14102301
RARS1	Arginyl-TRNA Synthetase 1	Protein Coding	35	GC05P168487	0.14102301
U2SURP	U2 SnRNP Associated SURP Domain Containing	Protein Coding	35	GC03P142964	0.14102301
VARS1	Valyl-TRNA Synthetase 1	Protein Coding	35	GC06M065973	0.14102301
ARMC1	Armadillo Repeat Containing 1	Protein Coding	34	GC08M065602	0.14102301
CALML5	Calmodulin Like 5	Protein Coding	34	GC10M005498	0.14102301
CASC3	CASC3 Exon Junction Complex Subunit	Protein Coding	34	GC17P040140	0.14102301
FUNDC2	FUN14 Domain Containing 2	Protein Coding	34	GC0XP155025	0.14102301
H1-4	H1.4 Linker Histone, Cluster Member	Protein Coding	34	GC06P084416	0.14102301
HSDL2	Hydroxysteroid Dehydrogenase Like 2	Protein Coding	34	GC09P112379	0.14102301
MAGEE1	MAGE Family Member E1	Protein Coding	34	GC0XP076427	0.14102301
MRPS15	Mitochondrial Ribosomal Protein S15	Protein Coding	34	GC01M036455	0.14102301
RBM25	RNA Binding Motif Protein 25	Protein Coding	34	GC14P073058	0.14102301
RBM27	RNA Binding Motif Protein 27	Protein Coding	34	GC05P146310	0.14102301
SEMG1	Semenogelin 1	Protein Coding	34	GC20P045206	0.14102301
SUGP2	SURP And G-Patch Domain Containing 2	Protein Coding	34	GC19M018990	0.14102301
TUBA3D	Tubulin Alpha 3d	Protein Coding	34	GC02P136917	0.14102301
UFL1	UFM1 Specific Ligase 1	Protein Coding	34	GC06P096521	0.14102301
ZNF746	Zinc Finger Protein 746	Protein Coding	34	GC07M149472	0.14102301
APOOL	Apolipoprotein O Like	Protein Coding	33	GC0XP085003	0.14102301
ATP5PO	ATP Synthase Peripheral Stalk Subunit OSCP	Protein Coding	33	GC21M033904	0.14102301
EPPK1	Epiplakin 1	Protein Coding	33	GC08M143857	0.14102301
IGHG1	Immunoglobulin Heavy Constant Gamma 1 (G1m Marker)	Protein Coding	33	GC14M105736	0.14102301
NUFIP2	Nuclear FMR1 Interacting Protein 2	Protein Coding	33	GC17M035147	0.14102301
PABPC1L	Poly(A) Binding Protein Cytoplasmic 1 Like	Protein Coding	33	GC20P044910	0.14102301
PIWIL3	Piwi Like RNA-Mediated Gene Silencing 3	Protein Coding	33	GC22M037273	0.14102301
PLD6	Phospholipase D Family Member 6	Protein Coding	33	GC17M017297	0.14102301
PRRC2C	Proline Rich Coiled-Coil 2C	Protein Coding	33	GC01P171486	0.14102301
SEPTIN9	Septin 9	Protein Coding	33	GC17P077282	0.14102301
UBAP2	Ubiquitin Associated Protein 2	Protein Coding	33	GC09M033921	0.14102301
ZC3H13	Zinc Finger CCCH-Type Containing 13	Protein Coding	33	GC13M045954	0.14102301
ZC3H3	Zinc Finger CCCH-Type Containing 3	Protein Coding	33	GC08M143437	0.14102301
H1-2	H1.2 Linker Histone, Cluster Member	Protein Coding	32	GC06M026056	0.14102301
MTFR2	Mitochondrial Fission Regulator 2	Protein Coding	32	GC06M136231	0.14102301
SBSN	Suprabasin	Protein Coding	32	GC19M067523	0.14102301
ZC3H15	Zinc Finger CCCH-Type Containing 15	Protein Coding	32	GC02P186486	0.14102301
ZNF804B	Zinc Finger Protein 804B	Protein Coding	32	GC07P088759	0.14102301
MISP	Mitotic Spindle Positioning	Protein Coding	31	GC19P002791	0.14102301
C1orf35	Chromosome 1 Open Reading Frame 35	Protein Coding	30	GC01M228100	0.14102301
H2AC20	H2A Clustered Histone 20	Protein Coding	30	GC01P150156	0.14102301
H2BC21	H2B Clustered Histone 21	Protein Coding	30	GC01M152029	0.14102301
H4C1	H4 Clustered Histone 1	Protein Coding	30	GC06P083484	0.14102301
IGHA1	Immunoglobulin Heavy Constant Alpha 1	Protein Coding	30	GC14M112710	0.14102301
GCN1	GCN1 Activator Of EIF2AK4	Protein Coding	29	GC12M120128	0.14102301
H1-3	H1.3 Linker Histone, Cluster Member	Protein Coding	29	GC06M066504	0.14102301
H2AC11	H2A Clustered Histone 11	Protein Coding	29	GC06P084417	0.14102301
HNRNPCL1	Heterogeneous Nuclear Ribonucleoprotein C Like 1	Protein Coding	29	GC01M012848	0.14102301
ATP5MF	ATP Synthase Membrane Subunit F	Protein Coding	28	GC07M099506	0.14102301
H2AC1	H2A Clustered Histone 1	Protein Coding	28	GC06M025975	0.14102301
H2AC4	H2A Clustered Histone 4	Protein Coding	28	GC06M026034	0.14102301
H2BC5	H2B Clustered Histone 5	Protein Coding	28	GC06P084424	0.14102301
H4C11	H4 Clustered Histone 11	Protein Coding	28	GC06P083549	0.14102301
H2AW	H2A.W Histone	Protein Coding	27	GC01M228551	0.14102301
H2BC12	H2B Clustered Histone 12	Protein Coding	27	GC06M065642	0.14102301
H2BC14	H2B Clustered Histone 14	Protein Coding	27	GC06P083547	0.14102301
H2BC9	H2B Clustered Histone 9	Protein Coding	27	GC06P084427	0.14102301
MICOS13	Mitochondrial Contact Site And Cristae Organizing System Subunit 13	Protein Coding	27	GC19M005692	0.14102301
H2AC12	H2A Clustered Histone 12	Protein Coding	26	GC06P084418	0.14102301
H2AC14	H2A Clustered Histone 14	Protein Coding	26	GC06M065714	0.14102301
H2AC6	H2A Clustered Histone 6	Protein Coding	26	GC06P084419	0.14102301
H2AC7	H2A Clustered Histone 7	Protein Coding	26	GC06M066509	0.14102301
H2AJ	H2A.J Histone	Protein Coding	26	GC12P021406	0.14102301
H2BC11	H2B Clustered Histone 11	Protein Coding	26	GC06M065639	0.14102301
H2BC13	H2B Clustered Histone 13	Protein Coding	26	GC06M065712	0.14102301
H2BC18	H2B Clustered Histone 18	Protein Coding	26	GC01M152006	0.14102301
H2BC3	H2B Clustered Histone 3	Protein Coding	26	GC06M026044	0.14102301
H2BC15	H2B Clustered Histone 15	Protein Coding	25	GC06P083551	0.14102301
H2BC17	H2B Clustered Histone 17	Protein Coding	25	GC06P083558	0.14102301
H2BU1	H2B.U Histone 1	Protein Coding	25	GC01P229321	0.14102301

RADX	RPA1 Related Single Stranded DNA Binding Protein, X-Linked	Protein Coding	25	GC0XP106612	0.14102301
TERB1	Telomere Repeat Binding Bouquet Formation Protein 1	Protein Coding	25	GC16M066755	0.14102301
HSPA7	Heat Shock Protein Family A (Hsp70) Member 7 (Pseudogene)	Pseudogene	24	GC01P161606	0.14102301
ANXA2P2	Annexin A2 Pseudogene 2	Pseudogene	23	GC09P047082	0.14102301
H2BC12L	H2B Clustered Histone 12 Like	Protein Coding	23	GC21P043569	0.14102301
NME2P1	NME2 Pseudogene 1	Pseudogene	22	GC12P120282	0.14102301
RPS26P11	Ribosomal Protein S26 Pseudogene 11	Pseudogene	20	GC0XP072044	0.14102301
PALM2AKAP2	PALM2 And AKAP2 Fusion	Protein Coding	19	GC09P109499	0.14102301
ST13P4	ST13, Hsp70 Interacting Protein Pseudogene 4	Pseudogene	18	GC13P050172	0.14102301
TUBB7P	Tubulin Beta 7 Pseudogene	Pseudogene	16	GC04M189982	0.14102301
IGKV2-30	Immunoglobulin Kappa Variable 2-30	Protein Coding	14	GC02M090921	0.14102301
CALM1P1	Calmodulin 1 Pseudogene 1	Pseudogene	7	GC0XP095499	0.14102301
ERBB2	Erb-B2 Receptor Tyrosine Kinase 2	Protein Coding	52	GC17P039687	0.099718325
FGFR1	Fibroblast Growth Factor Receptor 1	Protein Coding	52	GC08M038400	0.099718325
FGFR2	Fibroblast Growth Factor Receptor 2	Protein Coding	52	GC10M121478	0.099718325
AKT2	AKT Serine/Threonine Kinase 2	Protein Coding	51	GC19M040230	0.099718325
AKT3	AKT Serine/Threonine Kinase 3	Protein Coding	51	GC01M243488	0.099718325
EGFR	Epidermal Growth Factor Receptor	Protein Coding	51	GC07P055019	0.099718325
MET	MET Proto-Oncogene, Receptor Tyrosine Kinase	Protein Coding	51	GC07P116672	0.099718325
RET	Ret Proto-Oncogene	Protein Coding	51	GC10P043182	0.099718325
EPHB4	EPH Receptor B4	Protein Coding	50	GC07M101954	0.099718325
IKBKB	Inhibitor Of Nuclear Factor Kappa B Kinase Subunit Beta	Protein Coding	50	GC08P042271	0.099718325
RPS6KA3	Ribosomal Protein S6 Kinase A3	Protein Coding	50	GC0XM020149	0.099718325
ACVR1	Activin A Receptor Type 1	Protein Coding	49	GC02M157736	0.099718325
CAT	Catalase	Protein Coding	49	GC11P034460	0.099718325
EPHB2	EPH Receptor B2	Protein Coding	49	GC01P022710	0.099718325
MMP13	Matrix Metallopeptidase 13	Protein Coding	49	GC11M102942	0.099718325
NOS3	Nitric Oxide Synthase 3	Protein Coding	49	GC07P150990	0.099718325
PRKCG	Protein Kinase C Gamma	Protein Coding	49	GC19P053879	0.099718325
ACVR2B	Activin A Receptor Type 2B	Protein Coding	48	GC03P038453	0.099718325
BLK	BLK Proto-Oncogene, Src Family Tyrosine Kinase	Protein Coding	48	GC08P011486	0.099718325
BUB1B	BUB1 Mitotic Checkpoint Serine/Threonine Kinase B	Protein Coding	48	GC15P040161	0.099718325
FN1	Fibronectin 1	Protein Coding	48	GC02M215360	0.099718325
LIMK1	LIM Domain Kinase 1	Protein Coding	48	GC07P074082	0.099718325
MARK3	Microtubule Affinity Regulating Kinase 3	Protein Coding	48	GC14P103385	0.099718325
PHGDH	Phosphoglycerate Dehydrogenase	Protein Coding	48	GC01P119660	0.099718325
PRKCA	Protein Kinase C Alpha	Protein Coding	48	GC17P066302	0.099718325
SOD2	Superoxide Dismutase 2	Protein Coding	48	GC06M159669	0.099718325
STAT6	Signal Transducer And Activator Of Transcription 6	Protein Coding	48	GC12M057095	0.099718325
BUB1	BUB1 Mitotic Checkpoint Serine/Threonine Kinase	Protein Coding	47	GC02M110637	0.099718325
CASP3	Caspase 3	Protein Coding	47	GC04M184627	0.099718325
GSK3B	Glycogen Synthase Kinase 3 Beta	Protein Coding	47	GC03M119821	0.099718325
PDE4D	Phosphodiesterase 4D	Protein Coding	47	GC05M058969	0.099718325
PDGFB	Platelet Derived Growth Factor Subunit B	Protein Coding	47	GC22M058085	0.099718325
PIM1	Pim-1 Proto-Oncogene, Serine/Threonine Kinase	Protein Coding	47	GC06P083862	0.099718325
PRKACB	Protein Kinase CAMP-Activated Catalytic Subunit Beta	Protein Coding	47	GC01P084078	0.099718325
SNAP25	Synaptosome Associated Protein 25	Protein Coding	47	GC20P010186	0.099718325
ACTA1	Actin Alpha 1, Skeletal Muscle	Protein Coding	46	GC01M229515	0.099718325
HDAC5	Histone Deacetylase 5	Protein Coding	46	GC17M044076	0.099718325
KRT18	Keratin 18	Protein Coding	46	GC12P052948	0.099718325
MITF	Melanocyte Inducing Transcription Factor	Protein Coding	46	GC03P069788	0.099718325
MMP8	Matrix Metallopeptidase 8	Protein Coding	46	GC11M102617	0.099718325
PLA2G6	Phospholipase A2 Group VI	Protein Coding	46	GC22M058815	0.099718325
PLD2	Phospholipase D2	Protein Coding	46	GC17P004808	0.099718325
PRMT1	Protein Arginine Methyltransferase 1	Protein Coding	46	GC19P049675	0.099718325
RFC1	Replication Factor C Subunit 1	Protein Coding	46	GC04M039291	0.099718325
TAB2	TGF-Beta Activated Kinase 1 (MAP3K7) Binding Protein 2	Protein Coding	46	GC06P149218	0.099718325
TUBG1	Tubulin Gamma 1	Protein Coding	46	GC17P042609	0.099718325
ACO2	Aconitase 2	Protein Coding	45	GC22P041559	0.099718325
BCKDK	Branched Chain Keto Acid Dehydrogenase Kinase	Protein Coding	45	GC16P041121	0.099718325
BIRC5	Baculoviral IAP Repeat Containing 5	Protein Coding	45	GC17P078214	0.099718325
CSNK1E	Casein Kinase 1 Epsilon	Protein Coding	45	GC22M057751	0.099718325
EIF4G1	Eukaryotic Translation Initiation Factor 4 Gamma 1	Protein Coding	45	GC03P184314	0.099718325
EPHB3	EPH Receptor B3	Protein Coding	45	GC03P184561	0.099718325
FGF10	Fibroblast Growth Factor 10	Protein Coding	45	GC05M044340	0.099718325
HADH	Hydroxyacyl-CoA Dehydrogenase	Protein Coding	45	GC04P107989	0.099718325
HMGCS2	3-Hydroxy-3-Methylglutaryl-CoA Synthase 2	Protein Coding	45	GC01M119747	0.099718325
IL10	Interleukin 10	Protein Coding	45	GC01M206767	0.099718325
LIMK2	LIM Domain Kinase 2	Protein Coding	45	GC22P031212	0.099718325
NEDD4	NEDD4 E3 Ubiquitin Protein Ligase	Protein Coding	45	GC15M055826	0.099718325
PPP1CB	Protein Phosphatase 1 Catalytic Subunit Beta	Protein Coding	45	GC02P028752	0.099718325
PRKAB1	Protein Kinase AMP-Activated Non-Catalytic Subunit Beta 1	Protein Coding	45	GC12P119632	0.099718325
PRKCB	Protein Kinase C Beta	Protein Coding	45	GC16P024288	0.099718325
RALA	RAS Like Proto-Oncogene A	Protein Coding	45	GC07P039622	0.099718325
RORB	RAR Related Orphan Receptor B	Protein Coding	45	GC09P074497	0.099718325
SLC18A2	Solute Carrier Family 18 Member A2	Protein Coding	45	GC10P117241	0.099718325
TP73	Tumor Protein P73	Protein Coding	45	GC01P003652	0.099718325
CDK10	Cyclin Dependent Kinase 10	Protein Coding	44	GC16P089680	0.099718325
CYB5R3	Cytochrome B5 Reductase 3	Protein Coding	44	GC22M057611	0.099718325
IL4	Interleukin 4	Protein Coding	44	GC05P132673	0.099718325
KLF4	Kruppel Like Factor 4	Protein Coding	44	GC09M107484	0.099718325
MAP2K6	Mitogen-Activated Protein Kinase Kinase 6	Protein Coding	44	GC17P069414	0.099718325
MEF2D	Myocyte Enhancer Factor 2D	Protein Coding	44	GC01M156463	0.099718325
MUTYH	MutY DNA Glycosylase	Protein Coding	44	GC01M045329	0.099718325
NFIA	Nuclear Factor I A	Protein Coding	44	GC01P060865	0.099718325
NUMB	NUMB Endocytic Adaptor Protein	Protein Coding	44	GC14M073275	0.099718325
PAFAH1B1	Platelet Activating Factor Acetylhydrolase 1b Regulatory Subunit 1	Protein Coding	44	GC17P002593	0.099718325
PRKAB2	Protein Kinase AMP-Activated Non-Catalytic Subunit Beta 2	Protein Coding	44	GC01M147155	0.099718325
S100B	S100 Calcium Binding Protein B	Protein Coding	44	GC21M050764	0.099718325
SHMT1	Serine Hydroxymethyltransferase 1	Protein Coding	44	GC17M025713	0.099718325
SHMT2	Serine Hydroxymethyltransferase 2	Protein Coding	44	GC12P057229	0.099718325
SPRY2	Sprouty RTK Signaling Antagonist 2	Protein Coding	44	GC13M080335	0.099718325
TEC	Tec Protein Tyrosine Kinase	Protein Coding	44	GC04M048150	0.099718325
TUBB2A	Tubulin Beta 2A Class IIa	Protein Coding	44	GC06M003153	0.099718325
UROD	Uroporphyrinogen Decarboxylase	Protein Coding	44	GC01P045353	0.099718325
BAD	BCL2 Associated Agonist Of Cell Death	Protein Coding	43	GC11M089708	0.099718325
CASQ2	Calsequestrin 2	Protein Coding	43	GC01M115700	0.099718325
EIF4A2	Eukaryotic Translation Initiation Factor 4A2	Protein Coding	43	GC03P186783	0.099718325

NEK1	NIMA Related Kinase 1	Protein Coding	43	GC04M169393	0.099718325
PARP2	Poly(ADP-Ribose) Polymerase 2	Protein Coding	43	GC14P020343	0.099718325
PSMB4	Proteasome 20S Subunit Beta 4	Protein Coding	43	GC01P151372	0.099718325
PTPRN	Protein Tyrosine Phosphatase Receptor Type N	Protein Coding	43	GC02M219289	0.099718325
ACSL1	Acyl-CoA Synthetase Long Chain Family Member 1	Protein Coding	42	GC04M184755	0.099718325
ACTC1	Actin Alpha Cardiac Muscle 1	Protein Coding	42	GC15M034790	0.099718325
AIMP1	Aminoacyl TRNA Synthetase Complex Interacting Multifunctional Protein 1	Protein Coding	42	GC04P106315	0.099718325
ASPH	Aspartate Beta-Hydroxylase	Protein Coding	42	GC08M061500	0.099718325
CAMK1	Calcium/Calmodulin Dependent Protein Kinase I	Protein Coding	42	GC03M009774	0.099718325
CAPN5	Calpain 5	Protein Coding	42	GC11P077066	0.099718325
CDC20	Cell Division Cycle 20	Protein Coding	42	GC01P043358	0.099718325
CHMP2B	Charged Multivesicular Body Protein 2B	Protein Coding	42	GC03P087227	0.099718325
CLOCK	Clock Circadian Regulator	Protein Coding	42	GC04M055427	0.099718325
CUL1	Cullin 1	Protein Coding	42	GC07P148697	0.099718325
CXCL12	C-X-C Motif Chemokine Ligand 12	Protein Coding	42	GC10M044370	0.099718325
GRK6	G Protein-Coupled Receptor Kinase 6	Protein Coding	42	GC05P177403	0.099718325
INPP5K	Inositol Polyphosphate-5-Phosphatase K	Protein Coding	42	GC17M001494	0.099718325
KPNB1	Karyopherin Subunit Beta 1	Protein Coding	42	GC17P047649	0.099718325
MAK	Male Germ Cell Associated Kinase	Protein Coding	42	GC06M010762	0.099718325
MAOB	Monoamine Oxidase B	Protein Coding	42	GC0XM043766	0.099718325
MDK	Midkine	Protein Coding	42	GC11P046380	0.099718325
MGST2	Microsomal Glutathione S-Transferase 2	Protein Coding	42	GC04P139665	0.099718325
NANS	N-Acetylneuraminate Synthase	Protein Coding	42	GC09P098056	0.099718325
NOP56	NOP56 Ribonucleoprotein	Protein Coding	42	GC20P004223	0.099718325
NPHP1	Nephrocystin 1	Protein Coding	42	GC02M110122	0.099718325
PAK6	P21 (RAC1) Activated Kinase 6	Protein Coding	42	GC15P040217	0.099718325
PIM2	Pim-2 Proto-Oncogene, Serine/Threonine Kinase	Protein Coding	42	GC0XM048913	0.099718325
PRPF31	Pre-MRNA Processing Factor 31	Protein Coding	42	GC19P067360	0.099718325
PSMC3	Proteasome 26S Subunit, ATPase 3	Protein Coding	42	GC11M089365	0.099718325
PSMD14	Proteasome 26S Subunit, Non-ATPase 14	Protein Coding	42	GC02P161308	0.099718325
PSMD7	Proteasome 26S Subunit, Non-ATPase 7	Protein Coding	42	GC16P074296	0.099718325
RALB	RAS Like Proto-Oncogene B	Protein Coding	42	GC02P120240	0.099718325
RPL15	Ribosomal Protein L15	Protein Coding	42	GC03P023916	0.099718325
SMOC2	SPARC Related Modular Calcium Binding 2	Protein Coding	42	GC06P168441	0.099718325
TECR	Trans-2,3-Enoyl-CoA Reductase	Protein Coding	42	GC19P014504	0.099718325
TFAM	Transcription Factor A, Mitochondrial	Protein Coding	42	GC10P058385	0.099718325
TLE3	TLE Family Member 3, Transcriptional Corepressor	Protein Coding	42	GC15M070047	0.099718325
TOLLIP	Toll Interacting Protein	Protein Coding	42	GC11M001274	0.099718325
USF1	Upstream Transcription Factor 1	Protein Coding	42	GC01M161039	0.099718325
USP10	Ubiquitin Specific Peptidase 10	Protein Coding	42	GC16P084702	0.099718325
BANF1	BAF Nuclear Assembly Factor 1	Protein Coding	41	GC11P066002	0.099718325
CCBE1	Collagen And Calcium Binding EGF Domains 1	Protein Coding	41	GC18M059430	0.099718325
CENPJ	Centromere Protein J	Protein Coding	41	GC13M024882	0.099718325
CHMP4B	Charged Multivesicular Body Protein 4B	Protein Coding	41	GC20P034052	0.099718325
ENDOG	Endonuclease G	Protein Coding	41	GC09P128818	0.099718325
FARSA	Phenylalanyl-TRNA Synthetase Subunit Alpha	Protein Coding	41	GC19M012922	0.099718325
FBL	Fibrillarin	Protein Coding	41	GC19M039834	0.099718325
FGF13	Fibroblast Growth Factor 13	Protein Coding	41	GC0XM138615	0.099718325
FMOD	Fibromodulin	Protein Coding	41	GC01M203340	0.099718325
GNL3	G Protein Nucleolar 3	Protein Coding	41	GC03P052681	0.099718325
GPAM	Glycerol-3-Phosphate Acyltransferase, Mitochondrial	Protein Coding	41	GC10M112148	0.099718325
HAX1	HCLS1 Associated Protein X-1	Protein Coding	41	GC01P154273	0.099718325
LGALS3BP	Galectin 3 Binding Protein	Protein Coding	41	GC17M078971	0.099718325
MAPRE2	Microtubule Associated Protein RP/EB Family Member 2	Protein Coding	41	GC18P034976	0.099718325
MNDA	Myeloid Cell Nuclear Differentiation Antigen	Protein Coding	41	GC01P158801	0.099718325
NDE1	NudE Neurodevelopment Protein 1	Protein Coding	41	GC16P017556	0.099718325
PCBP1	Poly(RC) Binding Protein 1	Protein Coding	41	GC02P070087	0.099718325
PIAS2	Protein Inhibitor Of Activated STAT 2	Protein Coding	41	GC18M046808	0.099718325
PLP1	Proteolipid Protein 1	Protein Coding	41	GC0XP103773	0.099718325
PSMD12	Proteasome 26S Subunit, Non-ATPase 12	Protein Coding	41	GC17M067337	0.099718325
PSMD9	Proteasome 26S Subunit, Non-ATPase 9	Protein Coding	41	GC12P126233	0.099718325
PUF60	Poly(U) Binding Splicing Factor 60	Protein Coding	41	GC08M143816	0.099718325
QKI	QKI, KH Domain Containing RNA Binding	Protein Coding	41	GC06P163414	0.099718325
RAD23A	RAD23 Homolog A, Nucleotide Excision Repair Protein	Protein Coding	41	GC19P014288	0.099718325
RBX1	Ring-Box 1	Protein Coding	41	GC22P040951	0.099718325
RCC1	Regulator Of Chromosome Condensation 1	Protein Coding	41	GC01P028505	0.099718325
RECQL4	RecQ Like Helicase 4	Protein Coding	41	GC08M145425	0.099718325
RPL10A	Ribosomal Protein L10a	Protein Coding	41	GC06P083826	0.099718325
RPS12	Ribosomal Protein S12	Protein Coding	41	GC06P132814	0.099718325
RPS24	Ribosomal Protein S24	Protein Coding	41	GC10P078033	0.099718325
RPS26	Ribosomal Protein S26	Protein Coding	41	GC12P056043	0.099718325
RPS27	Ribosomal Protein S27	Protein Coding	41	GC01P153991	0.099718325
SAMHD1	SAM And HD Domain Containing Deoxynucleoside Triphosphate Triphosphohydrolase 1	Protein Coding	41	GC20M036890	0.099718325
SDCCAG8	SHH Signaling And Ciliogenesis Regulator SDCCAG8	Protein Coding	41	GC01P243255	0.099718325
SKP1	S-Phase Kinase Associated Protein 1	Protein Coding	41	GC05M134148	0.099718325
TANK	TRAF Family Member Associated NFKB Activator	Protein Coding	41	GC02P161136	0.099718325
TRIB3	Tribbles Pseudokinase 3	Protein Coding	41	GC20P000361	0.099718325
USH1C	USH1 Protein Network Component Harmonin	Protein Coding	41	GC11M017594	0.099718325
CCNE2	Cyclin E2	Protein Coding	40	GC08M094879	0.099718325
CDK14	Cyclin Dependent Kinase 14	Protein Coding	40	GC07P090471	0.099718325
CLN8	CLN8 Transmembrane ER And ERGIC Protein	Protein Coding	40	GC08P001755	0.099718325
CNOT1	CCR4-NOT Transcription Complex Subunit 1	Protein Coding	40	GC16M058519	0.099718325
COLEC12	Collectin Subfamily Member 12	Protein Coding	40	GC18M000318	0.099718325
CYB5B	Cytochrome B5 Type B	Protein Coding	40	GC16P069474	0.099718325
EEF1D	Eukaryotic Translation Elongation Factor 1 Delta	Protein Coding	40	GC08M143579	0.099718325
FOXO4	Forkhead Box O4	Protein Coding	40	GC0XP071095	0.099718325
HMGB2	High Mobility Group Box 2	Protein Coding	40	GC04M173331	0.099718325
IFT88	Intraflagellar Transport 88	Protein Coding	40	GC13P020566	0.099718325
IL7	Interleukin 7	Protein Coding	40	GC08M078689	0.099718325
KCNAB1	Potassium Voltage-Gated Channel Subfamily A Regulatory Beta Subunit 1	Protein Coding	40	GC03P156037	0.099718325
MEST	Mesoderm Specific Transcript	Protein Coding	40	GC07P130486	0.099718325
PCBP2	Poly(RC) Binding Protein 2	Protein Coding	40	GC12P053452	0.099718325
PSMC4	Proteasome 26S Subunit, ATPase 4	Protein Coding	40	GC19P066660	0.099718325
PSMD11	Proteasome 26S Subunit, Non-ATPase 11	Protein Coding	40	GC17P032444	0.099718325
PSME1	Proteasome Activator Subunit 1	Protein Coding	40	GC14P024136	0.099718325
PSME3	Proteasome Activator Subunit 3	Protein Coding	40	GC17P042824	0.099718325
PYCARD	PYD And CARD Domain Containing	Protein Coding	40	GC16M031201	0.099718325
SLC25A6	Solute Carrier Family 25 Member 6	Protein Coding	40	GC0XM001386	0.099718325

SMARCD1	SWI/SNF Related, Matrix Associated, Actin Dependent Regulator Of Chromatin, Subfamily D, Member 1	Protein Coding	40	GC12P050085	0.099718325
TMED10	Transmembrane P24 Trafficking Protein 10	Protein Coding	40	GC14M075135	0.099718325
UBR5	Ubiquitin Protein Ligase E3 Component N-Recognin 5	Protein Coding	40	GC08M102252	0.099718325
AATF	Apoptosis Antagonizing Transcription Factor	Protein Coding	39	GC17P036948	0.099718325
CTNNBL1	Catenin Beta Like 1	Protein Coding	39	GC20P037693	0.099718325
DHRS2	Dehydrogenase/Reductase 2	Protein Coding	39	GC14P032711	0.099718325
DNAJA2	DnaJ Heat Shock Protein Family (Hsp40) Member A2	Protein Coding	39	GC16M046955	0.099718325
KIFC1	Kinesin Family Member C1	Protein Coding	39	GC06P033391	0.099718325
MPG	N-Methylpurine DNA Glycosylase	Protein Coding	39	GC16P011593	0.099718325
MPZL1	Myelin Protein Zero Like 1	Protein Coding	39	GC01P167721	0.099718325
MYBBP1A	MYB Binding Protein 1a	Protein Coding	39	GC17M004538	0.099718325
NEDD8	NEDD8 Ubiquitin Like Modifier	Protein Coding	39	GC14M024216	0.099718325
NFIC	Nuclear Factor I C	Protein Coding	39	GC19P003314	0.099718325
PSMC6	Proteasome 26S Subunit, ATPase 6	Protein Coding	39	GC14P052707	0.099718325
PSMD13	Proteasome 26S Subunit, Non-ATPase 13	Protein Coding	39	GC11P000236	0.099718325
PSME2	Proteasome Activator Subunit 2	Protein Coding	39	GC14M024143	0.099718325
RAB35	RAB35, Member RAS Oncogene Family	Protein Coding	39	GC12M120096	0.099718325
SCARA5	Scavenger Receptor Class A Member 5	Protein Coding	39	GC08M027869	0.099718325
SNRPA	Small Nuclear Ribonucleoprotein Polypeptide A	Protein Coding	39	GC19P040750	0.099718325
UBE2M	Ubiquitin Conjugating Enzyme E2 M	Protein Coding	39	GC19M058555	0.099718325
VRK3	VRK Serine/Threonine Kinase 3	Protein Coding	39	GC19M049976	0.099718325
ZNF462	Zinc Finger Protein 462	Protein Coding	39	GC09P106860	0.099718325
ABCF2	ATP Binding Cassette Subfamily F Member 2	Protein Coding	38	GC07M151211	0.099718325
ANGPTL7	Angiopoietin Like 7	Protein Coding	38	GC01P011189	0.099718325
BOK	BCL2 Family Apoptosis Regulator BOK	Protein Coding	38	GC02P241558	0.099718325
CCDC88C	Coiled-Coil Domain Containing 88C	Protein Coding	38	GC14M091271	0.099718325
CHCHD10	Coiled-Coil-Helix-Coiled-Coil-Helix Domain Containing 10	Protein Coding	38	GC22M023765	0.099718325
DDX10	DEAD-Box Helicase 10	Protein Coding	38	GC11P108569	0.099718325
GLCE	Glucuronic Acid Epimerase	Protein Coding	38	GC15P117546	0.099718325
ILKAP	ILK Associated Serine/Threonine Phosphatase	Protein Coding	38	GC02M238170	0.099718325
KRT6C	Keratin 6C	Protein Coding	38	GC12M052468	0.099718325
LYAR	Ly1 Antibody Reactive	Protein Coding	38	GC04M004289	0.099718325
MBNL1	Muscleblind Like Splicing Regulator 1	Protein Coding	38	GC03P152243	0.099718325
MYO9A	Myosin IXA	Protein Coding	38	GC15M071822	0.099718325
MYOG	Myogenin	Protein Coding	38	GC01M203083	0.099718325
NAT10	N-Acetyltransferase 10	Protein Coding	38	GC11P034105	0.099718325
NVL	Nuclear VCP Like	Protein Coding	38	GC01M224227	0.099718325
OSTF1	Osteoclast Stimulating Factor 1	Protein Coding	38	GC09P075088	0.099718325
PIEZO1	Piezo Type Mechanosensitive Ion Channel Component 1	Protein Coding	38	GC16M088715	0.099718325
PRICKLE2	Prickle Planar Cell Polarity Protein 2	Protein Coding	38	GC03M064079	0.099718325
PSMD10	Proteasome 26S Subunit, Non-ATPase 10	Protein Coding	38	GC0XM108084	0.099718325
PSMD6	Proteasome 26S Subunit, Non-ATPase 6	Protein Coding	38	GC03M063973	0.099718325
PSMF1	Proteasome Inhibitor Subunit 1	Protein Coding	38	GC20P001113	0.099718325
RPL23	Ribosomal Protein L23	Protein Coding	38	GC17M038847	0.099718325
RPL30	Ribosomal Protein L30	Protein Coding	38	GC08M098024	0.099718325
RPL3L	Ribosomal Protein L3 Like	Protein Coding	38	GC16M001943	0.099718325
RPS11	Ribosomal Protein S11	Protein Coding	38	GC19P049496	0.099718325
SRM	Spermidine Synthase	Protein Coding	38	GC01M011054	0.099718325
SRPK3	SRSF Protein Kinase 3	Protein Coding	38	GC0XP153776	0.099718325
SUMO2	Small Ubiquitin Like Modifier 2	Protein Coding	38	GC17M075165	0.099718325
TBPL1	TATA-Box Binding Protein Like 1	Protein Coding	38	GC06P133952	0.099718325
TGIF2	TGFB Induced Factor Homeobox 2	Protein Coding	38	GC20P036573	0.099718325
TMPRSS5	Transmembrane Serine Protease 5	Protein Coding	38	GC11M113687	0.099718325
TRIM44	Tripartite Motif Containing 44	Protein Coding	38	GC11P035684	0.099718325
UBE2E1	Ubiquitin Conjugating Enzyme E2 E1	Protein Coding	38	GC03P023805	0.099718325
WDR77	WD Repeat Domain 77	Protein Coding	38	GC01M111439	0.099718325
ACOT8	Acyl-CoA Thioesterase 8	Protein Coding	37	GC20M045841	0.099718325
ACSM3	Acyl-CoA Synthetase Medium Chain Family Member 3	Protein Coding	37	GC16P020610	0.099718325
AK8	Adenylate Kinase 8	Protein Coding	37	GC09M132725	0.099718325
ALKBH3	AlkB Homolog 3, Alpha-Ketoglutarate Dependent Dioxygenase	Protein Coding	37	GC11P043902	0.099718325
CCN2	Cellular Communication Network Factor 2	Protein Coding	37	GC06M131948	0.099718325
CHCHD2	Coiled-Coil-Helix-Coiled-Coil-Helix Domain Containing 2	Protein Coding	37	GC07M056101	0.099718325
CXCL9	C-X-C Motif Chemokine Ligand 9	Protein Coding	37	GC04M076001	0.099718325
GEMIN2	Gem Nuclear Organelle Associated Protein 2	Protein Coding	37	GC14P039114	0.099718325
GGA2	Golgi Associated, Gamma Adaptin Ear Containing, ARF Binding Protein 2	Protein Coding	37	GC16M023464	0.099718325
GLRX2	Glutaredoxin 2	Protein Coding	37	GC01M193065	0.099718325
GNLY	Granulysin	Protein Coding	37	GC02P085685	0.099718325
GORAB	Golgin, RAB6 Interacting	Protein Coding	37	GC01P170501	0.099718325
GPER1	G Protein-Coupled Estrogen Receptor 1	Protein Coding	37	GC07P002054	0.099718325
GPM6B	Glycoprotein M6B	Protein Coding	37	GC0XM013789	0.099718325
HMG20A	High Mobility Group 20A	Protein Coding	37	GC15P077420	0.099718325
HNRNPAB	Heterogeneous Nuclear Ribonucleoprotein A/B	Protein Coding	37	GC05P178204	0.099718325
KHDRBS3	KH RNA Binding Domain Containing, Signal Transduction Associated 3	Protein Coding	37	GC08P135457	0.099718325
KIFC3	Kinesin Family Member C3	Protein Coding	37	GC16M057758	0.099718325
MPP3	MAGUK P55 Scaffold Protein 3	Protein Coding	37	GC17M043800	0.099718325
NDRG2	NDRG Family Member 2	Protein Coding	37	GC14M021016	0.099718325
NOP58	NOP58 Ribonucleoprotein	Protein Coding	37	GC02P202265	0.099718325
PNOC	Prepronociceptin	Protein Coding	37	GC08P028316	0.099718325
PRAME	PRAME Nuclear Receptor Transcriptional Regulator	Protein Coding	37	GC22M022547	0.099718325
PSMD5	Proteasome 26S Subunit, Non-ATPase 5	Protein Coding	37	GC09M120815	0.099718325
RAB32	RAB32, Member RAS Oncogene Family	Protein Coding	37	GC06P146543	0.099718325
REEP3	Receptor Accessory Protein 3	Protein Coding	37	GC10P063521	0.099718325
RGS1	Regulator Of G Protein Signaling 1	Protein Coding	37	GC01P192575	0.099718325
RNF20	Ring Finger Protein 20	Protein Coding	37	GC09P101533	0.099718325
RPL37A	Ribosomal Protein L37a	Protein Coding	37	GC02P216498	0.099718325
RSL24D1	Ribosomal L24 Domain Containing 1	Protein Coding	37	GC15M055180	0.099718325
SACS	Sacsin Molecular Chaperone	Protein Coding	37	GC13M023288	0.099718325
SLC25A26	Solute Carrier Family 25 Member 26	Protein Coding	37	GC03P066120	0.099718325
TECRL	Trans-2,3-Enoyl-CoA Reductase Like	Protein Coding	37	GC04M064275	0.099718325
TMOD2	Tropomodulin 2	Protein Coding	37	GC15P051751	0.099718325
TOM1L2	Target Of Myb1 Like 2 Membrane Trafficking Protein	Protein Coding	37	GC17M017843	0.099718325
TSLP	Thymic Stromal Lymphopoietin	Protein Coding	37	GC05P111070	0.099718325
ABCF3	ATP Binding Cassette Subfamily F Member 3	Protein Coding	36	GC03P184186	0.099718325
APOBEC3C	Apolipoprotein B MRNA Editing Enzyme Catalytic Subunit 3C	Protein Coding	36	GC22P039014	0.099718325
ATF5	Activating Transcription Factor 5	Protein Coding	36	GC19P067134	0.099718325
CHMP3	Charged Multivesicular Body Protein 3	Protein Coding	36	GC02M086505	0.099718325
CPEB4	Cytoplasmic Polyadenylation Element Binding Protein 4	Protein Coding	36	GC05P173888	0.099718325
DRAP1	DR1 Associated Protein 1	Protein Coding	36	GC11P070012	0.099718325

GBP2	Guanylate Binding Protein 2	Protein Coding	36	GC01M089106	0.099718325
GLIPR2	GLI Pathogenesis Related 2	Protein Coding	36	GC09P047186	0.099718325
INTS7	Integrator Complex Subunit 7	Protein Coding	36	GC01M211940	0.099718325
LETMD1	LETM1 Domain Containing 1	Protein Coding	36	GC12P051047	0.099718325
LRRC8E	Leucine Rich Repeat Containing 8 VRAC Subunit E	Protein Coding	36	GC19P007888	0.099718325
MED22	Mediator Complex Subunit 22	Protein Coding	36	GC09M133338	0.099718325
MED27	Mediator Complex Subunit 27	Protein Coding	36	GC09M131860	0.099718325
MRPL18	Mitochondrial Ribosomal Protein L18	Protein Coding	36	GC06P159789	0.099718325
MTUS1	Microtubule Associated Scaffold Protein 1	Protein Coding	36	GC08M017643	0.099718325
ORC3	Origin Recognition Complex Subunit 3	Protein Coding	36	GC06P087590	0.099718325
PCBD2	Pterin-4 Alpha-Carbinolamine Dehydratase 2	Protein Coding	36	GC05P134904	0.099718325
PDE12	Phosphodiesterase 12	Protein Coding	36	GC03P057556	0.099718325
PLP2	Proteolipid Protein 2	Protein Coding	36	GC0XP049171	0.099718325
PNO1	Partner Of NOB1 Homolog	Protein Coding	36	GC02P068157	0.099718325
POLR2K	RNA Polymerase II, I And III Subunit K	Protein Coding	36	GC08P100150	0.099718325
PPHLN1	Periphilin 1	Protein Coding	36	GC12P042238	0.099718325
PPP1R17	Protein Phosphatase 1 Regulatory Subunit 17	Protein Coding	36	GC07P031726	0.099718325
RBM23	RNA Binding Motif Protein 23	Protein Coding	36	GC14M022902	0.099718325
RPL22L1	Ribosomal Protein L22 Like 1	Protein Coding	36	GC03M170864	0.099718325
RRP12	Ribosomal RNA Processing 12 Homolog	Protein Coding	36	GC10M097356	0.099718325
S100A13	S100 Calcium Binding Protein A13	Protein Coding	36	GC01M153618	0.099718325
S100A16	S100 Calcium Binding Protein A16	Protein Coding	36	GC01M153606	0.099718325
SEC16B	SEC16 Homolog B, Endoplasmic Reticulum Export Factor	Protein Coding	36	GC01M177923	0.099718325
TMEM107	Transmembrane Protein 107	Protein Coding	36	GC17M010442	0.099718325
TMEM237	Transmembrane Protein 237	Protein Coding	36	GC02M201620	0.099718325
UXT	Ubiquitously Expressed Prefoldin Like Chaperone	Protein Coding	36	GC0XM047651	0.099718325
YPEL5	Yippee Like 5	Protein Coding	36	GC02P030108	0.099718325
AMOTL2	Angiomotin Like 2	Protein Coding	35	GC03M134355	0.099718325
CKAP2	Cytoskeleton Associated Protein 2	Protein Coding	35	GC13P052455	0.099718325
DNAJB12	DnaJ Heat Shock Protein Family (Hsp40) Member B12	Protein Coding	35	GC10M072332	0.099718325
DNAJC30	DnaJ Heat Shock Protein Family (Hsp40) Member C30	Protein Coding	35	GC07M073680	0.099718325
GNL2	G Protein Nucleolar 2	Protein Coding	35	GC01M037566	0.099718325
GPKOW	G-Patch Domain And KOW Motifs	Protein Coding	35	GC0XM049113	0.099718325
IPO9	Importin 9	Protein Coding	35	GC01P201829	0.099718325
KIF12	Kinesin Family Member 12	Protein Coding	35	GC09M114086	0.099718325
MAP1S	Microtubule Associated Protein 1S	Protein Coding	35	GC19P066229	0.099718325
MRPS14	Mitochondrial Ribosomal Protein S14	Protein Coding	35	GC01M175010	0.099718325
MTIF3	Mitochondrial Translational Initiation Factor 3	Protein Coding	35	GC13M027436	0.099718325
POLDIP2	DNA Polymerase Delta Interacting Protein 2	Protein Coding	35	GC17M028346	0.099718325
PPP1R21	Protein Phosphatase 1 Regulatory Subunit 21	Protein Coding	35	GC02P048440	0.099718325
RRP1	Ribosomal RNA Processing 1	Protein Coding	35	GC21P043789	0.099718325
SLAIN1	SLAIN Motif Family Member 1	Protein Coding	35	GC13P077697	0.099718325
SRSF10	Serine And Arginine Rich Splicing Factor 10	Protein Coding	35	GC01M023964	0.099718325
STYX	Serine/Threonine/Tyrosine Interacting Protein	Protein Coding	35	GC14P052730	0.099718325
TAF9B	TATA-Box Binding Protein Associated Factor 9b	Protein Coding	35	GC0XM078129	0.099718325
XRN1	5'-3' Exoribonuclease 1	Protein Coding	35	GC03M142306	0.099718325
ANP32E	Acidic Nuclear Phosphoprotein 32 Family Member E	Protein Coding	34	GC01M150218	0.099718325
APOBEC4	Apolipoprotein B MRNA Editing Enzyme Catalytic Polypeptide Like 4	Protein Coding	34	GC01M183646	0.099718325
BRIX1	Biogenesis Of Ribosomes BRX1	Protein Coding	34	GC05P034916	0.099718325
CEP112	Centrosomal Protein 112	Protein Coding	34	GC17M065635	0.099718325
CILK1	Ciliogenesis Associated Kinase 1	Protein Coding	34	GC06M066350	0.099718325
CLIP3	CAP-Gly Domain Containing Linker Protein 3	Protein Coding	34	GC19M065729	0.099718325
DHX40	DEAH-Box Helicase 40	Protein Coding	34	GC17P059565	0.099718325
DOHH	Deoxyhypusine Hydroxylase	Protein Coding	34	GC19M005404	0.099718325
FYTTD1	Forty-Two-Three Domain Containing 1	Protein Coding	34	GC03P197737	0.099718325
HMG20B	High Mobility Group 20B	Protein Coding	34	GC19P003572	0.099718325
MRPL21	Mitochondrial Ribosomal Protein L21	Protein Coding	34	GC11M090212	0.099718325
NUDT16L1	Nudix Hydrolase 16 Like 1	Protein Coding	34	GC16P004693	0.099718325
ODF2L	Outer Dense Fiber Of Sperm Tails 2 Like	Protein Coding	34	GC01M086346	0.099718325
OLFML2A	Olfactomedin Like 2A	Protein Coding	34	GC09P124777	0.099718325
PAAF1	Proteasomal ATPase Associated Factor 1	Protein Coding	34	GC11P073876	0.099718325
PDCD7	Programmed Cell Death 7	Protein Coding	34	GC15M065117	0.099718325
PHLDA1	Pleckstrin Homology Like Domain Family A Member 1	Protein Coding	34	GC12M076025	0.099718325
PNRC2	Proline Rich Nuclear Receptor Coactivator 2	Protein Coding	34	GC01P024577	0.099718325
RGS22	Regulator Of G Protein Signaling 22	Protein Coding	34	GC08M099960	0.099718325
RPL36A	Ribosomal Protein L36a	Protein Coding	34	GC0XP101392	0.099718325
SPCS2	Signal Peptidase Complex Subunit 2	Protein Coding	34	GC11P077879	0.099718325
SPDYA	Speedy/RINGO Cell Cycle Regulator Family Member A	Protein Coding	34	GC02P028782	0.099718325
SRCIN1	SRC Kinase Signaling Inhibitor 1	Protein Coding	34	GC17M038530	0.099718325
TC2N	Tandem C2 Domains, Nuclear	Protein Coding	34	GC14M101154	0.099718325
WDR18	WD Repeat Domain 18	Protein Coding	34	GC19P002830	0.099718325
ZFYVE21	Zinc Finger FYVE-Type Containing 21	Protein Coding	34	GC14P103715	0.099718325
ZNF558	Zinc Finger Protein 558	Protein Coding	34	GC19M008806	0.099718325
AFDN	Afadin, Adherens Junction Formation Factor	Protein Coding	33	GC06P167827	0.099718325
AKIRIN2	Akirin 2	Protein Coding	33	GC06M087674	0.099718325
C15orf48	Chromosome 15 Open Reading Frame 48	Protein Coding	33	GC15P045430	0.099718325
FCF1	FCF1 RRNA-Processing Protein	Protein Coding	33	GC14P074713	0.099718325
GET4	Guided Entry Of Tail-Anchored Proteins Factor 4	Protein Coding	33	GC07P000876	0.099718325
KIAA1586	KIAA1586	Protein Coding	33	GC06P057046	0.099718325
LRRC20	Leucine Rich Repeat Containing 20	Protein Coding	33	GC10M070298	0.099718325
MRPL27	Mitochondrial Ribosomal Protein L27	Protein Coding	33	GC17M050367	0.099718325
MT-ND4	Mitochondrially Encoded NADH:Ubiquinone Oxidoreductase Core Subunit 4	Protein Coding	33	GCMTP010762	0.099718325
NKD2	NKD Inhibitor Of WNT Signaling Pathway 2	Protein Coding	33	GC05P001008	0.099718325
PIGY	Phosphatidylinositol Glycan Anchor Biosynthesis Class Y	Protein Coding	33	GC04M088520	0.099718325
RPS19BP1	Ribosomal Protein S19 Binding Protein 1	Protein Coding	33	GC22M057597	0.099718325
RRP15	Ribosomal RNA Processing 15 Homolog	Protein Coding	33	GC01P218285	0.099718325
SNX33	Sorting Nexin 33	Protein Coding	33	GC15P117709	0.099718325
SPATA4	Spermatogenesis Associated 4	Protein Coding	33	GC04M176184	0.099718325
TBKBP1	TBK1 Binding Protein 1	Protein Coding	33	GC17P047694	0.099718325
TEX10	Testis Expressed 10	Protein Coding	33	GC09M100302	0.099718325
TMEM147	Transmembrane Protein 147	Protein Coding	33	GC19P035545	0.099718325
YIPF6	Yip1 Domain Family Member 6	Protein Coding	33	GC0XP068498	0.099718325
ZBTB44	Zinc Finger And BTB Domain Containing 44	Protein Coding	33	GC11M130226	0.099718325
ZMYM5	Zinc Finger MYM-Type Containing 5	Protein Coding	33	GC13M019823	0.099718325
ZNF574	Zinc Finger Protein 574	Protein Coding	33	GC19P042068	0.099718325
ATAT1	Alpha Tubulin Acetyltransferase 1	Protein Coding	32	GC06P030626	0.099718325
H3-3B	H3.3 Histone B	Protein Coding	32	GC17M075809	0.099718325
HEATR3	HEAT Repeat Containing 3	Protein Coding	32	GC16P050065	0.099718325

LTV1	LTV1 Ribosome Biogenesis Factor	Protein Coding	32	GC06P143843	0.099718325
MIS18BP1	MIS18 Binding Protein 1	Protein Coding	32	GC14M045203	0.099718325
MRPL52	Mitochondrial Ribosomal Protein L52	Protein Coding	32	GC14P022829	0.099718325
MT-ND1	Mitochondrially Encoded NADH:Ubiquinone Oxidoreductase Core Subunit 1	Protein Coding	32	GCMTP003309	0.099718325
PRRC2B	Proline Rich Coiled-Coil 2B	Protein Coding	32	GC09P131373	0.099718325
RAB39A	RAB39A, Member RAS Oncogene Family	Protein Coding	32	GC11P107928	0.099718325
RBM33	RNA Binding Motif Protein 33	Protein Coding	32	GC07P155644	0.099718325
RWDD4	RWD Domain Containing 4	Protein Coding	32	GC04M183639	0.099718325
SLC7A6OS	Solute Carrier Family 7 Member 6 Opposite Strand	Protein Coding	32	GC16M068284	0.099718325
SREK1IP1	SREK1 Interacting Protein 1	Protein Coding	32	GC05M064718	0.099718325
SS18L2	SS18 Like 2	Protein Coding	32	GC03P042581	0.099718325
THAP7	THAP Domain Containing 7	Protein Coding	32	GC22M020999	0.099718325
TMEM120A	Transmembrane Protein 120A	Protein Coding	32	GC07M075986	0.099718325
TMEM134	Transmembrane Protein 134	Protein Coding	32	GC11M067461	0.099718325
TMEM14A	Transmembrane Protein 14A	Protein Coding	32	GC06P052671	0.099718325
ZC2HC1C	Zinc Finger C2HC-Type Containing 1C	Protein Coding	32	GC14P075064	0.099718325
ZNF684	Zinc Finger Protein 684	Protein Coding	32	GC01P040531	0.099718325
ASPHD1	Aspartate Beta-Hydroxylase Domain Containing 1	Protein Coding	31	GC16P041021	0.099718325
C1orf105	Chromosome 1 Open Reading Frame 105	Protein Coding	31	GC01P172419	0.099718325
CCDC60	Coiled-Coil Domain Containing 60	Protein Coding	31	GC12P119334	0.099718325
JADE2	Jade Family PHD Finger 2	Protein Coding	31	GC05P134524	0.099718325
MOB3C	MOB Kinase Activator 3C	Protein Coding	31	GC01M046607	0.099718325
PUSL1	Pseudouridine Synthase Like 1	Protein Coding	31	GC01P001308	0.099718325
ZKSCAN8	Zinc Finger With KRAB And SCAN Domains 8	Protein Coding	31	GC06P028141	0.099718325
ZNF688	Zinc Finger Protein 688	Protein Coding	31	GC16M030569	0.099718325
AKAP14	A-Kinase Anchoring Protein 14	Protein Coding	30	GC0XP119895	0.099718325
C2orf88	Chromosome 2 Open Reading Frame 88	Protein Coding	30	GC02P189879	0.099718325
DNAH14	Dynein Axonemal Heavy Chain 14	Protein Coding	30	GC01P224896	0.099718325
KCTD14	Potassium Channel Tetramerization Domain Containing 14	Protein Coding	30	GC11M090494	0.099718325
MOB3A	MOB Kinase Activator 3A	Protein Coding	30	GC19M005345	0.099718325
SFR1	SWI5 Dependent Homologous Recombination Repair Protein 1	Protein Coding	30	GC10P104120	0.099718325
TMEM128	Transmembrane Protein 128	Protein Coding	30	GC04M004298	0.099718325
C11orf16	Chromosome 11 Open Reading Frame 16	Protein Coding	29	GC11M008920	0.099718325
CMTR2	Cap Methyltransferase 2	Protein Coding	29	GC16M071281	0.099718325
MRM3	Mitochondrial RRNA Methyltransferase 3	Protein Coding	29	GC17P001547	0.099718325
TEX29	Testis Expressed 29	Protein Coding	29	GC13P111306	0.099718325
C1orf115	Chromosome 1 Open Reading Frame 115	Protein Coding	28	GC01P220689	0.099718325
CCDC186	Coiled-Coil Domain Containing 186	Protein Coding	28	GC10M114120	0.099718325
CLHC1	Clathrin Heavy Chain Linker Domain Containing 1	Protein Coding	28	GC02M055172	0.099718325
C22orf31	Chromosome 22 Open Reading Frame 31	Protein Coding	27	GC22M029058	0.099718325
ERG28	Ergosterol Biosynthesis 28 Homolog	Protein Coding	26	GC14M075650	0.099718325
ERICH2	Glutamate Rich 2	Protein Coding	26	GC02P170772	0.099718325
H2AZ2	H2A.Z Variant Histone 2	Protein Coding	26	GC07M044829	0.099718325
SELENOK	Selenoprotein K	Protein Coding	26	GC03M053884	0.099718325
ZUP1	Zinc Finger Containing Ubiquitin Peptidase 1	Protein Coding	26	GC06M116636	0.099718325
CFAP300	Cilia And Flagella Associated Protein 300	Protein Coding	25	GC11P102048	0.099718325
HPF1	Histone PARylation Factor 1	Protein Coding	25	GC04M169730	0.099718325
INKA2	Inka Box Actin Regulator 2	Protein Coding	25	GC01M111689	0.099718325
TENT5D	Terminal Nucleotidyltransferase 5D	Protein Coding	24	GC0XP080336	0.099718325
TSBP1	Testis Expressed Basic Protein 1	Protein Coding	24	GC06M032288	0.099718325
PGAP4	Post-GPI Attachment To Proteins GalNAc Transferase 4	Protein Coding	23	GC09M101476	0.099718325
MIR221	MicroRNA 221	RNA Gene	21	GC0XM045746	0.099718325
IGKV1-5	Immunoglobulin Kappa Variable 1-5	Protein Coding	20	GC02M090892	0.099718325
MIR335	MicroRNA 335	RNA Gene	20	GC07P130496	0.099718325
OR14K1	Olfactory Receptor Family 14 Subfamily K Member 1	Protein Coding	20	GC01P247738	0.099718325
GMCL2	Germ Cell-Less 2, Spermatogenesis Associated	Protein Coding	19	GC05M178189	0.099718325
DPY19L2P1	DPY19L2 Pseudogene 1	Pseudogene	18	GC07M035080	0.099718325
EP400P1	EP400 Pseudogene 1	Pseudogene	18	GC12P132085	0.099718325
KIRREL3-AS3	KIRREL3 Antisense RNA 3	RNA Gene	18	GC11P127002	0.099718325
ANKRD36BP1	Ankyrin Repeat Domain 36B Pseudogene 1	Pseudogene	17	GC01M168245	0.099718325
TCP10L3	T-Complex 10 Like 3, Pseudogene	Pseudogene	17	GC06M167373	0.099718325
TEX41	Testis Expressed 41	RNA Gene	17	GC02P144667	0.099718325
CRMA	Cardiomyocyte Maturation Associated LncRNA	RNA Gene	13	GC20M062545	0.099718325
LINC01483	Long Intergenic Non-Protein Coding RNA 1483	RNA Gene	13	GC17P069577	0.099718325
TBC1D3P2	TBC1 Domain Family Member 3 Pseudogene 2	Pseudogene	13	GC17M062264	0.099718325
