# Supplementary material for: Six mitophagy-related hub genes as peripheral blood biomarkers of Alzheimer’s disease and their immune cell infiltration correlation
Source: Front Neurosci. 2023 May 18;17:1125281. doi: 10.3389/fnins.2023.1125281 (PMC10232817; doi:10.3389/fnins.2023.1125281)
Supplement: Supplementary file 8 [file Table_3.pdf]

Table S3. Results of MRDEGs in AD

Gene Symbol	logFC	AveExpr	pvalue
APOO	-0.619882665	9.84357486	0.000151755
PFN2	-0.521890668	12.26691466	0.000231407
ITGA5	0.867020896	8.487539163	0.00044612
DHX57	-0.64673567	7.409472314	0.000499452
PCCB	-0.513501521	8.759470981	0.000578416
MTX2	-0.63693071	9.267304096	0.000680296
KIFC3	0.699412506	7.62334366	0.00074259
NDUFS4	-0.521118372	9.474948948	0.001006045
SLC12A7	0.54241963	9.883952894	0.001373062
CHST3	0.518441489	8.647352497	0.001453549
GDAP1	-1.071962828	6.411657509	0.001670493
DLAT	-0.518657316	8.295915071	0.001904713
SLC35E1	0.515442968	10.3243477	0.00206301
NNT	-0.510453783	8.746276805	0.002134326
C1QBP	-0.516528645	9.807812891	0.002928101
KCNAB1	-0.716722763	8.931570163	0.00293937
INF2	0.501240531	9.14840211	0.002959694
ITGB4	0.5165276	8.167565184	0.002992207
EPHA2	0.626637781	7.235949332	0.003765345
MON1B	0.778881611	7.151669189	0.003799116
TMEM14A	-0.607925389	10.5581058	0.004974276
SLC1A5	0.658792946	7.726408594	0.005327564
CD44	0.65107755	8.651959164	0.006274914
RCN2	-0.628539928	10.06994394	0.006631999
ACTR10	-0.511287363	10.4166995	0.007036802
NETO2	-0.540498684	8.717373011	0.007572552
FOXO4	0.750743638	7.571734989	0.008324405
MDH1	-0.664040224	11.31019154	0.009566321
PNOC	-0.669999987	6.630491097	0.009832654
ZNF787	0.590825462	6.170037726	0.009873631
VPS33A	-0.518530371	7.081472919	0.010646982
TFEB	0.519701381	8.673291924	0.011119716
PDE12	0.627780054	6.506752328	0.011660267
SUCLA2	-0.576242113	9.609239781	0.011806263
NUP93	-0.763601945	6.915817492	0.014412055
NUPR1	0.522611982	9.924027043	0.016778381
MRPS28	-0.598903097	8.864167513	0.016832093
FGF13	-0.65606224	10.66658334	0.016966985
GLRX5	-0.570207814	9.443701249	0.01720705
MSTN	-0.777788912	5.045352106	0.018641968
UQCRC1	-0.534198466	10.12228491	0.02016572
MYC	0.759013813	6.384080312	0.020352417
NDE1	0.522065721	9.338278721	0.020371461
RAB23	-0.550118544	6.857038573	0.024569064
PSMA3	-0.584923148	9.131003426	0.025927833
DAP3	-0.51987231	9.188371418	0.027471947
DNAJC3	0.518591085	6.306625567	0.029974999
ITGAX	0.618531337	7.30153075	0.030008134
CPA3	-0.713266853	6.662586351	0.030031372
NOS3	0.521423179	6.668004624	0.030410751
PPARG	0.545186617	4.996263871	0.034302238
HILPDA	0.510783111	9.309649557	0.046833606
MRPS15	-0.539867216	7.488055155	0.048900652

MRDEGs, Mitophagy-Related Differentially Expressed Genes; AD, Alzheimer's Disease.
