# Supplementary material for: Six mitophagy-related hub genes as peripheral blood biomarkers of Alzheimer’s disease and their immune cell infiltration correlation
Source: Front Neurosci. 2023 May 18;17:1125281. doi: 10.3389/fnins.2023.1125281 (PMC10232817; doi:10.3389/fnins.2023.1125281)
Supplement: Supplementary file 9 [file Table_4.pdf]

Table S4. Results of MRDEGs in this research

Gene Symbol	Ⓐ	Ⓑ	Ⓒ	Ⓓ	Ⓔ	Ⓕ	Ⓖ	Ⓗ	Ⓘ	Ⓙ
APOO	14		ACTR10			ACTR10			ACTR10	
PFN2	X	APOO				APOO		APOO	APOO	
ITGA5	17		C1QBP			C1QBP			C1QBP	
DHX57	11			CD44		CD44		CD44	CD44	CD44
PCCB	10		CHST3			CHST3				
MTX2	3			CPA3			CPA3	CPA3		
KIFC3	1			DAP3		DAP3			DAP3	
NDUFS4	2	DHX57				DHX57		DHX57		
SLC12A7	11	DLAT				DLAT			DLAT	DLAT
CHST3	13			DNAJC3		DNAJC3		DNAJC3		
GDAP1	1		EPHA2			EPHA2			EPHA2	
DLAT	X			FGF13		FGF13			FGF13	
SLC35E1	X			FOXO4		FOXO4			FOXO4	
NNT	8		GDAP1			GDAP1			GDAP1	
C1QBP	14			GLRX5		GLRX5			GLRX5	
KCNAB1	7				HILPDA					
INF2	14		INF2			INF2			INF2	
ITGB4	12		ITGA5			ITGA5		ITGA5	ITGA5	
EPHA2	16			ITGAX			ITGAX	ITGAX	ITGAX	ITGAX
MON1B	17		ITGB4			ITGB4			ITGB4	
TMEM14A	3		KCNAB1			KCNAB1				
SLC1A5	16	KIFC3				KIFC3		KIFC3		
CD44	2			MDH1		MDH1			MDH1	
RCN2	16		MON1B			MON1B		MON1B	MON1B	
ACTR10	1				MRPS15				MRPS15	
NETO2	8		MRPS28			MRPS28			MRPS28	
FOXO4	2			MSTN		MSTN			MSTN	
MDH1	2	MTX2				MTX2			MTX2	
PNOC	8			MYC			MYC	MYC	MYC	MYC
ZNF787	16			NDE1		NDE1			NDE1	
VPS33A	5		NDUFS4			NDUFS4			NDUFS4	
TFEB	16		NETO2			NETO2				
PDE12	5		NNT			NNT		NNT	NNT	
SUCLA2	7			NOS3		NOS3			NOS3	
NUP93	16			NUP93		NUP93		NUP93		
NUPR1	16			NUPR1		NUPR1				
MRPS28	3	PCCB				PCCB			PCCB	
FGF13	3		PDE12			PDE12		PDE12	PDE12	
GLRX5	3	PFN2				PFN2			PFN2	
MSTN	8				PNOC			PNOC		
UQCRC1	3				PPARG				PPARG	PPARG
MYC	14			PSMA3			PSMA3		PSMA3	
NDE1	6			RAB23		RAB23		RAB23		
RAB23	15		RCN2			RCN2			RCN2	
PSMA3	5		SLC12A7			SLC12A7				
DAP3	19		SLC1A5			SLC1A5			SLC1A5	
DNAJC3	19		SLC35E1			SLC35E1				
ITGAX	13			SUCLA2		SUCLA2			SUCLA2	SUCLA2
CPA3	6		TFEB			TFEB				
NOS3	6		TMEM14A			TMEM14A				
PPARG	3			UQCRC1		UQCRC1			UQCRC1	
HILPDA	12		VPS33A			VPS33A		VPS33A	VPS33A	
MRPS15	19			ZNF787		ZNF787		ZNF787		
Total	53	7	22	20	4	45	4	17	36	6

MRDEGs, Mitophagy-Related Differentially Expressed Genes;

Ⓐ, Chromosome localization of MRDEGs；

Ⓑ, MRDEGs with statistically significance (p <0.001) in different groups on histogram；

Ⓒ, MRDEGs with statistically significance ($p < 0.01$) in different groups；
Ⓓ, MRDEGs with statistically significance ($p < 0.05$) in different groups；
Ⓔ, MRDEGs with statistically significance ($p \geq 0.05$) in different groups；
Ⓕ, MRDEGs with a certain correlation ($0.7 < AUC < 0.9$) on ROC curves；
Ⓖ, MRDEGs with a low correlation ($0.5 < AUC < 0.7$) on ROC curves；
Ⓗ, MRDEGs in the LASSO diagnostic model；
Ⓘ, Related MRDEGs in PPI network；
Ⓙ, Mitophagy-related hub genes.
